# MATCAP1 preferentially binds an expanded tubulin conformation to generate detyrosinated and ΔC2 α-tubulin

**DOI:** 10.1038/s44318-026-00772-6

**Published:** 2026-04-13

**Authors:** Yang Yue, Takashi Hotta, Ryoma Ohi, Kristen J Verhey

**Affiliations:** Department of Cell & Developmental Biology, University of Michigan Medical School, Ann Arbor, MI USA

**Keywords:** Cell Adhesion, Polarity & Cytoskeleton, Post-translational Modifications & Proteolysis

## Abstract

Microtubules are cytoskeletal filaments with critical roles in cell division, cell motility, intracellular trafficking, and cilium function. In cells, subsets of microtubules are selectively marked by posttranslational modifications (PTMs) that control the ability of microtubule-associated proteins (MAPs) and molecular motors to engage microtubules. Detyrosination (ΔY) and ΔC2 are PTMs of α-tubulin wherein one or two residues, respectively, are enzymatically removed from the C-terminus of the protein. How specific patterns of PTMs are generated in cells is incompletely understood. Here, we use in vitro reconstitution assays to investigate the microtubule-binding behavior of metallopeptidase MATCAP1 and the mechanism by which it generates ΔY and ΔC2 modifications of α-tubulin. We demonstrate that MATCAP1 preferentially binds to microtubules composed of tubulin subunits in an expanded conformation, which can be induced by preventing β-tubulin GTP hydrolysis, taxol treatment, or kinesin-1 stepping. MATCAP1 exhibits a long dwell-time on microtubules and sequentially removes residues to generate ΔY-microtubules and ΔC2-microtubules. Thus, the lattice conformation of microtubules is a key factor that gates the binding and activity of MATCAP1.

## Synopsis

MATCAP1 is a writer of the tubulin code that proteolytically removes one or two residues from the C-terminus of α-tubulin to generate ΔY- and ΔC2-tubulin, respectively. This study reveals that MATCAP1 preferentially binds tubulin in an expanded conformation, and that its long dwell-time enables sequential generation of ΔY- and ΔC2-microtubules.

- Novel biosensors can visualize and quantify ∆Y-tubulin and ∆C2-tubulin modifications on individual microtubules with high spatiotemporal resolution.
- MATCAP1 directly generates both ∆Y- and ∆C2-microtubules in cells and in vitro.
- MATCAP1-mediated conversion of tyrosinated microtubules to the ∆Y state is faster than the subsequent conversion to the ∆C2 state.
- MATCAP1 exhibits higher binding to and enzymatic activity on an expanded microtubule lattice than a compacted lattice.

## Abstract (web summary)

An expanded lattice conformation of microtubules recruits the metallopeptidase MATCAP1 for sequential removal of the two C-terminal residues from α-tubulin.

## Graphical abstract

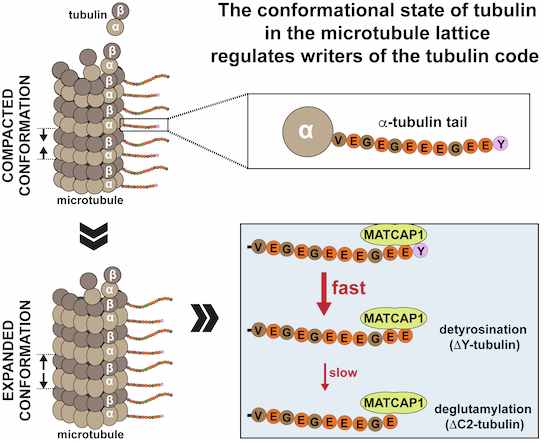

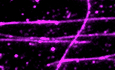

## Introduction

Microtubules are dynamic polymers of α,β-tubulin that play critical roles in eukaryotic cells, ranging from cell division to cell migration and intracellular trafficking. α,β-tubulin is a GTPase, and the nucleotide-bound state of β-tubulin is tied to the ability of microtubules to cycle between states of assembly and disassembly (Cleary and Hancock, 2021; Gudimchuk and McIntosh, 2021; Lawrence et al, 2023; Chew and Cross, 2025). In the GTP-bound form, tubulin readily assembles into microtubules. Upon incorporation into the microtubule lattice, β-tubulin hydrolyzes its bound nucleotide, generating GDP-bound β-tubulin. Cryo-electron microscopy studies show that GDP-tubulin is shortened (“compacted”) by ~1–2 Å along the longitudinal axis of the microtubule lattice relative to GTP-tubulin, which is said to be “expanded” (Alushin et al, 2014; Manka and Moores, 2018; Zhang et al, 2018; LaFrance et al, 2022). These nucleotide-dependent conformational states are thought to impact tubulin–tubulin interactions in the microtubule lattice and thereby affect microtubule dynamics.

In cells, the functional properties of microtubules are altered through post-translational modifications (PTMs) of their tubulin subunits, and these in turn act by changing the cohort of microtubule-associated proteins (MAPs) that interact with microtubules (Verhey and Gaertig, 2007; Janke and Magiera, 2020; Roll-Mecak, 2020). An important class of tubulin PTMs consists of those that involve proteolytic removal of amino acids from the α-tubulin C-terminal tail (αCTT): detyrosinated (ΔY)-, ΔC2-, and ΔC3-α-tubulin. Of these, microtubule detyrosination is the best understood in terms of its physiological roles and effects on MAPs (Nieuwenhuis and Brummelkamp, 2019; Ferreira et al, 2020; Moutin et al, 2021; Sanyal et al, 2023). For example, ΔY-microtubules near the poles of spindle microtubules facilitate chromosome congression by the kinesin-7 CENP-E (KIF10), whereas unmodified microtubules (tyrosinated or Y-microtubules) near the kinetochores enable proper kinesin-13 MCAK (KIF2C) error correction (Peris et al, 2009; Barisic et al, 2015; Liao et al, 2019). In interphase cells, the Y/ΔY state regulates intracellular trafficking as ΔY-microtubules are preferentially utilized by kinesin-1, whereas Y-microtubules are preferentially utilized by cytoplasmic dynein-1 (Dunn et al, 2008; Cai et al, 2009; Konishi and Setou, 2009; McKenney et al, 2016; Nirschl et al, 2016; Lavrsen et al, 2023; Konietzny et al, 2024). The Y/ΔY state also affects trafficking in cilia, where ΔY-microtubules promote the anterograde transport of intraflagellar transport trains by kinesin-2, and Y-microtubules enable the dynein-2-dependent retrograde trafficking of trains (Chhatre et al, 2025). In cardiomyocytes, microtubule detyrosination promotes a physical linkage between microtubules and sarcomeres, creating a force-restoring system that opposes myocyte contraction (Robison et al, 2016; Chen et al, 2018; Salomon et al, 2022).

How cells select microtubules for detyrosination or decide where to generate ΔC2- and ΔC3-α-tubulin has been a mystery for decades. Recent work has revealed that vertebrates encode two families of microtubule detyrosination enzymes: (1) the vasohibins (VASH1 and VASH2), cysteine proteases which require small-vasohibin binding protein (SVBP) as a cofactor (Aillaud et al, 2017; Nieuwenhuis et al, 2017) and (2) the microtubule-associated tyrosine carboxypeptidases (MATCAP1 and MATCAP2), metalloproteases also known as tubulin metallocarboxypeptidases (TMCP1 and TMCP2) (Landskron et al, 2022; Nicot et al, 2023) [reviewed in (Bak et al, 2024)]. Deletion of VASH1/2 and MATCAP1 proteins in mice and various cell lines abolishes the production of ΔY-microtubules (Landskron et al, 2022), suggesting that they are the only enzymes capable of microtubule detyrosination activity in mammals.

The writers of ΔC2- and ΔC3-α-tubulin marks have long been assumed to be cytosolic carboxypeptidases (CCPs) as these enzymes can generate ΔC2- and ΔC3-α-tubulin when overexpressed in mammalian cells (Rogowski et al, 2010; Berezniuk et al, 2012; Tort et al, 2014; Aillaud et al, 2016). Consistent with this possibility, we previously showed that CCP1 is the primary enzyme that generates ΔC2-α-tubulin in HeLa cells lacking tubulin tyrosine ligase (TTL), an enzyme that converts ΔY-α-tubulin back to full-length (tyrosinated) α-tubulin (Hotta et al, 2023). However, tissues from *pcd* mice, which harbor an inactivating mutation in CCP1, retain high levels of ΔC2-α-tubulin, suggesting that additional enzyme(s) can generate ΔC2-α-tubulin (Rogowski et al, 2010). Interestingly, recent work demonstrated that MATCAP1/TMCP1 and TMCP2 are both capable of generating ΔC2-α-tubulin in addition to ΔY-α-tubulin (Nicot et al, 2023).

In previous work, we developed a microscopy-based enzymatic assay to track the generation of ΔY-microtubules by VASH1/SVBP in real time. We discovered that VASH1/SVBP binds to microtubules regardless of their nucleotide or conformational state. However, the ability of VASH1/SVBP to detyrosinate microtubules is dramatically enhanced when tubulin subunits are in an expanded conformation, i.e., on microtubules assembled with the non-hydrolyzable analog GMPCPP or GDP-microtubules that are stabilized with the drug Taxol (Yue et al, 2023). These findings provide an explanation for why most microtubules in cells, which exist in a GDP-bound or compacted state, are not selected for modification by the VASH1/SVBP enzyme.

In this work, we sought to extend our understanding of the relationship between the conformational state of the microtubule lattice and detyrosination by examining the properties of MATCAP1. We find that, unlike VASH1/SVBP, MATCAP1 preferentially binds to microtubules with tubulin subunits in an expanded conformation, which can be induced by preventing β-tubulin GTP hydrolysis, Taxol treatment, or stepping of kinesin-1 along the microtubule lattice. Once bound, MATCAP1 exhibits a long dwell time, in contrast to VASH1/SVBP, which undergoes brief interactions with the microtubule lattice. Using probes that specifically recognize ΔY- and ΔC2-microtubules and the microscopy-based enzymatic assay, we show that MATCAP1’s rate of generating ΔY-microtubules is faster than for generating ΔC2-microtubules. This work provides a molecular explanation of how MATCAP1 sequentially generates ΔY- and ΔC2-α-tubulin within an expanded microtubule lattice.

## Results

### MATCAP1 directly generates both ΔY- and ΔC2-microtubules in HeLa cells

**Figure 1:**
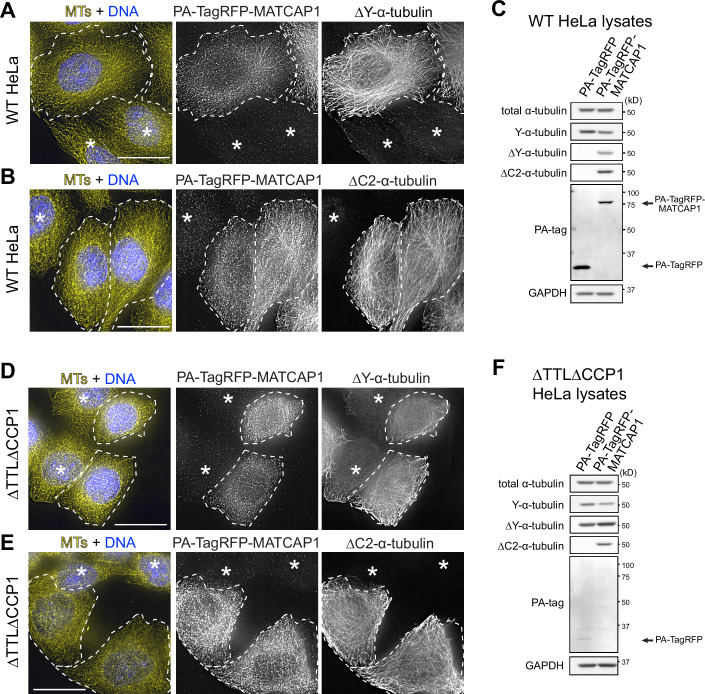
MATCAP1 directly generates both ΔY- and ΔC2-microtubules in cells. (**A**–**C**) Transient expression of MATCAP1 generates ΔY- and ΔC2-microtubules (MTs). (**A**, **B**) Representative images of HeLa cells transiently expressing PA-TagRFP-MATCAP1 and fixed and stained with antibodies against (**A**) ΔY-MTs or (**B**) ΔC2-MTs together with anti-PA-tag (to detect PA-TagRFP-MATCAP1) and AlexaFluor647-conjugated DM1a (to detect total MTs). Untransfected cells are indicated by an asterisk, and transfected cells are outlined with a dotted white line. Scale bars, 20 µm. (**C**) Lysates from HeLa cells transiently expressing PA-TagRFP or PA-TagRFP-MATCAP1 were immunoblotted with the indicated antibodies. (**D**–**F**) MATCAP1 directly generates ΔC2-MTs in cells. (**D**, **E**) Representative images of Δ*TTL*Δ*CCP1* HeLa cells transiently expressing PA-TagRFP-MATCAP1 and fixed and stained with antibodies against (**D**) ΔY-MTs or (**E**) ΔC2-MTs together with anti-PA-tag (to detect PA-TagRFP-MATCAP1) and AlexaFluor647-conjugated DM1a (to detect total MTs) antibodies. Untransfected cells are indicated by an asterisk, and transfected cells are outlined with a dotted white line. Scale bars, 20 µm. (**F**) Lysates from Δ*TTL*Δ*CCP1* HeLa cells transiently expressing PA-TagRFP or PA-TagRFP-MATCAP1 were immunoblotted with the indicated antibodies. Source data are available online for this figure.

**Figure EV1:**
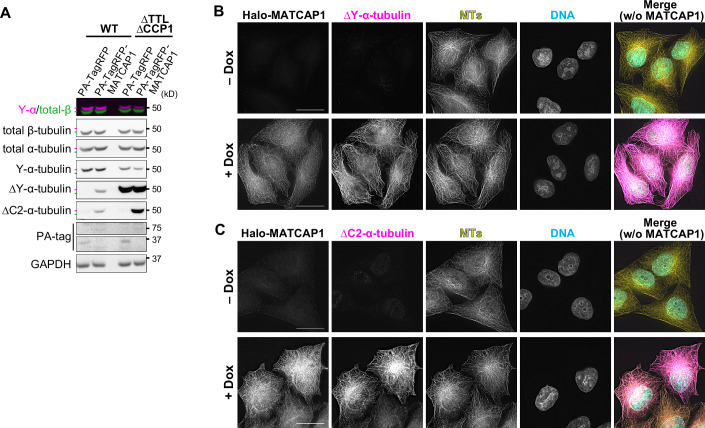
MATCAP1 generates both ΔY- and ΔC2-microtubules in cells. (**A**) Western blot of cell lysates from WT or Δ*TTL*Δ*CCP1* HeLa cells transiently expressing PA-TagRFP or PA-TagRFP-MATCAP1. The proteins were separated on high-pH gels to resolve α- and β-tubulin, and then the nitrocellulose membranes were blotted with antibodies against tyrosinated α-tubulin (Y-α, magenta) and β-tubulin (green) simultaneously or with antibodies against total α-tubulin, ΔY-α-tubulin, ΔC2-α-tubulin, the PA tag, and GAPDH. (**B**, **C**) Halo-MATCAP1 stable HeLa cells were untreated (-Dox) or treated with doxycycline ( + Dox) to induce Halo-MATCAP1 expression. Halo-MATCAP1 was labeled with JFX554 Halo ligand, and then cells were fixed and stained with antibodies against (**B**) ΔY-α-tubulin or (**C**) ΔC2-α-tubulin (magenta) and total α-tubulin (yellow). DNA is shown in cyan. Scale bars, 20 µm.

To investigate MATCAP1 activity in cells, we transiently expressed full-length human MATCAP1 tagged with both a PA epitope and TagRFP at the N-terminus (PA-TagRFP-MATCAP1) in HeLa Kyoto cells. No ΔY-microtubules were detected in the untransfected cells by immunostaining (Fig. 1A, asterisks) or in cells expressing the control PA-TagRFP construct by western blot (Figs. 1C and EV1A). However, transient expression of PA-TagRFP-MATCAP1 resulted in a dramatic increase in ΔY-microtubules by both immunostaining and western blot (Figs. 1A,C and EV1A). We also generated a HeLa Kyoto cell line that contains a stably integrated cassette for expressing Halo-MATCAP1 in a doxycycline-inducible manner. In the absence of doxycycline, no ΔY-microtubules were detected; however, doxycycline-induced expression of Halo-MATCAP1 resulted in a concomitant increase in ΔY-microtubules (Fig. EV1B). These results are consistent with previous work showing that the enzymatic activity of MATCAP1 results in tubulin detyrosination (Landskron et al, 2022; Nicot et al, 2023).

We next tested whether MATCAP1 activity results in increased levels of ΔC2-microtubules in cells. Indeed, we found that transient expression of PA-TagRFP-MATCAP1 (Fig. 1B,C) and doxycycline-induced expression of Halo-MATCAP1 (Fig. EV1C) in HeLa cells resulted in increased levels of ΔC2-microtubules, suggesting that MATCAP1 can carry out further trimming of the αCTT, in agreement with recent work (Nicot et al, 2023). ΔC2-microtubules in mammalian cells can also be generated by cytosolic carboxypeptidases (CCPs). While overexpression of five CCPs (CCP1, CCP2, CCP3, CCP4, and CCP6) can generate ΔC2-α-tubulin in cells, only loss of CCP1 decreases ΔC2-α-tubulin in cells and animals (Rogowski et al, 2010; Berezniuk et al, 2012; Tort et al, 2014; Hotta et al, 2023). To rule out the possibility that expression of MATCAP1 results in increased ΔC2-microtubules due to the activation of endogenous CCP1, we utilized a HeLa cell line (Δ*TTL*Δ*CCP1*) that lacks tubulin tyrosine ligase (TTL) and CCP1 proteins (Hotta et al, 2023). In the Δ*TTL*Δ*CCP1* cell line, knockout of TTL expression results in high levels of ΔY-microtubules (Figs. 1D, asterisks,  1F and EV1A), while knockout of CCP1 prevents the formation of ΔC2-α-tubulin as assessed by immunofluorescence (Fig. 1E, asterisks) and western blot (Figs. 1F and EV1A) (Hotta et al, 2023). Overexpression of MATCAP1 in Δ*TTL*Δ*CCP1* cells produced detectable ΔC2-microtubules, as assessed by both assays (Figs. 1E,F and EV1A–C). Taken together, these findings indicate that MATCAP1 can directly generate both ΔY-microtubules and ΔC2-microtubules, consistent with recent work (Nicot et al, 2023).

### MATCAP1 directly generates both ΔY-microtubules and ΔC2-microtubules in vitro

**Figure 2:**
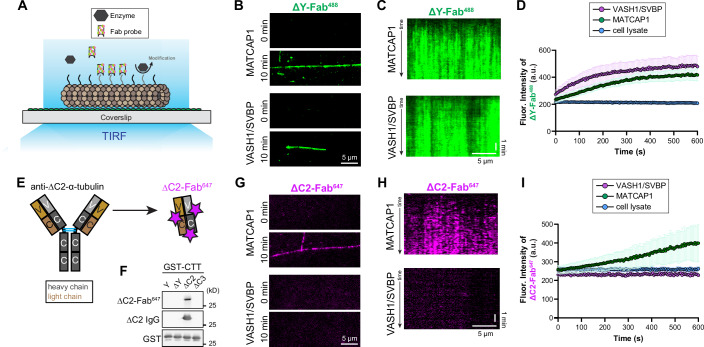
MATCAP1 directly generates both ΔY- and ΔC2-MTs in vitro. (**A**) Schematic of the microscopy-based enzymatic assay for detecting the biogenesis of microtubule PTMs. Taxol-stabilized HeLa MTs were incubated with a modifying enzyme (gray) and a fragment antibody binding (Fab) probe (multicolored) that recognizes a specific tubulin PTM. The binding of the Fab probe to microtubules is observed by total internal reflection fluorescence (TIRF) microscopy. (**B**–**D**) Biogenesis of ΔY-MTs. (**B**) Representative images of ΔY-Fab^488^ labeling of microtubules at 0 min and after 10 min incubation with 1 nM Halo-MATCAP1 or VASH1-Halo/SVBP in cell lysates. Scale bar, 5 µm. (**C**) Representative kymographs showing ΔY-Fab^488^ probe labeling of microtubules over time after the addition of 1 nM Halo-MATCAP1 or VASH1-Halo/SVBP in cell lysates. Time is shown on the *y* axis (scale bar, 1 min), and distance along the microtubule is on the *x* axis (scale bar, 5 µm). (**D**) Quantification of the mean fluorescence intensity of ΔY-Fab^488^ probe labeling along microtubules over time for the experiments in (**C**). Untransfected cell lysates were used as a control. Data are presented as mean ± SD, with *n* = 26–41 microtubules from two independent experiments. (**E**, **F**) Development of a Fab probe for ΔC2-MTs. (**E**) Schematic showing the RM447 monoclonal antibody against ΔC2-α-tubulin. The antibody was non-specifically labeled with Alexa647 dye, and then a Fab fragment (ΔC2-Fab^647^) was produced by papain digestion. (**F**) Purified glutathione S-transferase (GST) proteins fused to various α-tubulin C-terminal tail (CTT) sequences were analyzed by western blot using the ΔC2-Fab^647^ probe (top), the RM447 monoclonal ΔC2-α-tubulin antibody (middle), or an antibody against GST (bottom). The α-tubulin CTT sequences are full-length CTT (Y), missing the C-terminal tyrosine (ΔY), missing the C-terminal two amino acid residues (ΔC2), and missing the C-terminal three amino acid residues (ΔC3). (**G**–**I**) Biogenesis of ΔC2-MTs. (**G**) Representative images of ΔC2-Fab^647^ labeling of microtubules at 0 min and after 10 min incubation with 1 nM Halo-MATCAP1 or VASH1-Halo/SVBP in cell lysates. Scale bar, 5 µm. (**H**) Representative kymographs showing ΔC2-Fab^647^ probe labeling of microtubules over time after the addition of 1 nM Halo-MATCAP1 or VASH1-Halo/SVBP in cell lysates. Time is shown on the *y* axis (scale bar, 1 min), and distance along the microtubule is on the *x* axis (scale bar, 5  μm). (**I**) Quantification of the mean fluorescence intensity of ΔC2-Fab^647^ probe labeling along microtubules over time for the experiments shown in (**H**). Untransfected cell lysates were used as a control. Data are presented as mean ± SD, with *n* = 15–37 microtubules from two to four independent experiments. Source data are available online for this figure.

To investigate the catalytic properties of MATCAP1, we utilized a total internal reflection fluorescence (TIRF) microscopy-based enzymatic assay developed in our previous work on VASH1/SVBP (Yue et al, 2023). In this assay, Taxol-stabilized microtubules are adhered to the surface of flow chambers and then incubated with PTM enzymes and with a fluorescently labeled probe for detecting the modified tubulin (Fig. 2A). To avoid complications of PTM and isotype variability in brain tubulin, we purified tubulin from HeLa S3 cells as they lack most PTMs, including ΔY-tubulin (Souphron et al, 2019; Thomas et al, 2025). To compare the activities of MATCAP1 and VASH1/SVBP, we transiently expressed Halo-tagged versions of the enzymes in COS-7 cells and generated cell lysates, as this approach has been successful for investigating motors, MAPs, and PTM enzymes (e.g., Cai et al, 2007; Hammond et al, 2009; Jijumon et al, 2022; Yue et al, 2023).

**Figure EV2:**
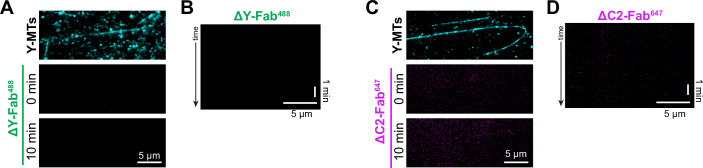
Controls using untransfected cell lysates in microscopy-based enzymatic assays. (**A**, **B**) Specificity of the ∆Y-Fab^488^ probe for microtubules in the presence of untransfected COS-7 cell lysates. (**A**) Representative images of ΔY-Fab^488^ probe labeling of Taxol-stabilized HeLa microtubules (cyan) at 0 min and after 10 min incubation with untransfected COS-7 cell lysates. Scale bar, 5 µm. (**B**) Representative kymographs showing ΔY-Fab^488^ probe labeling of microtubules over time after the addition of untransfected cell lysates. Time is shown on the *y* axis (scale bar, 1 min), and distance along the microtubule is on the *x* axis (scale bar, 5 µm). (**C**, **D**) Specificity of the ∆C2-Fab^647^ probe for microtubules in the presence of untransfected COS-7 cell lysates. (**C**) Representative images of ΔC2-Fab^647^ probe labeling of Taxol-stabilized HeLa microtubules (cyan) at 0 min and after 10 min incubation with untransfected cell lysates. Scale bar, 5 µm. (**D**) Representative kymographs showing ΔC2-Fab^647^ probe labeling of microtubules over time after the addition of untransfected cell lysates. Time is shown on the *y* axis (scale bar, 1 min), and distance along the microtubule is on the *x* axis (scale bar, 5 µm).

We first examined the generation of ΔY-microtubules. As a probe, we used a fluorescently labeled (Alexa488) fragment antibody binding (Fab) generated from the RM444 recombinant monoclonal antibody that specifically recognizes ΔY-α-tubulin [ΔY-Fab^488^ (Yue et al, 2023)]. Incubation of HeLa microtubules with cell lysates containing either MATCAP1 or VASH1/SVBP for 10 min resulted in a dramatic increase in the amount of ΔY-Fab^488^ probe bound to microtubules (Fig. 2B). We thus used time-lapse imaging to examine the kinetics of detyrosination by MATCAP1 and VASH1/SVBP using untransfected cell lysate as a control. When added at 1 nM in cell lysate, both enzymes resulted in rapid detyrosination of the microtubules (Fig. 2C,D) whereas incubation with untransfected cell lysates did not increase ΔY-Fab^488^ binding to microtubules over time (Figs. 2D and EV2A,B). These results indicate that the observed detyrosination is driven by the overexpressed MATCAP1 or VASH1/SVBP enzymes.

To examine the generation of ΔC2-microtubules, we generated a Fab version of the RM447 recombinant monoclonal antibody that specifically recognizes ΔC2-α-tubulin (Hotta et al, 2023). The ΔC2-Fab was fluorescently labeled with Alexa647 dye to generate a fluorescent ΔC2-Fab^647^ probe (Fig. 2E). We confirmed that the ΔC2-Fab^647^ probe shows the same selectivity for the ΔC2 α-tubulin CTT as the original IgG monoclonal antibody by western blotting (Fig. 2F). Incubation of Taxol-stabilized HeLa microtubules and the ΔC2-Fab^647^ probe with cell lysate containing MATCAP1 for 10 min resulted in a dramatic increase in ΔC2-Fab^647^ probe binding to microtubules (Fig. 2G). In contrast, incubation of HeLa microtubules and the ΔC2-Fab^647^ probe with either untransfected cell lysates or cell lysates containing VASH1/SVBP for 10 min did not increase ΔC2-Fab^647^ probe binding to microtubules (Figs. 2G and EV2C). Using time-lapse imaging of assays containing untransfected cell lysate, or cell lysate containing 1 nM MATCAP1 or VASH1/SVBP proteins, we found that only MATCAP1 activity resulted in an increase in ΔC2-microtubules over time (Figs. 2H,I and EV2D). Taken together, these results demonstrate that only MATCAP1 is capable of generating both ΔY-microtubules and ΔC2-microtubules in reconstitution assays, consistent with our observations in cells.

### MATCAP1 generates ΔY-microtubules faster than ΔC2-microtubules

**Figure EV3:**
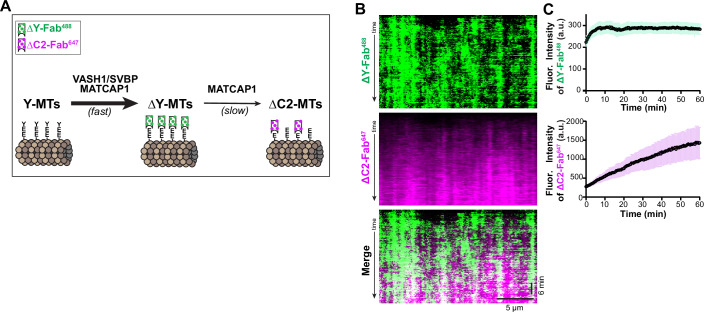
MATCAP1 generates ΔY-MTs faster than ΔC2-MTs in vitro. (**A**) Schematic illustrating the sequential cleavage of the α-tubulin CTT by MATCAP1 and probe binding. (**B**, **C**) Long imaging time visualizing the generation of the ΔY- and ΔC2-α-tubuilin modifications. (**B**) Representative kymographs showing ΔY-Fab^488^ (green) and ΔC2-Fab^647^ (magenta) labeling of Taxol-stabilized HeLa microtubules over 1 h incubation with 4.2 nM Halo-MATCAP1 in cell lysates. Time is shown on the *y* axis (scale bar, 6 min), and distance along the microtubule is on the *x* axis (scale bar, 5 µm). (**C**) Quantification of the mean fluorescence intensity of ΔY-Fab^488^ (green) and ΔC2-Fab^647^ (magenta) probes along microtubules over time. Data are presented as mean ± SD, with *n* = 100 microtubules from two independent experiments.

**Figure 3:**
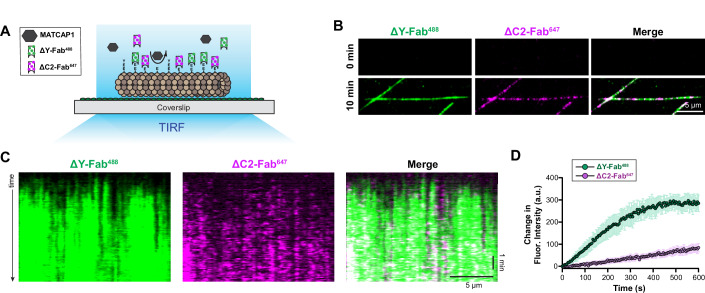
MATCAP1 generates ΔY-MTs faster than ΔC2-MTs in vitro. (**A**) Schematic of the microscopy-based enzymatic assay for dual Fab probe visualization of PTM biogenesis. Taxol-stabilized HeLa microtubules were incubated with MATCAP1, and the formation of ΔY-MTs and ΔC2-MTs was observed by Fab binding to the microtubules. (**B**) Representative images of ΔY-Fab^488^ (green) and ΔC2-Fab^647^ probe (magenta) labeling of the same microtubules at 0 min and after 10 min incubation with 1 nM Halo-MATCAP1 in cell lysates. Scale bar, 5 µm. (**C**, **D**) Simultaneous visualization of ΔY- and ΔC2-MT biogenesis. (**C**) Representative kymographs showing ΔY-Fab^488^ (green) and ΔC2-Fab^647^ probe (magenta) labeling of the same microtubule over time after the addition of 1 nM Halo-MATCAP1 in cell lysate. Time is shown on the *y* axis (scale bar, 1 min), and distance along the microtubule is on the *x* axis (scale bar, 5 µm). (**D**) Quantification of the change in fluorescence intensity of ΔY-Fab^488^ (green) and ΔC2-Fab^647^ (magenta) probes along microtubules over time. The data are presented as mean ± SD, with *n* = 29 microtubules from three independent experiments. Source data are available online for this figure.

A comparison of the graphs shows that in the presence of MATCAP1 enzyme, binding of the ΔY-Fab^488^ probe is faster than binding of the ΔC2-Fab^647^ probe (Fig. 2D vs  I). We hypothesized that the first step of the reaction (Fig. EV3A), cleavage of the Y to generate ΔY-α-tubulin, occurs quickly, whereas the second step of the reaction, cleavage of the E to generate ΔC2-α-tubulin, occurs at a much slower rate. To test this, we combined the ΔY-Fab^488^ and ΔC2-Fab^647^ probes in the same assay to directly compare the ability of MATCAP1 to generate these αCTT modifications. Taxol-stabilized HeLa microtubules were incubated with cell lysate containing 1 nM MATCAP1 protein in the presence of both the ΔY-Fab^488^ and the ΔC2-Fab^647^ probes (Fig. 3A). After a 10 min incubation, the ΔY-Fab^488^ and ΔC2-Fab^647^ probes were observed to decorate the same microtubules (Fig. 3B). We thus carried out time-lapse imaging and found that the ΔY-Fab^488^ probe rapidly labeled the microtubule whereas the labeling by the ΔC2-Fab^647^ probe appeared more slowly and was more sparse (Fig. 3C). Quantification of these assays showed that the ΔY-Fab^488^ fluorescence intensity plateaued 6–8 min after adding 1 nM MATCAP1, whereas the ΔC2-Fab^647^ intensity continued to increase throughout the 10 min imaging period (Fig. 3D). When the concentration of MATCAP1 was increased to 4.2 nM and the imaging time was extended to 1 h, the binding of the ΔC2-Fab^647^ probe to microtubules steadily increased over 1 h of imaging (Fig. EV3B,C). These results support the possibility that MATCAP1 sequentially modifies microtubules by rapidly generating ΔY-microtubules and more slowly generating ΔC2-microtubules (Fig. EV3A).

**Figure EV4:**
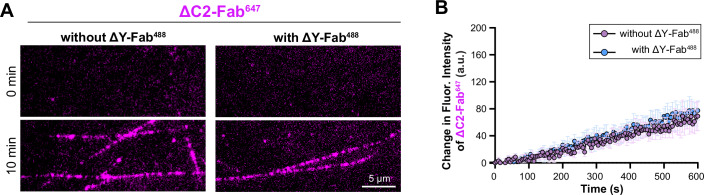
The ∆Y-Fab does not hinder the binding of the ∆C2-Fab. (**A**) Representative images of ΔC2-Fab^647^ labeling of Taxol-stabilized HeLa microtubules at 0 min and after 10 min incubation with 1 nM Halo-MATCAP1 in cell lysates, without or with ΔY-Fab^488^. Scale bar, 5 µm. (**B**) Quantification of the change in fluorescence intensity of ΔC2-Fab^647^ probe labeling along microtubules over time. The data are presented as mean  ± SD, with *n* = 17–23 microtubules from two independent experiments.

One concern with these assays stems from the fact that both Fab probes recognize the CTT of α-tubulin, which could lead to competition between the probes for substrate binding. To test whether the ΔY-Fab^488^ probe obstructs ΔC2-Fab^647^ binding, we compared ΔC2-microtubule biogenesis by MATCAP1 in the absence and presence of the ΔY-Fab^488^ probe. Our results show that the ΔY-Fab^488^ probe does not affect the kinetics of ΔC2-Fab^647^ microtubule labeling in response to MATCAP1 activity (Fig. EV4A,B), indicating that the ΔY-Fab^488^ does not hinder the binding of the ΔC2-Fab^647^.

**Figure EV5:**
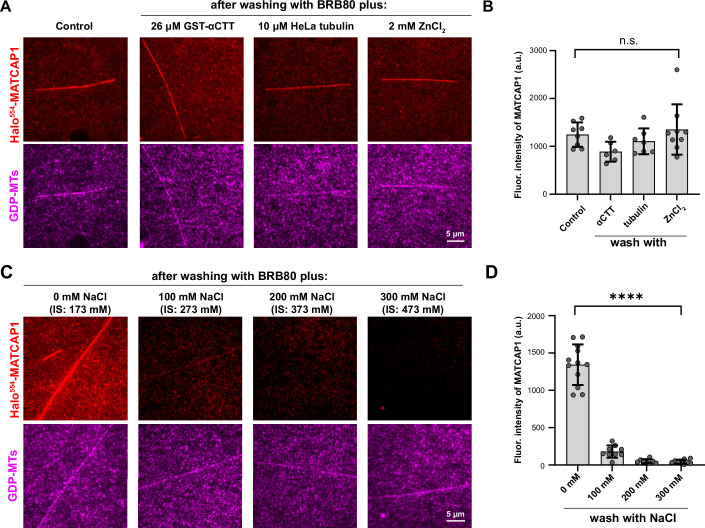
MATCAP1 detaches from microtubules in high ionic strength buffer. (**A**) Representative images of 1 nM Halo^554^-MATCAP1 (red) in cell lysates bound to glycerol-stabilized HeLa GDP-MTs (magenta) after washing with BRB80 containing 26 µM GST-αCTT, 10 µM HeLa tubulin, or 2 mM ZnCl_2_. Scale bar, 5 µm. (**B**) Quantification of the mean fluorescence intensity of Halo^554^-MATCAP1 under the indicated conditions is shown in (**A**). Data are presented as mean ± SD, with *n* = 6–9 microtubules. n.s., not significant (one-way ANOVA). (**C**) Representative images of 1 nM Halo^554^-MATCAP1 (red) in cell lysates bound to glycerol-stabilized HeLa GDP-MTs (magenta) after washing with BRB80 containing increasing concentrations of NaCl. IS Ionic strength. Scale bar, 5 µm. (**D**) Quantification of the mean fluorescence intensity of Halo^554^-MATCAP1 under the indicated conditions is shown in (**C**). Data are presented as mean ± SD, with *n* = 8–12 microtubules. ****P < 0.0001 (one-way ANOVA).

**Figure EV6:**
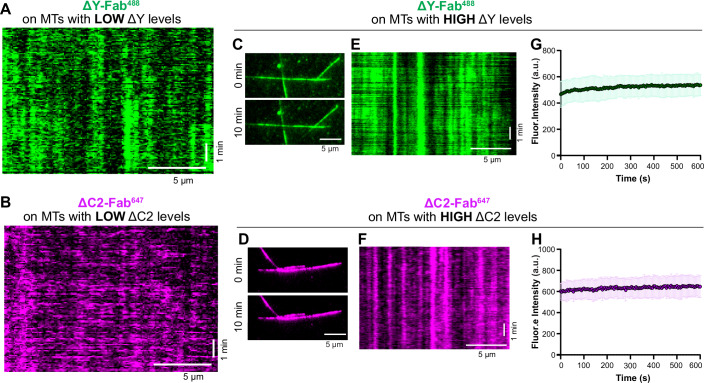
Controls for Fab binding to microtubules. (**A**, **B**) Single-molecule imaging of probe binding to pre-cleaved microtubules containing low levels of modification. Pre-cleaved microtubules were generated by incubating Taxol-stabilized HeLa microtubules with (**A**) 0.07 nM VASH1/SVBP in cell lysates for 2–3 s or (**B**) 0.7 nM MATCAP1 in cell lysates for 3 min to generate low levels of modification. The enzymes were then washed away with high-salt buffer, and the probes were added to the flow chamber and monitored over time. Representative kymographs are shown for (**A**) ΔY-Fab^488^ or (**B**) ΔC2-Fab^647^ probe labeling. Time is shown on the *y* axis (scale bar, 1 min) and distance along the microtubule is on the *x* axis (scale bar, 5 µm). (**C**–**H**) Probe binding to pre-cleaved microtubules containing high levels of modification. Pre-modified microtubules were generated by incubation with (**C**, **E**, **G**) 0.7 nM VASH1/SVBP in cell lysates for 2–3 s or (**D**, **F**, **H**) 1.4 nM MATCAP1 in cell lysates for 15 min. The enzymes were then washed away with high-salt buffer, followed by the addition of the Fab probes to the flow chamber. (**C**, **D**) Representative images of (**C**) ΔY-Fab^488^ or (**D**) ΔC2-Fab^647^ probe labeling of pre-cleaved microtubules at 0 min and after 10 min addition of the respective Fab probe. Scale bar, 5 µm. (**E**, **F**) Representative kymographs of (**E**) ΔY-Fab^488^ or (**F**) ΔC2-Fab^647^ probe labeling of pre-cleaved HeLa microtubules over time. Time is shown on the *y* axis (scale bar, 1 min), and distance along the microtubule is on the *x* axis (scale bar, 5 µm). (**G**, **H**) Quantification of the mean fluorescence intensity of (**G**) ΔY-Fab^488^ probe and (**H**) ΔC2-Fab^647^ probe along microtubules. Data are presented as mean ± SD, with *n* = 13–21 microtubules from two independent experiments.

A second concern with these assays is that the slower binding of the ΔC2-Fab^647^ probe reflects the probe’s binding kinetics rather than MATCAP1 enzymatic activity. To examine this, we measured the binding kinetics of the probes using pre-cleaved microtubules. To do this, we tested various conditions for removing the enzymes after modification of the microtubules. Whereas VASH1/SVBP could be detached from the microtubules by washing the flow chamber with BRB80 buffer (Yue et al, 2023), MATCAP1 removal required supplementing the BRB80 wash buffer with NaCl (Fig. EV5A–D). Therefore, we treated Taxol-stabilized HeLa microtubules with VASH1/SVBP to generate ΔY-microtubules or with MATCAP1 to generate ΔC2-microtubules. The enzymes were removed by a buffer wash, and then the ΔY-Fab^488^ or the ΔC2-Fab^647^ probe was added to the flow chamber. On microtubules with low levels of modification (generated using low enzyme concentrations and/or short incubation time), the ΔY-Fab^488^ and ΔC2-Fab^647^ probes showed rapid binding and unbinding events (Fig. EV6A,B), suggesting that the dwell time of an individual Fab is relatively short. On highly modified microtubules (generated using high enzyme concentrations and/or long incubation time), the probes appeared to stably decorate microtubules (Fig. EV6C–F), which likely reflects the binding of multiple probes to α-tubulin CTTs within each pixel. On highly modified microtubules, both the ΔY-Fab^488^ and ΔC2-Fab^647^ probes rapidly reached saturation levels (Fig. EV6G,H). Taken together, these results indicate that both probes have a high binding rate to the corresponding modified microtubule, which validates their utilization for real-time visualization of microtubule modification.

### Microtubule detyrosination status has minimal effect on MATCAP1 binding and activity

**Figure 4:**
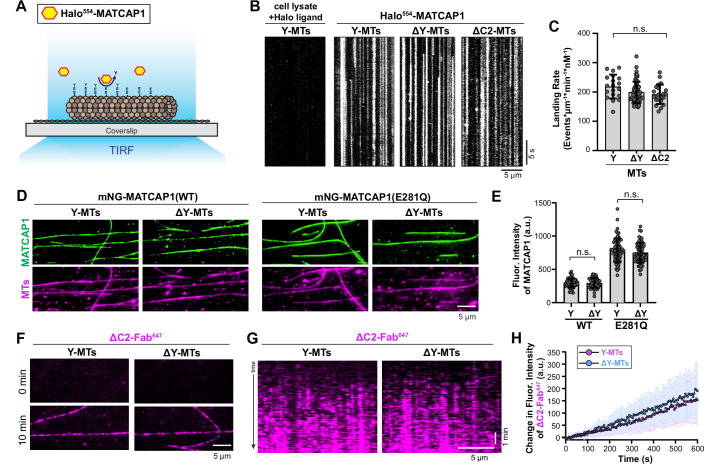
Microtubule detyrosination status has minimal effect on MATCAP1 binding and activity. (**A**) Schematic of the in vitro microtubule-binding assay using TIRF microscopy. Microtubules were incubated with varying concentrations of fluorescently labeled MATCAP1. Single-molecule binding events were monitored over time at low concentrations of MATCAP1, while the steady-state binding was analyzed by measuring fluorescence intensity at high concentrations of MATCAP1. (**B**, **C**) Landing rate of MATCAP1 on microtubules with different α-tubulin CTT modifications. Taxol-stabilized HeLa microtubules were untreated (Y-MTs) or were incubated with cell lysates containing VASH1/SVBP to generate ΔY-MTs or with MATCAP1 to generate ΔC2-MTs. The enzymes were removed with a high-salt wash, and then the cell lysate containing Halo^554^-MATCAP1 was added to the flow chamber. (**B**) Representative kymographs showing single molecules of 10 pM Halo^554^-MATCAP1 in cell lysate binding to the indicated microtubules. Untransfected cell lysates with Halo JFX554 ligand were used as a control. Time is shown on the *y* axis (scale bar, 5 s) and distance along the microtubule is on the *x* axis (scale bar, 5 µm). (**C**) Quantification of the landing rate of Halo^554^-MATCAP1 along microtubules. Each point represents the landing rate of an individual microtubule. Data are presented as mean ± SD, with *n* = 19–60 microtubules from two or three independent experiments. n.s., not significant (one-way ANOVA). (**D**, **E**) Steady-state binding of MATCAP1 to Y-MTs vs ΔY-MTs. (**D**) Representative images of 1 nM mNG-MATCAP1(WT) or mNG-MATCAP1(E281Q) in cell lysates binding to Taxol-stabilized Y- or ∆Y-MTs. Scale bar, 5 µm. (**E**) Quantification of mean fluorescence intensity of mNG-MATCAP1(WT) and mNG-MATCAP1(E281Q) along the microtubules in (**D**). Data are presented as mean ± SD, with *n *= 60–66 microtubules from two independent experiments. n.s., not significant (two-tailed, *t* test). (**F**–**H**) MATCAP1 activity along Y-MTs and ΔY-MTs. (**F**) Representative images of ΔC2-Fab^647^ labeling of microtubules at 0 min and after 10 min incubation with 1 nM Halo-MATCAP1 in cell lysate. Scale bar, 5 µm. (**G**) Representative kymographs showing ΔC2-Fab^647^ probe labeling over time. Time is shown on the *y* axis (scale bar, 1 min), and distance along the microtubule is on the *x* axis (scale bar, 5 µm). (**H**) Quantification of the change in fluorescence intensity of ΔC2-Fab^647^ probe along the microtubules in (**G**). Data are presented as mean ± SD, with *n* = 24 microtubules from two independent experiments. Source data are available online for this figure.

To understand how the state of the microtubule lattice impacts MATCAP1’s enzymatic activity, we characterized MATCAP1 binding to microtubules with different tubulin states using a microscopy-based microtubule binding assay (Fig. 4A). Halo-MATCAP1 was expressed in COS-7 cells and labeled with the JFX554 Halo ligand. The resulting cell extracts were added to Taxol-stabilized HeLa microtubules to observe the behavior of MATCAP1.

We first examined how modification of the α-tubulin CTT affects the binding of MATCAP1 to microtubules at the single-molecule level. Taxol-stabilized HeLa microtubules were untreated to maintain the tyrosinated state of α-tubulin or were treated with cell lysate containing VASH1/SVBP to generate ΔY-microtubules or with cell lysate containing MATCAP1 to generate ΔC2-microtubules. After washing out the enzymes with high-salt buffer (Fig. EV5), Halo^554^-MATCAP1 in cell lysates was added to the flow chamber and imaged over time. When added at 10 pM, individual Halo^554^-MATCAP1 molecules bound to Y-microtubules, ΔY-microtubules, and ΔC2-microtubules with comparable landing rates (Fig. 4B,C). As a control, untransfected cell lysate containing Halo JFX554 ligand was added to Taxol-stabilized Y-microtubules. No microtubule labeling was observed (Fig. 4B), indicating that the binding events are specific to Halo^554^-MATCAP1. Once bound to Taxol-stabilized microtubules, Halo^554^-MATCAP1 rarely detached, with a dwell time >30 s (the duration of our imaging period). This is in contrast to VASH1/SVBP, which rapidly binds and unbinds from microtubules (dwell time ~1 s (Yue et al, 2023; Ramirez-Rios, 2023)).

To further examine the binding of MATCAP1 to tyrosinated vs detyrosinated microtubules, we took advantage of a catalytically inactive version of MATCAP1 (E281Q), which localizes to microtubules in cells (Landskron et al, 2022). We compared the microtubule binding behavior of WT and E281Q MATCAP1 along Y-microtubules vs ΔY-microtubules. For these experiments, MATCAP1 was labeled with an mNeonGreen tag at the N-terminus (mNG-MATCAP1) to rule out any influence of the fluorescent tag on MATCAP1 behavior. When added at 1 nM in cell lysates, mNG-MATCAP1(WT) and mNG-MATCAP1(E281Q) showed no significant difference in binding to Y-microtubules vs ΔY-microtubules (Fig. 4D,E). Taken together, these results indicate that MATCAP1 binding to microtubules is not influenced by the detyrosination state of the α-tubulin CTT.

To examine whether the detyrosination state of the microtubule affects the activity of MATCAP1, we used the ΔC2-Fab^647^ probe to follow the generation of ΔC2-microtubules. Taxol-stabilized HeLa microtubules were untreated (Y-microtubules) or were treated with VASH1/SVBP to generate ΔY-microtubules. After a high-salt wash to remove VASH1/SVBP, cell lysate containing 1 nM MATCAP1 was added together with the ΔC2-Fab^647^ probe. The fluorescence intensity of ΔC2-Fab^647^ increased on both Y-microtubules and ΔY-microtubules (Fig. 4F). Using time-lapse imaging to follow the generation of ΔC2-tubulin in real time, MATCAP1’s ΔC2 modification activity appeared similar along Y- and ΔY-microtubules at the beginning of the reaction (Fig. 4G,H), largely due to the low signal caused by the rapid binding and unbinding of individual probes (Fig. EV6A). After ~100 sec, an increase in ΔC2 modification could be detected on ΔY-microtubules earlier than on Y-microtubules (Fig. 4G,H). The lag in generation of ΔC2-α-tubulin on Y-microtubules compared to ΔY-microtubules is consistent with a sequential reaction in which MATCAP1 quickly cleaves the terminal Y residue to generate ΔY-microtubules and then more slowly cleaves the E residue to generate ΔC2-microtubules (Fig. EV3A).

### Microtubule binding of MATCAP1 depends on the conformational state of tubulin

**Figure 5:**
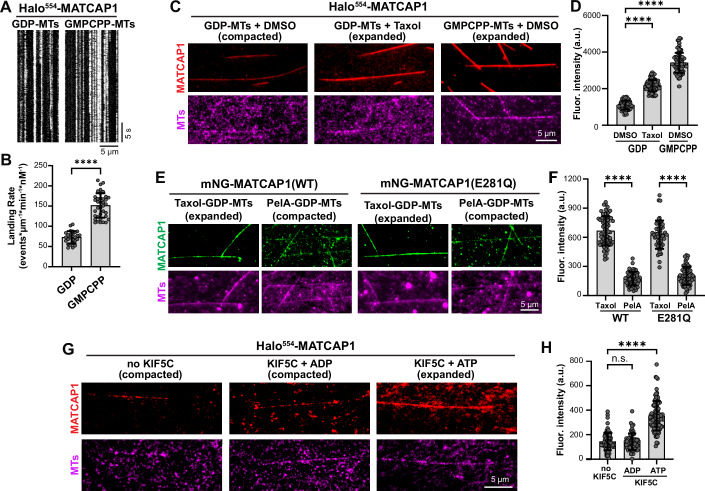
Microtubule binding of MATCAP1 is regulated by the conformational state of tubulin within the microtubule lattice. (**A**, **B**) Landing rate of Halo^554^-MATCAP1 on GDP-MTs vs GMPCPP-MTs. (**A**) Representative kymographs showing single molecules of 10 pM Halo^554^-MATCAP1 in cell lysates binding to glycerol-stabilized GDP-MTs or GMPCPP-MTs. Time is on the *y* axis (scale bar, 5 s), and distance along the microtubule is on the *x* axis (scale bar, 5 µm). (**B**) Quantification of the landing rate of Halo^554^-MATCAP1 along the microtubules in (**A**). Each point represents the landing rate of an individual microtubule. Data are presented as mean ± SD, with *n* = 26–37 microtubules from two independent experiments. *P* = 5.127E-18. ****P < 0.001 (two-tailed, *t* test). (**C**, **D**) Steady-state binding of MATCAP1 to compacted vs expanded microtubules. (**C**) Representative images of 1 nM Halo^554^-MATCAP1 in cell lysates binding to glycerol-stabilized GDP-MTs with DMSO, GDP-MTs with Taxol, or GMPCPP-MTs with DMSO. Scale bar, 5 µm. (**D**) Quantification of the mean fluorescence intensity of Halo^554^-MATCAP1 along microtubules. Data are presented as mean ± SD, with *n* = 56–83 microtubules from three independent experiments. *P* = 3.084E-41 (GDP-MTs, DMSO vs. GDP-MTs, Taxol) and *P* = 1.147E-61 (GDP-MTs, DMSO vs. GMPCPP-MTs, DMSO). ****P < 0.0001 (two-tailed, *t* test). (**E**, **F**) Steady-state binding of MATCAP1 to Taxol-stabilized vs PelA-stabilized GDP-MTs. (**E**) Representative images of 2 nM mNG-MATCAP1(WT) or mNG-MATCAP1(E281Q) in cell lysates binding to Taxol- or PelA-stabilized GDP-MTs. Scale bar, 5 µm. (**F**) Quantification of the mean fluorescence intensity of mNG-MATCAP1(WT) and mNG-MATCAP1(E281Q) along microtubules. Data are presented as mean ± SD with *n* = 51–71 microtubules from two independent experiments. *P* = 4.153E-53 (WT). *P *= 1.085E-34 (E281Q). ****P < 0.0001 (two-tailed, *t* test). (**G**, **H**) Steady-state binding of MATCAP1 to KIF5C-expanded microtubules. (**G**) Representative images of 3 nM purified Halo^554^-MATCAP1 protein binding to glycerol-stabilized GDP-MTs in the absence of KIF5C (microtubule compacted), in the presence of 100 nM KIF5C and 2 mM ADP (KIF5C inactive, microtubule compacted) or in the presence of 100 nM KIF5C and 2 mM ATP (KIF5C active, microtubule expanded). (**H**) Quantification of the mean fluorescence intensity of Halo^554^-MATCAP1 along microtubules. Data are presented as mean ± SD, with *n* = 60–100 microtubules from two or three independent experiments. *P* = 0.94 (no 5 C vs. 5 C with ADP). *P* = 7.671E-32 (no 5 C vs. 5C with ATP). ****P < 0.0001 and n.s., not significant (two-tailed, *t* test). Source data are available online for this figure.

We next examined how the conformational state of tubulin in the microtubule lattice affects the binding and activity of MATCAP1. HeLa tubulin was polymerized in the presence of the nonhydrolyzable GTP analog GMPCPP (GMPCPP-microtubules) or in the presence of GTP to generate GDP-microtubules as GTP is converted to GDP upon polymerization. The microtubules were stabilized by glycerol, and low concentrations (10 pM) of Halo^554^-MATCAP1 in the cell lysate were added. Individual Halo^554^-MATCAP1 molecules exhibited long dwell times on both types of microtubules (Fig. 5A). We thus calculated the landing rate as the number of pre-bound molecules and those binding de novo during imaging and found that the landing rate was higher on GMPCPP-microtubules than on GDP-microtubules (Fig. 5B). These results suggest that microtubule binding of MATCAP1 is regulated by the nucleotide state of tubulin within the microtubule lattice. This behavior is in contrast to that of VASH1/SVBP, which displays similar landing rates on GMPCPP- and GDP-microtubules (Yue et al, 2023).

The higher landing rate on GMPCPP-microtubules suggests that MATCAP1 may preferentially bind to tubulin within the microtubule lattice that is in an expanded state. To test this, we took advantage of the fact that Taxol can revert GDP-tubulin to an expanded state within the microtubule lattice (Alushin et al, 2014; Prota et al, 2023). We compared the fluorescence intensity of Halo^554^-MATCAP1 in cell lysate along GDP-microtubules with DMSO (compacted state), GDP-microtubules with Taxol (expanded state), and GMPCPP-microtubules with DMSO (expanded state). Halo^554^-MATCAP1 showed 2.1-fold higher binding to Taxol-treated GDP-microtubules than to GDP-microtubules and 3.4-fold higher binding to GMPCPP-microtubules than to GDP-microtubules (Fig. 5C,D).

**Figure EV7:**
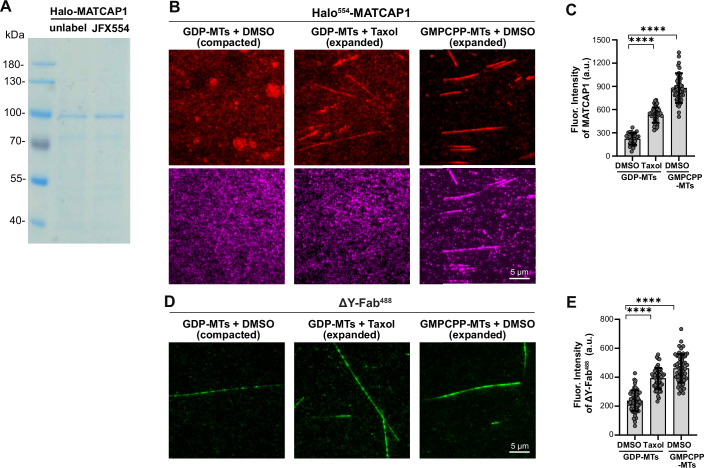
The microtubule binding and detyrosination activity of purified MATCAP1 are affected by the conformational state of the microtubule. (**A**) Coomassie-stained gel of TwinStrep-Halo-MATCAP1 protein purified from COS-7 cells. (**B**, **C**) Microtubule binding of purified MATCAP1 is regulated by the conformational state of tubulin in the microtubule lattice. (**B**) Representative images of 1.5 nM purified TwinStrep-Halo^554^-MATCAP1 protein binding to glycerol-stabilized GDP-MTs with DMSO, GDP-MTs with Taxol, or GMPCPP-MTs with DMSO. Scale bar, 5 µm. (**C**) Quantification of the mean fluorescence intensity of TwinStrep-Halo^554^-MATCAP1 along the microtubules in (**B**). Each point represents the mean fluorescence intensity of an individual microtubule. Data are presented as mean ± SD, with *n* = 30–56 microtubules from two or three independent experiments. *P* = 1.893E-22 (GDP-MTs, DMSO vs. GDP-MTs, Taxol). *P* = 1.330E-30 (GDP-MTs, DMSO vs. GMPCPP-MTs, DMSO). ****P < 0.0001 (two-tailed, *t* test). (**D**, **E**) Detyrosination activity of purified MATCAP1 is regulated by the conformational state of tubulin in the microtubule lattice. (**D**) Representative images of ΔY-Fab^488^ labeling of glycerol-stabilized GDP-MTs with DMSO, GDP-MTs with Taxol, or GMPCPP-MTs with DMSO after incubation with 1.5 nM purified Halo-MATCAP1 protein. MATCAP1 was removed by a high-salt wash step before the addition of the ΔY-Fab^488^ probe. Scale bar, 5 µm. (**E**) Quantification of the mean fluorescence intensity of the ΔY-Fab^48^ probe labeling along the microtubules in (**D**). Each point represents the mean fluorescence intensity of an individual microtubule. Data are presented as mean ± SD, with *n* = 52–65 microtubules from two or three independent experiments. *P* = 7.098E-21 (GDP-MTs, DMSO vs. GDP-MTs, Taxol). *P* = 2.317E-28 (GDP-MTs, DMSO vs. GMPCPP-MTs, DMSO). ****P < 0.0001 (two-tailed, *t* test).

Given this key finding, we confirmed the results using purified MATCAP1 protein. To do this, we added a dual Strep tag to Halo-MATCAP1 (TwinStrep-Halo-MATCAP1) and purified the protein from COS-7 cells using affinity chromatography (Fig. EV7A). The purified TwinStrep-Halo^554^-MATCAP1 protein showed 2.4-fold higher binding to Taxol-treated GDP-microtubules than to GDP-microtubules and 3.9-fold higher binding to GMPCPP-microtubules than to GDP-microtubules (Fig. EV7B,C).

As a second test of whether MATCAP1 preferentially binds to tubulins in an expanded state, we compared its ability to bind to GDP-microtubules stabilized with Taxol vs peloruside A (hereafter referred to as PelA). Although both natural products stabilize GDP-microtubules, only Taxol drives tubulin to an expanded state in the microtubule lattice (Huzil et al, 2008; Estevez-Gallego et al, 2023). For these experiments, GDP-microtubules were polymerized from HeLa tubulin, stabilized with either Taxol or PelA, and then incubated with cell lysate containing 2 nM mNG-MATCAP1(WT) or mNG-MATCAP1(E281Q) in an imaging chamber. The fluorescence intensity of mNG-MATCAP1 was 3.8-fold higher on Taxol-GDP-microtubules compared to PelA-GDP-microtubules (Fig. 5E,F). Similarly, the E281Q mutant protein showed 3.1-fold higher fluorescence intensity on Taxol-GDP-microtubules than on PelA-GDP-microtubules (Fig. 5E,F). These results demonstrate that both WT and E281Q MATCAP1 proteins display preferential binding to tubulin in an expanded state within the microtubule lattice.

**Figure EV8:**
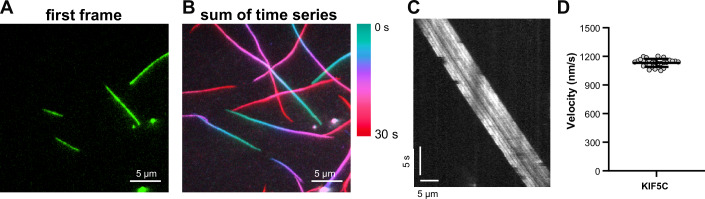
Motility properties of purified kinesin-1 protein in microtubule gliding assays. (**A**, **B**) Representative images. 200 nM unlabeled purified kinesin-1 protein was attached to the flow cells, and then Taxol-stabilized microtubules were introduced in the presence of ATP, and images were acquired over time. (**A**) First frame of imaging. (**B**) Time-lapse projection. Color bar, imaging time. Scale bars, 5 µm. (**C**) Representative kymograph of an individual microtubule gliding over time. Time is shown on the *y* axis (scale bar, 5 s), and distance along the microtubule is on the *x* axis (scale bar, 5 µm). (**D**) Quantification of the velocity of microtubule gliding driven by purified kinesin-1 protein. Data are presented as mean ± SD, with *n* = 28 microtubules.

Finally, we used the kinesin-1 motor KIF5C as an alternative, non-drug-based mechanism to drive GDP-tubulin to an expanded state within the microtubule lattice. Previous work has shown that kinesin-1 triggers lattice expansion when it is in the ATP-bound (active) state but not in the ADP-bound (inactive) state (Peet et al, 2018; Shima et al, 2018). We purified KIF5C(1-560)-Halo-TwinStrep from insect cells and verified its motility via a microtubule gliding assay (Fig. EV8A–D). Then, we measured the fluorescence intensity of MATCAP1 protein along GDP-microtubules in the absence or presence of purified KIF5C protein. To rigorously demonstrate these key findings, we used purified TwinStrep-Halo^554^-MATCAP1 protein for these experiments. In the presence of active KIF5C (ATP-bound), the mean fluorescence intensity of TwinStrep-Halo^554^-MATCAP1 along the microtubule lattice was increased by 2.5-fold compared to the absence of KIF5C (Fig. 5G,H). In contrast, the presence of inactive KIF5C (ADP-bound) did not affect TwinStrep-Halo^554^-MATCAP1 binding to microtubules compared to the absence of KIF5C (Fig. 5G,H).

Collectively, these results demonstrate that the microtubule binding of MATCAP1 is regulated by the conformational state of tubulin within the microtubule lattice. This behavior contrasts with that of VASH1/SVBP, which binds equally to microtubules regardless of tubulin conformation (Yue et al, 2023).

### Preferential binding to expanded tubulin determines the ability of MATCAP1 to detyrosinate tubulin in the microtubule lattice

**Figure 6:**
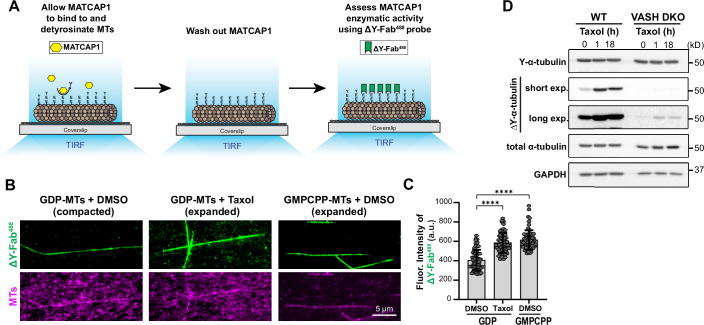
Detyrosination activity of MATCAP1 is regulated by the conformational state of tubulin within the microtubule lattice. (**A**) Schematic of the modified microscopy-based enzymatic assay. Glycerol-stabilized HeLa microtubules were incubated with MATCAP1 (yellow) in cell lysate for microtubule detyrosination. The enzyme was removed by high-salt wash buffer, and then the ΔY-Fab^488^ probe was added to visualize microtubule detyrosination. (**B**, **C**) The detyrosination activity of MATCAP1 on compacted vs. expanded microtubules. (**B**) Representative images of ΔY-Fab^488^ labeling of glycerol-stabilized GDP-MTs with DMSO, GDP-MTs with Taxol, or GMPCPP-MTs with DMSO after incubation with 1 nM Halo-MATCAP1 in cell lysates. Scale bar, 5 µm. (**C**) Quantification of the mean fluorescence intensity of ΔY-Fab^488^ probe labeling along the microtubules in (**B**). Each point represents the mean fluorescence intensity of an individual microtubule. Data are presented as mean ± SD, with *n* = 61–65 microtubules from three independent experiments. *P* = 2.926E-16 (GDP-MTs, DMSO vs. GDP-MTs, Taxol). *P* = 6.678E-21 (GDP-MTs, DMSO vs. GMPCPP-MTs, DMSO). ****P < 0.0001 (two-tailed, *t* test). (**D**) MATCAP1 activity is regulated by microtubule lattice expansion in cells. CHL-1 wild-type (WT) and VASH1/2 double knockout (DKO) cells were treated with 10 µM Taxol for 0, 1, or 18 h and then immunoblotted with the indicated antibodies. The signal from the ΔY-α-tubulin antibody is shown after a short exposure and long exposure. Source data are available online for this figure.

Given the preferential binding of MATCAP1 to an expanded microtubule lattice, we examined whether its detyrosination activity also depends on the conformational state of tubulin in the microtubule lattice. Halo-MATCAP1 in cell lysate was incubated for 6 min with GDP-microtubules with DMSO (compacted state), GDP-microtubules with Taxol (expanded state), or GMPCPP-microtubules with DMSO (expanded state). Bound MATCAP1 was then removed by washing with high-salt buffer. Microtubules were subsequently incubated with the ΔY-Fab^488^ probe to visualize the extent of detyrosination catalyzed by MATCAP1 (Fig. 6A). We found that MATCAP1 exhibited higher detyrosination activity on GMPCPP-microtubules and Taxol-treated GDP-microtubules than on GDP-microtubules (Fig. 6B,C). To confirm these key findings, we carried out similar experiments with purified TwinStrep-Halo-MATCAP1 protein. We found that purified Halo-MATCAP1 displays higher detyrosination activity along microtubules with an expanded lattice (Fig. EV7D,E). These results suggest that the detyrosination activity of MATCAP1 depends on the conformational state of tubulin within the microtubule lattice.

To examine whether MATCAP1 activity is influenced by the expanded state of the microtubule lattice in cells, we utilized ΔVASH1/ΔVASH2 double knockout (VASH DKO) CHL-1 cells, which lack expression of both VASH1 and VASH2 detyrosination enzymes and display a dramatic decrease in the levels of ΔY-microtubules (Fig. 6D), consistent with (Nieuwenhuis et al, 2017). WT and VASH DKO cells were treated with 10 µM Taxol to generate an expanded GDP-lattice, and the levels of detyrosinated microtubules were assessed by Western blot. The levels of ΔY-tubulin increased dramatically after 1 h Taxol treatment of WT (parental) CHL-1 cells (Fig. 6D). In contrast, the levels of ΔY-tubulin did not appear to increase in the VASH DKO cells even after 18 h Taxol treatment (Fig. 6D). These results suggest that endogenous VASH enzymes are the major contributors of microtubule detyrosination in CHL-1 cells, consistent with previous work (Nieuwenhuis et al, 2017). However, a longer exposure of the blot allowed us to detect a subtle increase in the levels of ΔY-tubulin in VASH DKO cells treated with Taxol for 1 or 18 h (Fig. 6D). Given that the VASH and MATCAP enzymes are the only detyrosinases known to date, the Taxol-dependent detyrosination activity in VASH DKO cells is most likely due to MATCAP activity. This finding is consistent with previous reports on HAP1, HEK293T, U2OS, and HCT116 cells deficient for VASH1 and VASH2 (Nieuwenhuis et al, 2017; Landskron et al, 2022). Overall, our results indicate that the expanded state of the microtubule lattice is a key feature that gates microtubule detyrosination by MATCAP1.

## Discussion

How cells select specific microtubules for tubulin PTMs remains a major unresolved question in the field. In this study, we provide mechanistic insights into the microtubule binding and enzymatic activity of MATCAP1. By developing microscopy-based enzymatic assays with PTM-specific Fab probes, we discovered that MATCAP1 binds preferentially to microtubules with an expanded lattice to carry out the sequential generation of ΔY- and ΔC2-α-tubulin modifications. Our findings provide insights into how the conformational state of tubulin subunits within the microtubule lattice participates in the spatiotemporal regulation of tubulin PTMs.

A significant challenge in studying tubulin PTMs has been the lack of tools for real-time detection, limiting our ability to understand how specific PTMs are dynamically established and regulated. To address this, we previously developed a fluorescently labeled Fab probe for ΔY-microtubules (Yue et al, 2023). In this study, we applied the same approach to generate a fluorescently labeled Fab probe specific for ΔC2-microtubules. Both Fab probes exhibit immediate and selective binding to their respective targets, enabling real-time visualization of ΔY and ΔC2 modifications on individual microtubules. Furthermore, both Fab probes exhibit rapid and transient binding kinetics and thus do not interfere with each other’s labeling under experimental conditions, enabling the simultaneous tracking of both PTMs. Using the dual-Fab strategy, we were able to directly demonstrate that MATCAP1 catalyzes the formation of ΔY-tubulin faster than ΔC2-tubulin on the same microtubule. Collectively, our work establishes Fab-based probes as powerful tools for spatiotemporal analysis of microtubule PTMs, opening new avenues to investigate the functional crosstalk among distinct tubulin PTMs.

We demonstrate that MATCAP1 efficiently generates both ΔY- and ΔC2-microtubules in vitro and in cells, consistent with recent work (Nicot et al, 2023). However, it had remained unclear whether MATCAP1 acts independently of CCP1 to generate ΔC2-microtubules in cells. Here, by overexpressing MATCAP1 in Δ*TTL*Δ*CPP1* HeLa cells, we demonstrate that MATCAP1 is capable of directly generating ΔC2-α-tubulin. This cell-based evidence is corroborated by our in vitro enzymatic assays, which reveal that MATCAP1 efficiently generates the ΔC2 modification along microtubules.

The ability of MATCAP1 to generate both ΔY- and ΔC2-α-tubulin modifications may be facilitated by its relatively long dwell time (>30 s) on microtubules. In this respect, MATCAP1 behavior is more similar to VASH2/SVBP (dwell time >11 s) than to VASH1/SVBP (dwell time ~1 s) (Ramirez-Rios et al, 2023; Yue et al, 2023). It seems possible that MATCAP1 could remain bound to a single tubulin substrate and sequentially modify its αCTT. This possibility is supported by structural studies showing that MATCAP1 interacts with αCTT residues N-terminal to the cleavage site and is able to carry out a variety of cleavage events at the C-terminal end of the αCTT (Landskron et al, 2022). However, we cannot exclude the possibility that individual MATCAP1 proteins diffuse with a diffraction-limited area to proteolyze the CTTs of multiple tubulins before falling off of the microtubule.

We find that the microtubule binding of MATCAP1 is insensitive to the prior detyrosination state of tubulin in the microtubule lattice, similar to VASH1/SVBP and VASH2/SVBP (Ramirez-Rios et al, 2023; Yue et al, 2023). These findings rule out a model in which progressive modification occurs through a positive feedback mechanism caused by increased affinity of the enzyme for its modified substrate. Rather, we find that MATCAP1 binds preferentially to microtubules with an expanded lattice conformation, which can be induced by preventing β-tubulin GTP hydrolysis, Taxol treatment, or stepping of active kinesin-1 along the microtubule. This behavior is different than that of VASH1/SVBP, which binds equally to microtubules that are in a compacted vs expanded state (Yue et al, 2023). The molecular basis for this difference is not readily apparent from recent cryo-EM data. VASH1/SVBP binds in between protofilaments and engages the two adjacent α-tubulin subunits, whereas MATCAP1 binds along one protofilament of a microtubule, primarily contacting the α-tubulin subunit targeted for cleavage while making contacts with an adjacent β-tubulin subunit (Li et al, 2020; Landskron et al, 2022; Ramirez-Rios et al, 2023). Thus, both MATCAP1 and VASH1/SVBP make extensive contacts with α-tubulin beyond its CTT, yet only MATCP1’s microtubule binding is sensitive to the compacted vs expanded state of tubulins in the lattice. Further structural studies are needed to understand the interactions of full-length MATCAP1 and VASH1/SVBP proteins along microtubules in different conformational states.

We also find that MATPCAP1 has a higher affinity for GMPCPP microtubules than for Taxol-stabilized microtubules. Although both of these conditions generate an expanded tubulin state, there are differences between them that could affect MATCAP1 binding. For example, GMPCPP microtubules display a greater degree of tubulin expansion compared to Taxol-stabilized microtubules (83.2 Å vs 82.3 Å, respectively) (Alushin et al, 2014). In addition, GMPCPP-microtubules predominantly contain 14 protofilaments and exhibit a high degree of positive twist (~+0.2°), whereas Taxol-stabilized microtubules predominantly contain 13 protofilaments and have a more moderate degree of positive twist (~+0.1°) (Alushin et al, 2014; Zhang et al, 2015; Manka and Moores, 2018; LaFrance et al, 2022). In addition to MATCAP1, these features are likely to impact the binding and/or activity of other MAPs and PTM enzymes. Indeed, recent work suggests that the acetylation activity of α-tubulin acetyl transferase (αTAT1) is higher on GMPCPP-microtubules than on Taxol-stabilized microtubules (Egoldt et al, 2025). Therefore, further investigation is needed to determine the features of an expanded microtubule lattice that regulate the microtubule binding and activity of microtubule-modifying enzymes.

Our finding that the detyrosination activities of MATCAP1 (this study) and VASH1/SVBP (Yue et al, 2023) are regulated by the conformational state of tubulin within the microtubule lattice is consistent with emerging studies showing that tubulin conformation can influence the binding affinity and activity of various MAPs and vice versa. For example, kinesin-1, CAMSAP3, and MAP7 preferentially bind to expanded microtubules, and their binding can further promote lattice expansion (Peet et al, 2018; Shima et al, 2018; Liu and Shima, 2023; Shen and Ori-McKenney, 2024). In contrast, Doublecortin (DCX), MAP2, and tau, preferentially bind to and induce lattice compaction (Castle et al, 2020; Siahaan et al, 2022; Paquette et al, 2025). Collectively, our findings support a model in which MAPs that modulate tubulin conformation within the microtubule lattice contribute to the regulation of tubulin PTMs.

## Methods

**Table:** Reagents and tools table

Reagent/resource	Reference or source	Identifier or catalog #
**Experimental models**		
HeLa Kyoto cells	Shuh Narumiya	RRID: CVCL_1922
∆TTL∆CCP1 HeLa cells	Hotta et al, 2023	N/A
Stable Halo-MATCAP1 HeLa cells	This study	N/A
COS-7	ATCC	RRID: CVCL_0224
Wild-type CHL-1 cells	Nieuwenhuis et al, 2017	RRID: CVCL_1122
∆VASH1/2 double knockout CHL-1 cells	Nieuwenhuis et al, 2017	N/A
DH5α	ThermoFisher	Cat# 18258-012
DH10Bac Escherichia coli	ThermoFisher	Cat# 10361012
Sf9 cells	Yue et al, 2018	N/A
**Recombinant DNA**		
pC1-Halo-MATCAP1	This study	N/A
pC1-mNG-MATCAP1	This study	N/A
pC1-PA-TagRFP-MATCAP1	This study	N/A
pC1-TwinStrep-Halo-MATCAP1	This study	N/A
E281Q pC1-mNG-MATCAP1	This study	N/A
VASH1-Halo-IRES-SVBP	Yue et al, 2023	N/A
pFastBac1-KIF5C(1-560)]-Halo-TwinStrepII	This study	N/A
**Antibodies**		
Anti-detyrosinated (∆Y) α-tubulin, rabbit monoclonal, clone RM444	RevMAb Biosciences	Cat# 31-1335-00
Anti-∆C2-α-tubulin, rabbit monoclonal, clone RM447	RevMAb Biosciences	Cat# 31-1339-00
anti-PA-tag, clone NZ-1	Fujifilm Wako Pure Chemical	Cat# 016-25861
Anti-α-tubulin conjugated with Alexa Fluor 647, mouse monoclonal, clone DM1α	MilliporeSigma	Cat# 05-829-AF647
Anti-α-tubulin conjugated with FITC, mouse monoclonal, clone DM1α	MilliporeSigma	Cat# F2168
Anti-Halo tag, rabbit Polyclonal	Promega	Cat# G9281
Anti-NeonGreen tag, mouse monoclonal	Chromotek	Cat# 32f6
Anti-rabbit Alexa Fluor 680, goat polyclonal	Jackson ImmunoResearch	Cat# 711-625-152
Anti-mouse Alexa Fluor 680, donkey polyclonal	Jackson ImmunoResearch	Cat# 711-625-150
Anti-tyrosinated α-tubulin, rat monoclonal, clone YL1/2	Hotta et al, 2026	N/A
Anti-α-tubulin, mouse monoclonal, clone DM1α	MilliporeSigma	Cat# 05-829
anti-β-tubulin mouse monoclonal, clone E7	DSHB	Cat# E7
Anti-GAPDH mouse monoclonal, clone G-9	Santa Cruz	Cat# sc-365062; RRID: AB_10847862
Anti-GST mouse monoclonal	Nacalai USA	Cat# 04435-26
Anti-rabbit Alexa Fluor 488, goat	ThermoFisher	Cat# A-11034; RRID:AB_2576217
Anti-rat Alexa Fluor 594, goat	ThermoFisher	Cat# A-11007; RRID: AB_10561522
Anti-rat Alexa Fluor 680, goat	ThermoFisher	Cat# A-21096; RRID:AB_141554
Anti-rabbit IRDye 800CW, goat	LI-COR	Cat# 926-32211; RRID:AB_621843
Anti-mouse Alexa Fluor 700, goat	ThermoFisher	Cat# A-21036; RRID:AB_2535707
Anti-mouse DyLight 800, goat	ThermoFisher	Cat# SA5-10176; RRID:AB_2556756
**Chemicals, enzymes, and other reagents**		
Dulbecco’s Modified Eagle Medium (DMEM) high glucose	Gibco ThermoFisher	Cat# 11965092
FBS tetracycline-negative	R&D Systems	Cat# S10350
GlutaMAX	Gibco ThermoFisher	Cat# 35050061
Penicillin/streptomycin	Gibco ThermoFisher	Cat# 15140163
Doxycycline hyclate	MilliporeSigma	Cat# 9891
Lipofectamine	Invitrogen ThermoFisher	Cat# 11668030
Dulbecco’s Modified Eagle Medium (DMEM)	Gibco ThermoFisher	Cat# 11960044
Fetal Clone III serum	HyClone	Cat# SH30109.03
Janelia Fluor X 554 (JFX554) Halo ligand	Janelia Farms	Cat# JFX554
Trans-IT LT1	Mirus	Cat# MIR2305
Opti-MEM	Gibco ThermoFisher	Cat# 31985062
Protease inhibitors	MilliporeSigma	Cat# P8340
Bovine serum albumin	MilliporeSigma	Cat# A9647
Serum-free sf900 II SFM medium	Gibco ThermoFisher	Cat# 10902096
Grace’s insect cell culture medium	Gibco ThermoFisher	Cat# 11595-030
Antibiotic-Antimycotic	Gibco ThermoFisher	Cat# 15240062
Cellfectin II	Gibco ThermoFisher	Cat# 10362100
Strep-Tactin XT 4Flow beads	IBA	Cat# 2-5030-002
Biotin	MilliporeSigma	Cat# B4501
HeLa tubulin	Thomas et al, 2025	N/A
Alex647 HeLa tubulin	Thomas et al, 2025	N/A
Alex568 HeLa tubulin	Thomas et al, 2025	N/A
Taxol	Cytoskeleton	Cat# TXD01
DMSO	Invitrogen	Cat# D12345
Casein	MilliporeSigma	Cat# C8654
Glucose	MilliporeSigma	Cat# G7528
Glucose oxidase	MilliporeSigma	Cat# G7141-10KU
Catalase	MilliporeSigma	Cat# C3515
GTP	MilliporeSigma	Cat# G8877
GMPCPP	Jena Bioscience	Cat# NU405S
Glycerol	ThermoFisher	Cat# BP229-4
ATP	MilliporeSigma	A7699
ADP	MilliporeSigma	A2754
Hexokinase	MilliporeSigma	H5000
Peloruside A	Dan Sackett (NIH)	N/A
**Software**		
AIVIA	DRVision	https://www.aivia-software.com
Fiji/ImageJ	Schindelin et al, 2012	https://imagej.net/software/fiji
Prism v10.4.1 (627)	GraphPad Software	https://www.graphpad.com
**Other**		
Universal Mycoplasma Detection Kit	ATCC	Cat# 30-1012 K
HiPure Plasmid DNA miniprep kit	ThermoFisher	Cat# K210002
10 kDa MWCO spin column	Cytiva	Cat# GE28-9322-25
Fluorescent Protein Labeling Kit	ThermoFisher	Cat# A10235
Fab Preparation Kit	ThermoFisher	Cat# 44985
#1.5 coverslip	ThermoFisher	Cat# 2850-18
Glass slide	ThermoFisher	Cat# 12-544-3
Glass-bottom dishes	MatTek	Cat# P35GC-1.5-14-C

### Plasmids

For the Halo-MATCAP1 construct, a gene fragment containing Halo and human MATCAP1 (amino acids 1-471, UniProt Q68EN5) was synthesized and inserted into the pC1 vector using the NEBuilder HiFi DNA assembly kit. Additional MATCAP1 constructs were generated by replacing the Halo tag with monomeric NeonGreen (mNG-MATCAP1), tandem PA (GVAMPGAEDDVV), and TagRFP tags (PA-TagRFP-MATCAP1), or TwinStrep [WSHPQFEKGGGSGGGSGGSAWSHPQFEK (Schmidt et al, 2013)] and Halo tags (TwinStrep-Halo-MATCAP1) via PCR and Gibson assembly (NEB HiFi kit). The E281Q mNG-MATCAP1 mutant was generated by site-directed mutagenesis. To generate a construct for stable knock-in of Halo-MATCAP1 in HeLa Kyoto cells, the Halo-MATCAP1 fragment was PCR amplified and inserted into pEM791 vector that had been digested with BsrGI and AgeI. The resulting pEM791-Halo-MATCAP1 vector was used to establish knock-in HeLa cell lines expressing Halo-MATCAP1 in a doxycycline-inducible manner using recombination-mediated cassette exchange (Khandelia et al, 2011). A truncated, constitutively active rat kinesin-1 [KIF5C(1-560)] was amplified by PCR and subcloned into the pFastBac1 vector to generate KIF5C(1-560)]-Halo-TwinStrep tag for protein expression and purification. All plasmids were verified by DNA sequencing.

### Cell culture, transfection, lysate preparation, and immunofluorescence

HeLa Kyoto cells (RRID: CVCL_1922), Δ*TTL*Δ*CCP1* HeLa cells (Hotta et al, 2023), stable Halo-MATCAP1 HeLa cells, and wild-type and Δ*VASH1*Δ*VASH2* CHL-1 cells [kind gift from Dr. Thijn Brummelkamp (Nieuwenhuis et al, 2017)] were cultured in Gibco DMEM high glucose with 10% tetracycline-negative FBS, 1% GlutaMAX, and 1% penicillin/streptomycin and grown at 37 °C with 5% CO_2_. COS-7 [male *Ceropithecus aethiops* (green monkey) kidney fibroblast, RRID: CVCL_0224] cells were grown in Dulbecco’s modified Eagle medium supplemented with 10% (vol/vol) Fetal Clone III and 2 mM GlutaMAX and grown at 37 °C with 5% (vol/vol) CO_2_. All cell lines were screened routinely and found to be negative for mycoplasma contamination.

HeLa cells seeded on coverslips in a 6-well plate were transfected using Lipofectamine 2000 and used the following day. For the Halo-MATCAP1 stable HeLa cell line, Halo-MATCAP1 expression was induced by adding 2 µg/mL doxycycline to the culture medium along with Halo554 ligand (to label the protein) 16 h prior to fixation. Cells were fixed with ice-cold methanol for 10 min at −20 °C. Blocking was performed with 2% BSA in TBS (0.2 M Tris Base, 137 mM NaCl, pH 7.6) supplemented with 0.1% Triton X-100. Antibodies used were: anti-∆Y-α-tubulin antibody clone RM444 (final concentration 1 mg/ml, 1 h), anti-∆C2-α-tubulin antibody clone RM447 (concentration 1 mg/ml, 1 h), anti-PA-tag antibody clone NZ-1 (1:250, 45 min), DM1a-AlexaFluor647 (for PA-TagRFP-MATCAP1 experiment) or FITC-conjugated DM1a (for Halo-MATCAP1 experiment, 1:500). All antibodies were diluted in the blocking solution. DNA was stained with 5 mg/mL Hoechst. Coverslips were mounted with ProLong Diamond. Images were obtained with a DeltaVision microscope equipped with an Olympus Plan Apo N ×60/1.42 oil immersion lens. Images were deconvolved, and single optical sections are presented.

COS-7 cells were transfected using Trans-IT LT1 and Opti-MEM according to the manufacturer’s instructions. Halo-tagged protein was fluorescently labeled by adding 50 nM JFX554 Halo ligand to the cell medium after the transfection. Lysates from untransfected COS-7 cells without or with the addition of 50 nM JFX554 Halo ligand were prepared as controls. The cells were harvested 16 h post-transfection by low-speed centrifugation at 1500 × *g* for 5 min at 4 °C. The pellet was rinsed once in PBS and resuspended in ice-cold BRB80 buffer (80 mM Pipes/KOH pH 6.8, 1 mM MgCl_2_, and 1 mM EGTA) freshly supplemented with 1 mM phenylmethylsulfonylfluoride (PMSF) and protease inhibitors. After lysing the cells on ice using a Sonic Dismembrator with a microtip (Fisher Scientific), the lysate was clarified by centrifugation at 20,000 × *g* for 10 min at 4 °C. The supernatant was aliquoted, snap-frozen in liquid nitrogen, and stored at 80 °C until further use.

The concentration of Halo-MATCAP1 and mNG-MATCAP1 in the cell lysates was measured by a dot-blot, in which 1 µL of COS-7 lysates expressing MATCAP1 and a series of diluted known concentrations of protein fused with Halo or mNG tags were spotted onto a nitrocellulose membrane. The membrane was air-dried for 30 min to 1 h and immunoblotted with a primary antibody to Halo tag or mNeonGreen tag at room temperature for 1 h and subsequently with a secondary antibody 680 nm anti-rabbit or 680 nm anti-mouse at room temperature for 30 min. The fluorescence intensity of the spots on the nitrocellulose membrane was detected by Azure 600 and quantified based on the standard curve of known concentrations of protein using Fiji/ImageJ.

### Western blot

HeLa or CHL-1 (WT and VASH1/2 DKO) cells were lysed in NP-40 buffer (6 mM Na_2_HPO_4_, 4 mM NaH_2_PO_4_, 2 mM EDTA, 150 mM NaCl, 1% NP40 and protease inhibitors) and sonicated. Lysates were clarified by centrifugation at 21,000 × *g* for 15 min at 4 °C. Protein concentrations were determined using the Bradford protein assay with BSA as a standard. For SDS-PAGE, 15 µg of total protein per sample was loaded. Standard 10% acrylamide Laemmli gels were used, except for experiments presented in Fig. EV1, where high pH separation gels were used to resolve α- and β-tubulin as previously described (Banerjee et al, 2010). Proteins were transferred to nitrocellulose membranes, which were then blocked with 5% skim milk in PBS supplemented with 0.05% tween-20 (PBST) for 1 h at room temperature. Membranes were incubated with primary antibodies at 4 °C overnight. Following three washes with PBST, membranes were incubated with fluorescently labeled secondary antibodies for 1 h at room temperature, followed by another three washes. Fluorescent signals were detected using an Azure 600 imaging system (Azure Biosystems). Antibodies used: anti-tyrosinated α-tubulin rat monoclonal clone YL1/2 (1:3000); anti-∆Y α-tubulin rabbit monoclonal RM444 (0.1 mg/ml); anti-∆C2 α-tubulin rabbit monoclonal RM447 (0.5 mg/ml); anti-α-tubulin mouse monoclonal DM1a (1:3000), anti-β-tubulin mouse monoclonal E7 (1:1000); anti-PA-tag rat monoclonal NZ-1 (1:5000); anti-GAPDH mouse monoclonal G-9 (1:2000).

### Protein purification

For KIF5C(1-560)-Halo-TwinStrep protein expression and purification, Sf9 cells were cultured in suspension with serum-free sf900 II SFM medium supplemented with Antibiotic-Antimycotic in flasks at 28 °C in a non-CO_2_ non-humidified incubator with an orbital shaker platform set at 110 rpm. The cells were infected with baculovirus generated according to the Bac-to-Bac system (ThermoFisher). In brief, pFastBac1-KIF5C(1-560)-Halo-TwinStrep plasmid was transformed into DH10Bac *Escherichia coli* to generate recombinant bacmids. Bacmid DNA was isolated with the HiPure Plasmid DNA miniprep kit and confirmed by PCR analysis. Recombinant bacmid DNA was transfected into Sf9 cells using Cellfectin II. 7 d after transfection, the supernatant containing P1 baculovirus was collected by centrifugation at 3000 rpm for 3 min at 4 °C. The baculovirus was amplified by successive infection of Sf9 cells to generate P2 and P3 baculoviruses. Baculovirus-containing supernatants were stored at 4 °C in the dark. To purify protein, Sf9 cells were infected with 3% P3 baculovirus (vol/vol). 3 d after infection, the cells were harvested by centrifugation for 15 min at 3000 rpm at 4 °C. The pellet was washed once with PBS and resuspended in ice-cold lysis buffer (200 mM NaCl, 4 mM MgCl_2_, 0.5 mM EDTA, 1 mM EGTA, 0.5% igepal, 7% sucrose, and 20 mM imidazole-HCl, pH 7.5) supplemented with 2 mM ATP, 1 mM PMSF, 5 mM DTT, and protease inhibitor cocktail. After 30 min incubation on ice, the lysates were clarified by ultracentrifugation for 20 min at 20,000 rpm in F12-8x50y rotor (Sorvall 3421), and the supernatants were incubated with Strep-Tactin beads for 1 h at 4 °C with rotation. Strep-Tactin beads with bound proteins were collected in a PD-10 column and washed with wash buffer (150 mM KCl, 25 mM imidazole-HCl, pH 7.5, 5 mM MgCl_2_, 1 mM EDTA, and 1 mM EGTA) supplemented with 1 mM PMSF, 3 mM DTT, 3 mM ATP, and protease inhibitor cocktail. Bound proteins were eluted with elution buffer (25 mM KCl, 25 mM imidazole-HCl, pH 7.5, 5 mM EGTA, 2 mM MgCl_2_, 2 mM DTT, 0.1 mM ATP, 1 mM PMSF, protease inhibitor cocktail, and 10% glycerol) supplemented with 50 mM biotin in 6×0.5 mL fractions. The eluted fractions were analyzed by SDS-PAGE, and fractions containing the peak protein were selected by visual inspection of the gel. Protein fractions were combined and dialyzed against dialysis buffer (25 mM imidazole-HCl, pH 7.5, 25 mM KCl, 5 mM EGTA, 2 mM MgCl_2_, 2 mM DTT, 0.1 mM ATP and 10% glycerol). After 2 h, the buffer was changed to fresh dialysis buffer and dialyzed overnight at 4 °C to remove biotin from the sample. The protein sample was collected by centrifugation, and aliquots were snap-frozen in liquid nitrogen and stored in −80 °C until further use.

For purification of TwinStep-Halo-MATCAP1 protein COS-7 cells were transfected with a plasmid for expression of TwinStrep-Halo-MATCAP1 protein and were harvested by centrifugation 16-20 h later. The cells from four 10 cm dishes were lysed in 1 mL of lysis buffer (25 mM HEPES, 115 mM KOAc, 5 mM NaOAc, 5 mM MgCl_2_, 0.5 mM EGTA, 10% TX-100, pH 7.4) supplemented with 1 mM PMSF and protease inhibitor cocktail and cleared by centrifugation. The cell lysates were incubated with 100 µl of Strep-Tactin XT 4Flow beads for 1 h at 4 °C. Beads were washed three times with washing buffer (20 mM HEPES/ KOH pH 7.5, 150 mM NaCl, 1 mM DTT). The proteins were eluted in 1 mL of washing buffer containing 50 mM biotin (Sigma) for 1 h at 4 °C. The eluted protein was further concentrated to 100 µL using a 10 K MWCO spin column. Purified proteins were snap-frozen in liquid nitrogen and stored at −80 °C.

### Preparation of ΔY-Fab^488^ and ΔC2-Fab^647^ probes

Fluorescently-labeled ∆Y-Fab^488^ and ∆C2-Fab^647^ probes were generated from recombinant rabbit monoclonal antibodies against ∆Y-and ∆C2-α-tubulin. Fluorescent labeling of the parental IgG and subsequent Fab preparation were prepared using the Fluorescent Protein Labeling Kit and the Fab Preparation Kit according to the manufacturer’s instructions.

### TIRF microscopy

All in vitro assays (below) were performed on an inverted Nikon Ti-E/B total internal reflection fluorescence (TIRF) microscope with a perfect focus system, a 100 × 1.49 NA oil immersion TIRF objective, three 20 mW diode lasers (488, 561, and 640 nm) and EMCCD camera (iXon^+^ DU879; Andor). Image acquisition was controlled using Nikon Elements software. A flow chamber (~10 μl volume) was assembled by attaching a clean #1.5 coverslip (ThermoFisher) to a glass slide (ThermoFisher) with two strips of double-sided tape. All in vitro assays were performed in the flow chambers at room temperature.

### Microtubule polymerization

All experiments utilized tubulin purified from HeLa S3 suspension cells which lack pre-existing ΔY and ΔC2 PTMs (Thomas et al, 2025). The tubulin was purified using TOG affinity chromatography and labeled with fluorescent dyes as described (Thomas et al, 2025). For fluorescently labeled microtubules, the tubulin mix contained 10% Alexa647-labeled or Alexa568-labeled HeLa tubulin. All microtubules were stored in the dark at 37 °C until further use.

#### Taxol-stabilized GDP-microtubules

Microtubules were polymerized from 30 μM HeLa tubulin in BRB80 buffer supplemented with 2.5 mM GTP and 4 mM MgCl_2_ at 37 °C for 35 min. A 5× volume of prewarmed BRB80 buffer containing 10 μM Taxol was added. The microtubules were centrifuged at 15,000 rpm for 10 min at room temperature. The pellet was resuspended in the same 5× volume of prewarmed BRB80 buffer containing 10 μM Taxol.

#### Taxol-stabilized ∆Y-microtubules

Taxol-stabilized HeLa microtubules were incubated with cell lysate containing 0.07–1 nM unlabeled VASH1/SVBP and 10 μM Taxol in P12 buffer (12 mM Pipes/KOH pH 6.8, 1 mM MgCl_2_, 1 mM EGTA) for 2 s–13 min to generate microtubules with different levels of detyrosination. The enzyme was removed from the microtubules either by a high-salt wash (BRB80 buffer supplemented with 200 mM NaCl) or by incubation with BRB80 buffer supplemented with 100 μM EpoY for 1 min, followed by two washes with the corresponding blocking buffer used in the assays.

#### Taxol-stabilized ∆C2-microtubules

Taxol-stabilized HeLa microtubules were incubated with cell lysate containing 0.7–2 nM MATCAP1 for 3 min–1 h to generate microtubules with different levels of ΔC2 modification. The enzyme was removed from the microtubules by washing with high-salt buffer (BRB80 buffer supplemented with 200 mM NaCl).

#### PelA-stabilized GDP-microtubules

Microtubules were polymerized from 30 μM HeLa tubulin in BRB80 buffer supplemented with 2.5 mM GTP and 4 mM MgCl_2_ at 37 °C for 35 min. A 2× volume of prewarmed BRB80 buffer containing 1 μM PelA was added, and the microtubules were centrifuged at 15,000 rpm for 10 min at room temperature. The pellet was resuspended in 3× volume of prewarmed BRB80 buffer containing 1 μM PelA.

#### GMPCPP-microtubules

Microtubules were polymerized from 5 μM HeLa tubulin in BRB80 buffer supplemented with 4 mM GMPCPP and 1 mM MgCl_2_ at 37 °C for 35 min. The microtubules were then centrifuged at 15,000 rpm for 10 min at room temperature. The pellet was resuspended in the same volume of prewarmed BRB80 buffer containing 1 mM DTT.

#### GDP-microtubules

Microtubules were polymerized from 60 µM HeLa tubulin in BRB80 buffer supplemented with 2.5 mM GTP and 5 mM MgCl_2_ at 37 °C for 35 min. A 5× volume of prewarmed BRB80 buffer containing 25% glycerol and 1 mM GTP was added. The microtubules were centrifuged at 15,000 rpm for 10 min at room temperature. The pellet was resuspended in the same 5× volume of BRB80 buffer containing 25% glycerol and 1 mM GTP.

### Microscopy-based enzymatic assays

To examine the biogenesis of ∆Y- and/or ∆C2-microtubules along Taxol-stabilized HeLa microtubules, polymerized microtubules were introduced into a flow chamber and incubated for 3 min at room temperature to allow for nonspecific adsorption to the coverslip. After washing with blocking buffer (1 mg/mL casein in P12 buffer), the flow chamber was infused with imaging buffer [P12 buffer supplemented with 3 mg/mL casein, 10 µM Taxol, oxygen scavenger mix (1 mM DTT, 1 mM MgCl_2_, 10 mM glucose, 0.2 mg/ml glucose oxidase, and 0.08 mg/ml catalase)] containing VASH1/SVBP-Halo or Halo-MATCAP1 (in cell lysate or as purified protein), and 592 pg/μL ΔY-Fab^488^ and/or 5 ng/µL ∆C2-Fab^647^ probe. An equivalent volume of untransfected COS-7 cell lysate was used as a negative control. Images were acquired every 5 s for 10 min or every 30 s for 60 min by TIRF microscopy. For quantification, the mean fluorescence intensity of the ΔY-Fab^488^ and/or ∆C2-Fab^647^ probes along the microtubule in each frame was measured by drawing a line along each microtubule (width = 3 pixels) using Fiji/ImageJ. To calculate the change in fluorescence intensity, the mean fluorescence intensity of the Fab probe measured in the first frame was subtracted from the mean fluorescence intensity in each subsequent frame.

To examine the biogenesis of ∆Y-microtubules along HeLa microtubules in different nucleotide or conformational states, GDP-microtubules or GMPCPP-microtubules were introduced into a flow chamber and incubated for 3 min at room temperature to allow for nonspecific adsorption to the coverslip. After washing with blocking buffer (1 mg/mL casein and 10% glycerol in BRB80 buffer), the flow chamber was infused with binding buffer (BRB80 buffer supplemented with 10% glycerol, 0.3 mg/mL casein, Halo-MATCAP1 in cell lysate or purified protein, and 20 µM Taxol or DMSO) and incubated for 6 min at 37 °C to allow MATCAP1 to detyrosinate microtubules. To remove MATCAP1 from the microtubules, the flow chamber was sequentially washed with (1) blocking buffer 2 (1 mg/mL casein and 25% glycerol in BRB80 buffer), (2) blocking buffer 2 supplemented with 200 mM NaCl, and (3) blocking buffer 3 (1 mg/mL casein and 25% glycerol in P12 buffer). Subsequently, the flow chamber was washed with imaging buffer (P12 buffer supplemented with 25% glycerol, 0.3 mg/mL casein, oxygen scavenger mix, and 592 pg/μL ΔY-Fab^488^ probe) and incubated for 5 min. The flow chamber was then sealed with molten paraffin wax and imaged. Snapshots were acquired using a TIRF microscope. The fluorescence intensities along the microtubules were measured using Fiji/ImageJ (width = 3 pixels), and the fluorescence intensity of an adjacent region was subtracted to account for background noise. Notably, different buffers and concentrations of glycerol were used at each step to optimize microtubule stability, microtubule binding behavior of MATCAP1, and ΔY-Fab^488^ probe. We found that 25% glycerol decreases the microtubule binding of MATCAP1, and the BRB80 buffer reduces the labeling of the ΔY-Fab^488^ probe for ΔY-microtubules.

### Microscopy-based microtubule binding assays

To examine the landing rate of MATCAP1 on Taxol-stabilized microtubules with different α-tubulin CTT modifications, modified microtubules were generated as described above. The microtubules were introduced into a flow chamber and incubated at room temperature for 3 min to allow nonspecific adsorption to the coverslip. After washing with blocking buffer (1 mg/ml casein in P12 buffer), the flow chamber was infused with imaging buffer [P12 buffer supplemented with 10 µM Taxol, 3 mg/ml casein, oxygen scavenger mix, and 10 pM Halo^554^-MATCAP1 in cell lysate]. An equivalent volume of untransfected COS-7 cell lysate containing Halo JFX554 ligand was used as a negative control. To examine the landing rate of Halo-MATCAP1 in cell lysate on GDP-microtubules versus GMPCPP-microtubules, 25% glycerol was added to all the buffers to stabilize the microtubules during the assay. The flow chamber was sealed with molten paraffin wax and imaged. Images were acquired continuously every 100 ms for 300 frames by TIRF microscopy. Maximum-intensity projections were generated, and kymographs were produced by drawing along microtubules (width = 3 pixels) using Fiji/ImageJ. The landing rate was calculated as the number of microtubule-bound molecules (including molecules pre-bound prior to imaging and molecules landing during the imaging period) per minute per nanomolar of protein per micrometer of microtubule.

To examine the microtubule binding of MATCAP1 to HeLa microtubules in different nucleotide or conformational states, GDP-microtubules or GMPCPP-microtubules were added to flow chambers and incubated for 3 min at room temperature to allow nonspecific adsorption to the coverslips. After washing with blocking buffer (1 mg/mL casein and 10% glycerol in BRB80 buffer), the flow chamber was infused with imaging buffer (BRB80 buffer supplemented with 10% glycerol, 0.3 mg/mL casein, oxygen scavenger mix, 1 nM Halo-MATCAP1 in cell lysate or 1.5 nM purified protein, and 20 µM Taxol or DMSO). The flow chamber was then sealed with molten paraffin wax and imaged. Snapshots were acquired using TIRF microscopy. The fluorescence intensities along the microtubules were measured using Fiji/ImageJ (width = 3 pixels), and the fluorescence intensity of an adjacent region was subtracted to account for background noise.

For microtubule binding experiments on Taxol-stabilized Y- and ∆Y- microtubules, the modified microtubules were generated as described above. The blocking buffer was 1 mg/ml casein in BRB80 buffer, and the imaging buffer was BRB80 buffer supplemented with 1 mg/ml casein, oxygen scavenger mix, and cell lysate containing 1 nM WT or E281Q mNG-MATCAP1.

For microtubule binding experiments on Taxol- and PelA-stabilized GDP-microtubules, the blocking buffer was 1 mg/ml casein in BRB80 buffer, and the imaging buffer was BRB80 buffer supplemented with 1 mg/ml casein, oxygen scavenger mix, and cell lysate containing 2 nM WT or E281Q mNG-MATCAP1.

For microtubule binding experiments during the stepping of mobile KIF5C(1-560)-Halo-TwinStrep protein, the blocking buffer was 1 mg/mL casein and 25% glycerol in P12 buffer, and the imaging buffer was P12 buffer supplemented with 25% glycerol, 0.3 mg/mL casein, oxygen scavenger mix, and 3 nM purified Halo-MATCAP1 protein. The assays were supplemented with purified KIF5C(1-560) protein under three conditions: (1) without KIF5C(1-560), (2) with 100 nM KIF5C(1-560) and 2 mM ATP, or (3) with 100 nM KIF5C(1-560), 2 mM ADP, and 2 unit/mL Hexokinase.

To examine the conditions that detach MATCAP1 from microtubules, GDP-microtubules were introduced into a flow chamber and incubated for 3 min at room temperature to allow nonspecific adsorption to the coverslip. After washing with blocking buffer 1 (1 mg/mL casein and 10% glycerol in BRB80 buffer), the flow chamber was infused with binding buffer (BRB80 buffer supplemented with 10% glycerol, 0.3 mg/mL casein) containing 1 nM Halo^554^-MATCAP1 in cell lysate and incubated for 3 min at 37 °C. To remove MATCAP1 from the microtubules, the flow chamber was sequentially washed with (1) blocking buffer 2 (1 mg/mL casein and 25% glycerol in BRB80 buffer), (2) blocking buffer 2 supplemented with either 26 µM GST-αCTT, 10 µM free tubulin, 2 mM ZnCl_2_, or indicated concentrations of NaCl, and (3) blocking buffer 2. Subsequently, the flow chamber was infused with imaging buffer (P12 buffer supplemented with 25% glycerol, 0.3 mg/mL casein, and oxygen scavenger mix). The flow chamber was then sealed with molten paraffin wax and imaged. Snapshots were acquired using a TIRF microscope. The ionic strength (IS) of each buffer was calculated based on the molar concentration of each ion and its counterion using the formula:

$${IS}=\frac{1\,}{2}\sum ({C}_{i}{Z}_{i}^{2})$$

where ${C}_{i}$ is the molar concentration of the ion i and ${Z}_{i}$ is its charge. The IS of 80 mM PIPES is 168 mM, the IS of 1 mM EGTA is 2 mM, the IS of 1 mM MgCl_2_ is 3 mM, and thus the IS of BRB80 buffer is 173 mM. The additional IS contributed by NaCl was included in the IS calculation for the BRB80 buffer containing the corresponding concentration of NaCl.

### Microtubule-gliding assays

The flow chamber was coated with 50 µg/mL anti-Halo antibody and then washed with blocking buffer (0.5 mg/mL casein, 10 µM Taxol in BRB80 buffer). 200 nM KIF5C(1-560)-Halo-TwinStrep purified protein in BRB80 buffer supplemented with 10 µM Taxol, 4 mM ATP, and 1 mg/mL casein was added in order to bind KIF5C proteins to the antibody-coated coverslip. After washing with blocking buffer, the flow chamber was infused with gliding buffer (BRB80 buffer supplemented with HiLy488-labeled Taxol-stabilized microtubules, 20 µM Taxol, 4 mM ATP, and oxygen scavenger mix). The flow chamber was then sealed with molten paraffin wax and imaged. Images were acquired continuously every 100 ms for 300 frames. Maximum-intensity projections were generated, and kymographs were produced by drawing along microtubules (width = 3 pixels) using Fiji/ImageJ. The velocity of microtubule gliding was calculated as the gliding distance divided by the gliding time.

## Supplementary information

Peer Review File

Source data Fig. 1

Source data Fig. 2

Source data Fig. 3

Source data Fig. 4

Source data Fig. 5

Source data Fig. 6

Expanded View Figures

## Data Availability

This study includes no data deposited in external repositories. The source data of this paper are collected in the following database record: biostudies:S-SCDT-10_1038-S44318-026-00772-6.
