# Supplementary figures and images for: MATCAP1 preferentially binds an expanded tubulin conformation to generate detyrosinated and ΔC2 α-tubulin
Source: EMBO J. 2026 Apr 13;45(12):4257–82. doi: 10.1038/s44318-026-00772-6 (PMC13269789; doi:10.1038/s44318-026-00772-6)

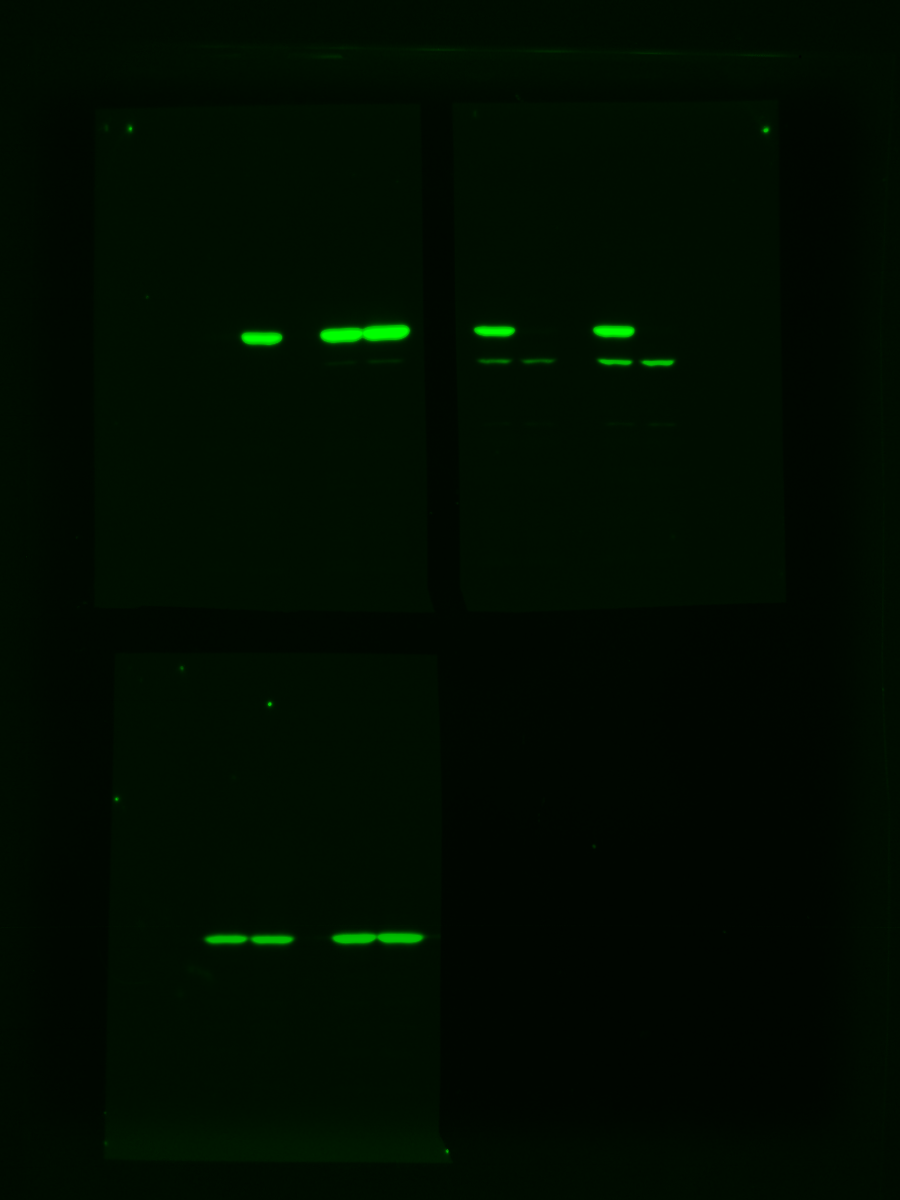

Supplement: Supplementary file 2 — Source data Fig. 1 [file 44318_2026_772_MOESM2_ESM.zip › Figure 1/1F/western_GAPDH_raw.tif]

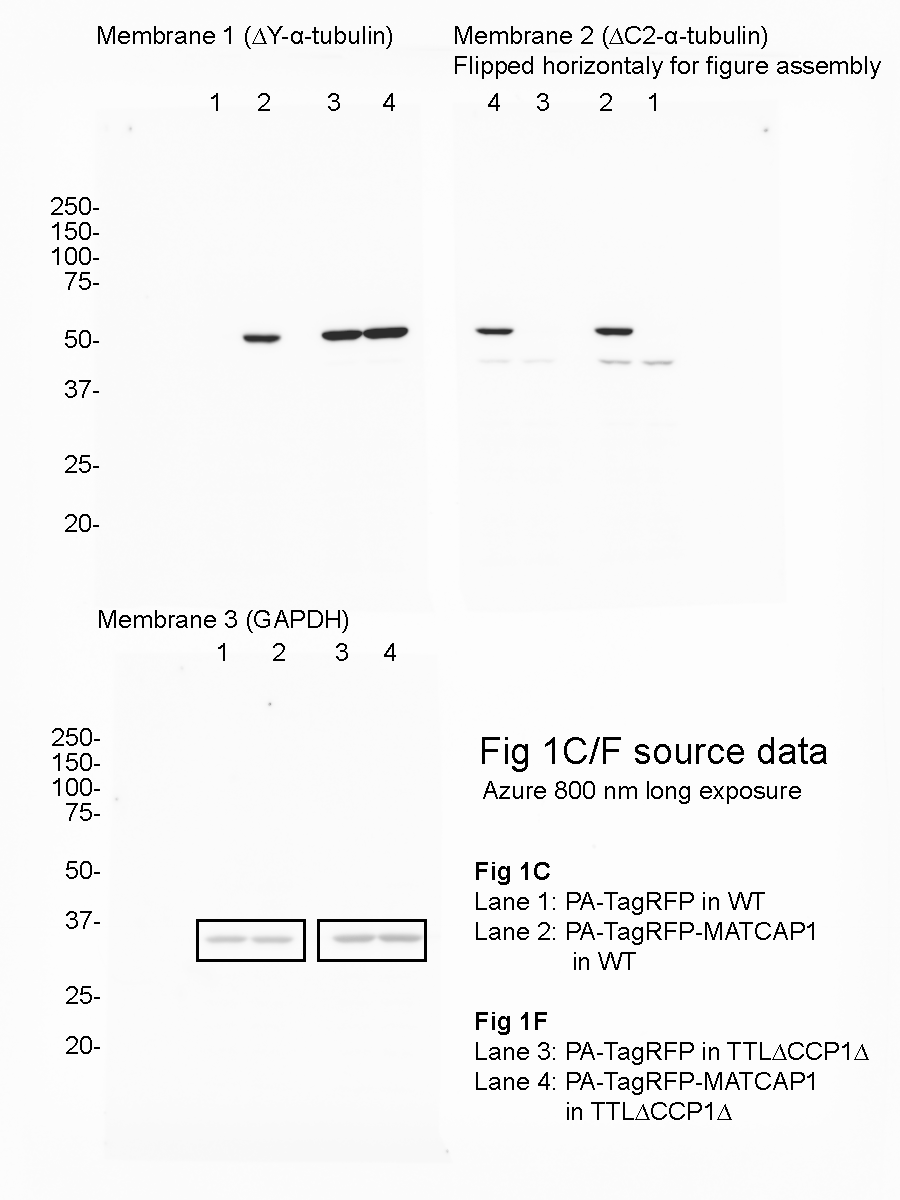

Supplement: Supplementary file 2 — Source data Fig. 1 [file 44318_2026_772_MOESM2_ESM.zip › Figure 1/1F/western_GAPDH_labeled.tif]

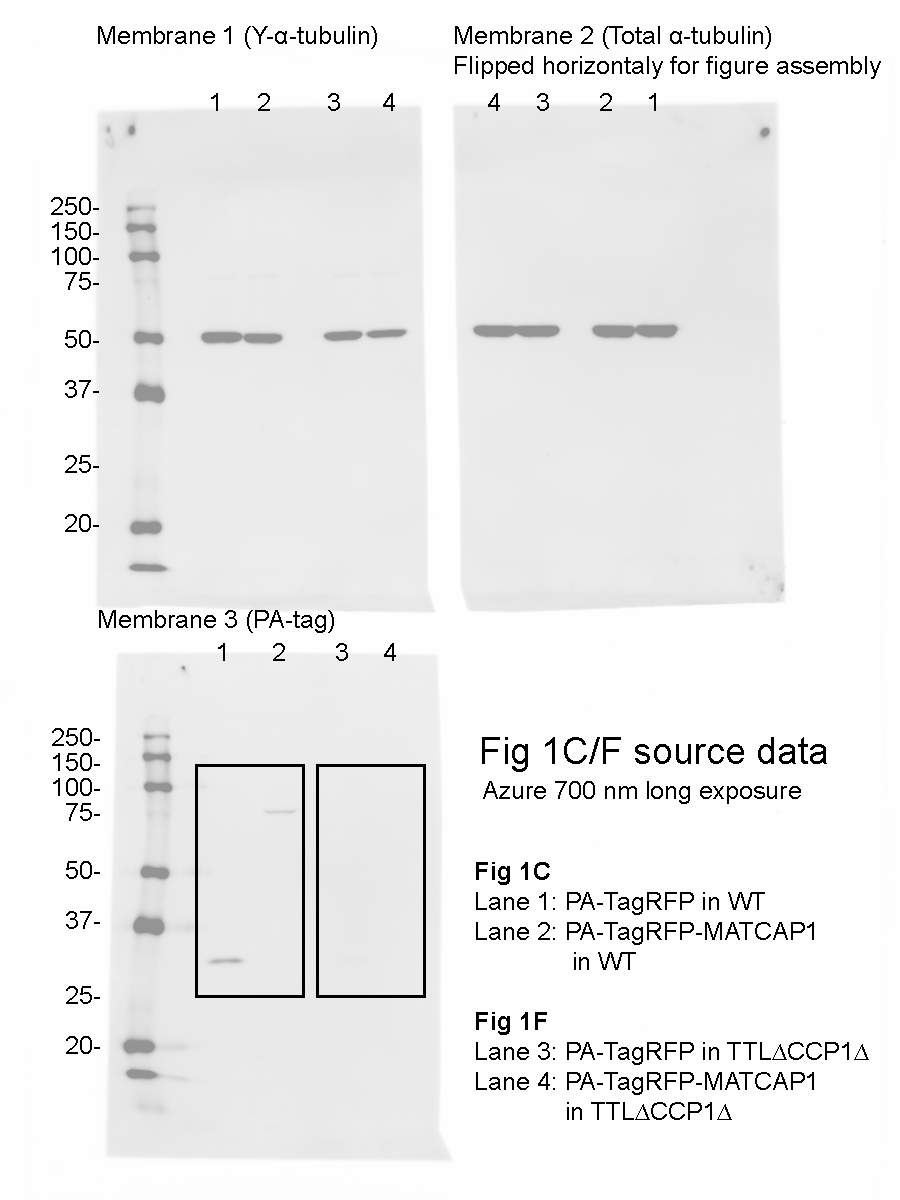

Supplement: Supplementary file 2 — Source data Fig. 1 [file 44318_2026_772_MOESM2_ESM.zip › Figure 1/1F/western_PA-tag_labeled.tif]

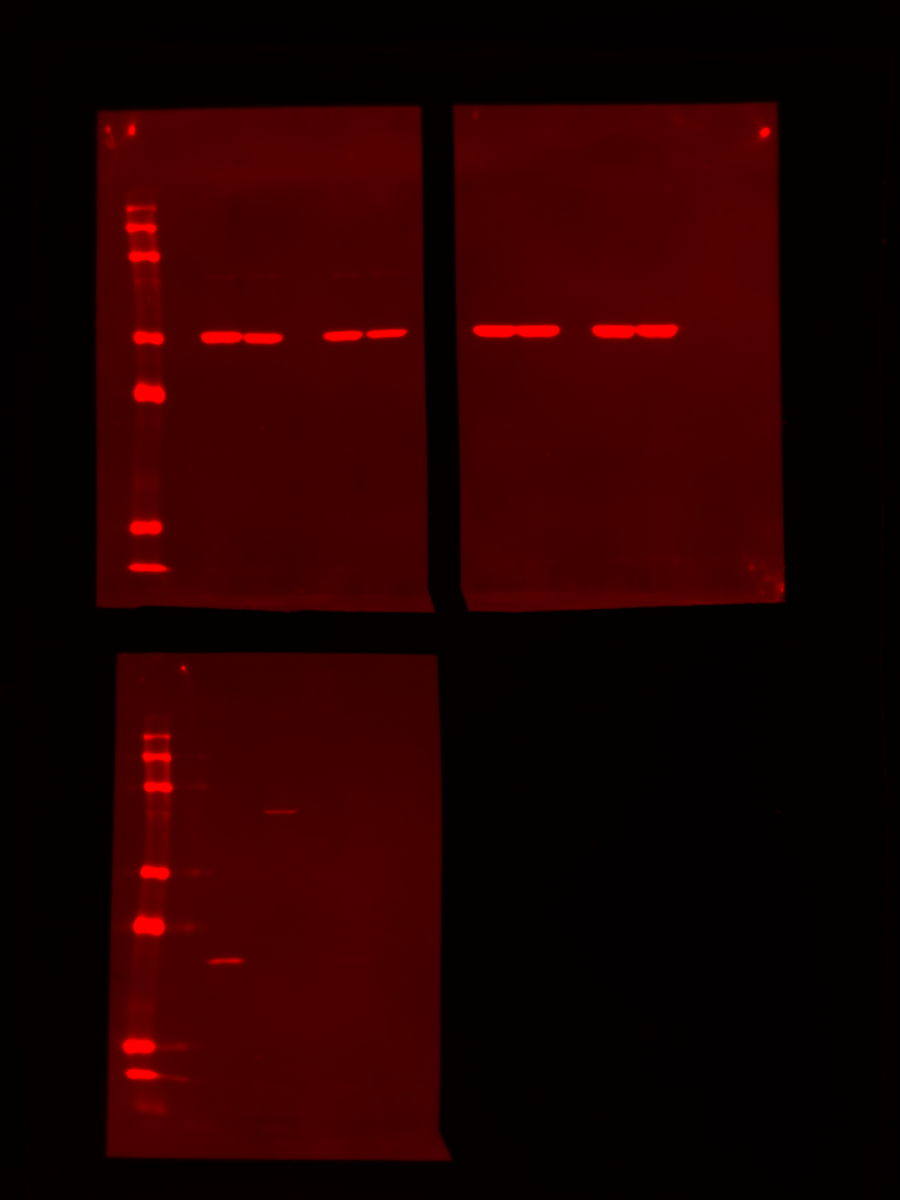

Supplement: Supplementary file 2 — Source data Fig. 1 [file 44318_2026_772_MOESM2_ESM.zip › Figure 1/1F/western_PA-tag_raw.tif]

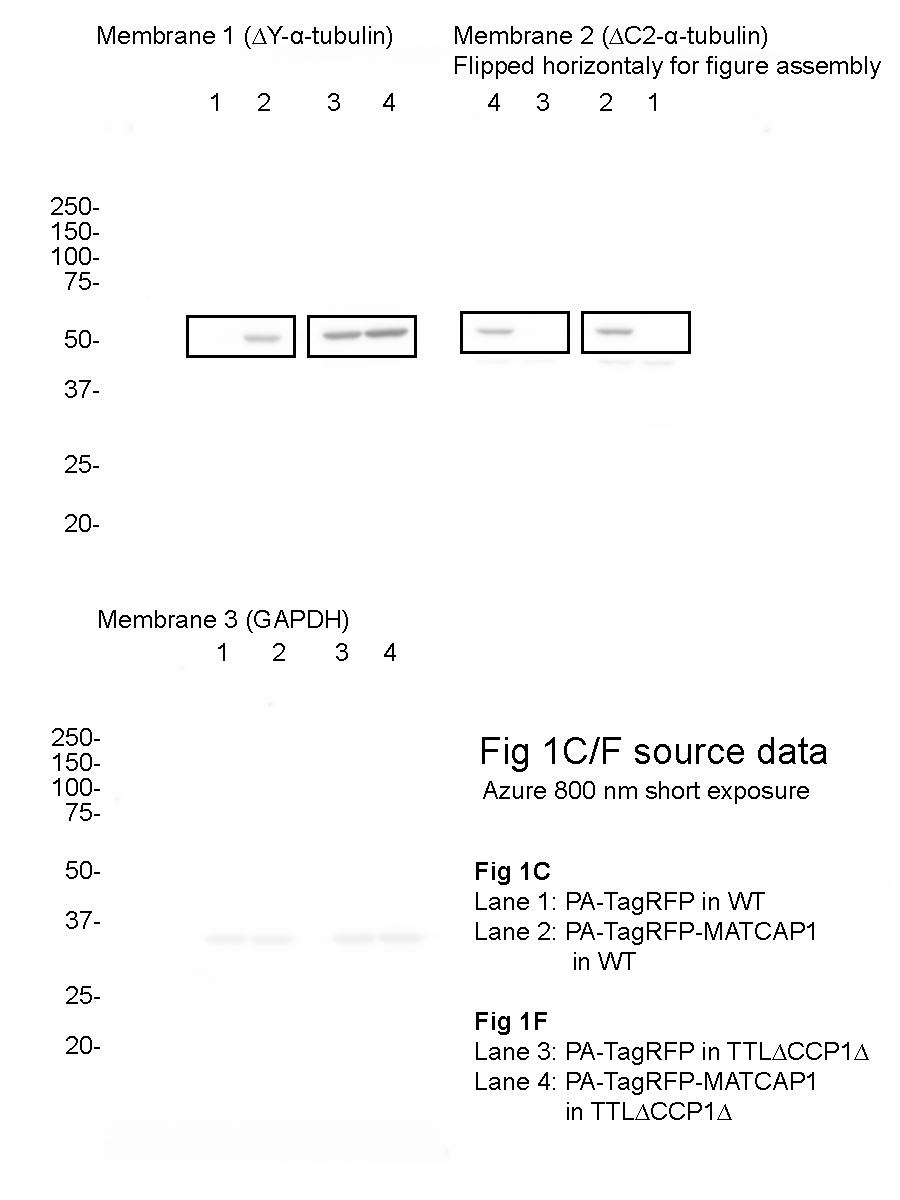

Supplement: Supplementary file 2 — Source data Fig. 1 [file 44318_2026_772_MOESM2_ESM.zip › Figure 1/1F/western_dY, dC2-a-tubulin_labeled.tif]

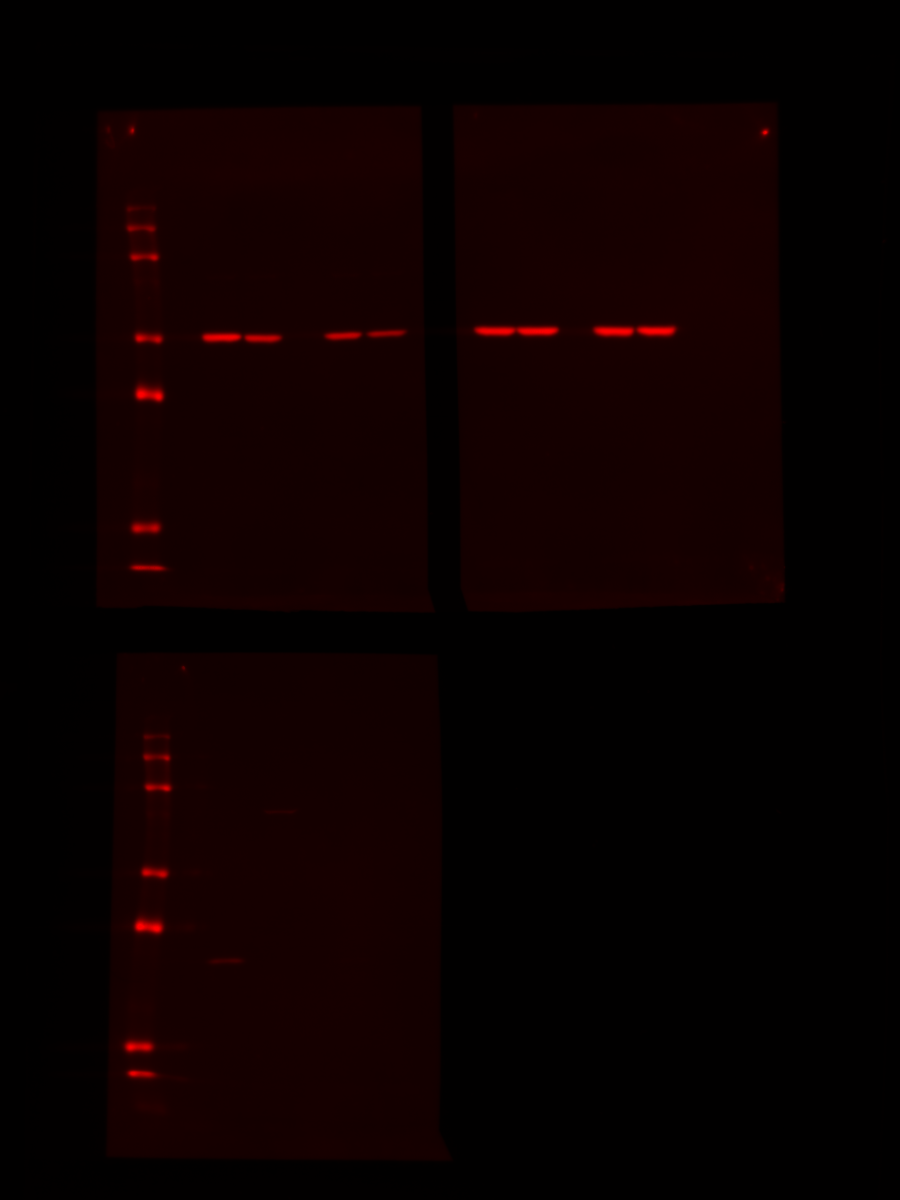

Supplement: Supplementary file 2 — Source data Fig. 1 [file 44318_2026_772_MOESM2_ESM.zip › Figure 1/1F/western_Y, total-a tubulin_raw.tif]

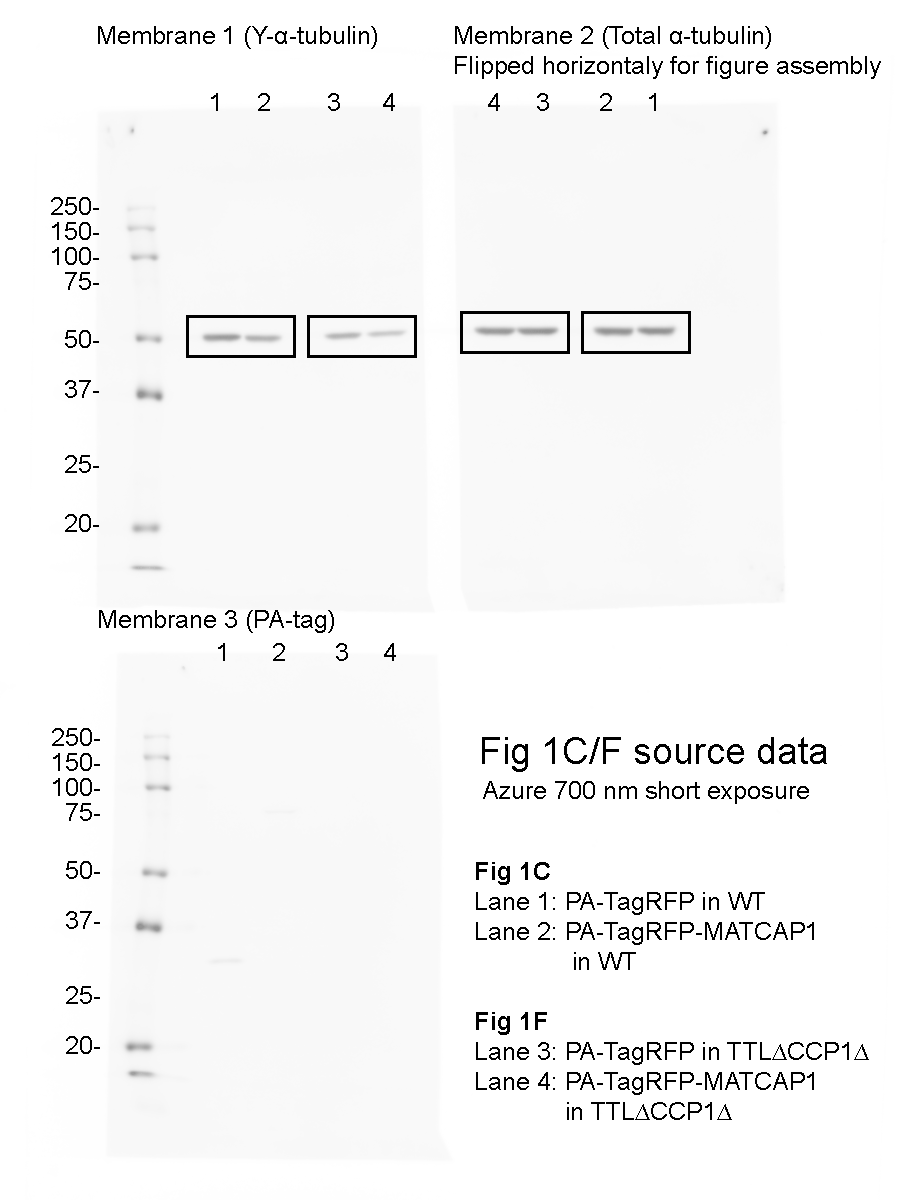

Supplement: Supplementary file 2 — Source data Fig. 1 [file 44318_2026_772_MOESM2_ESM.zip › Figure 1/1F/western_Y, total-a tubulin_labeled.tif]

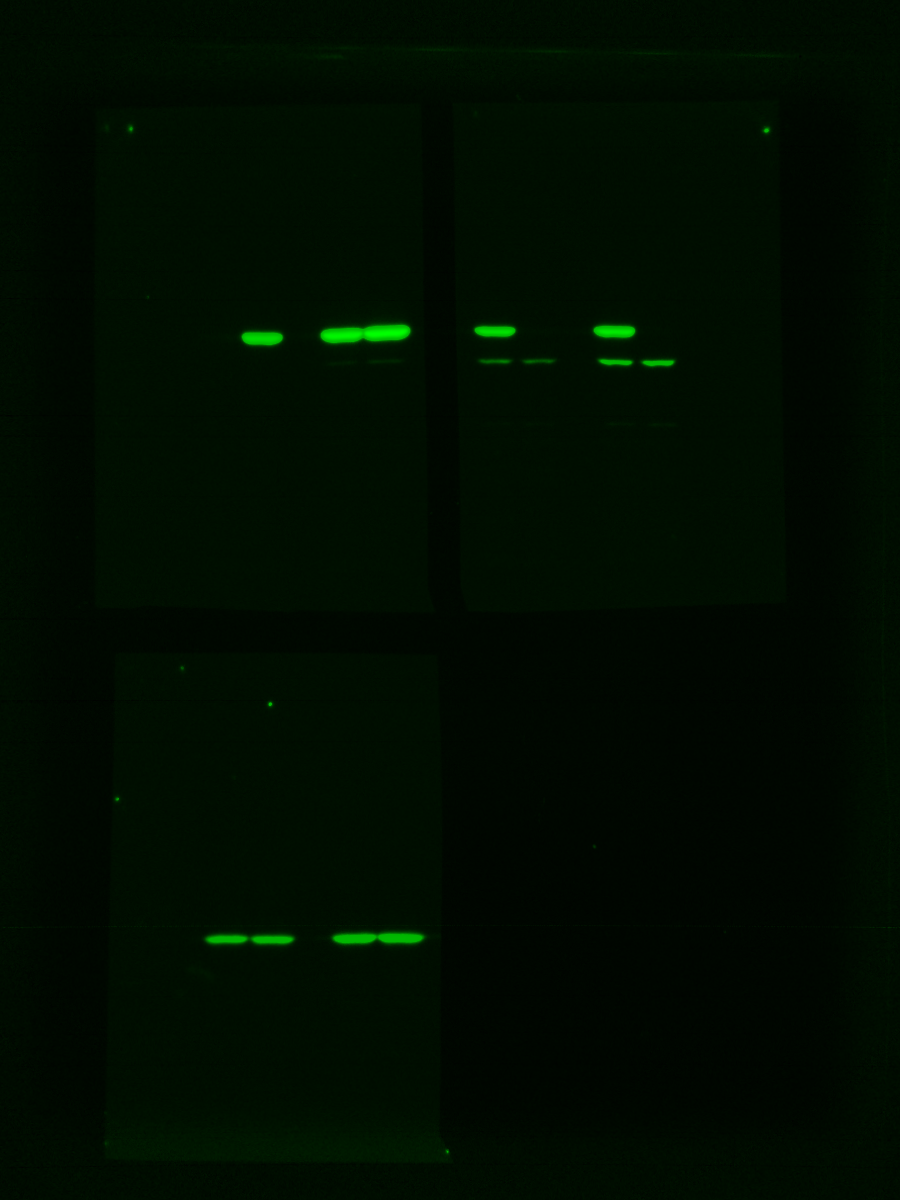

Supplement: Supplementary file 2 — Source data Fig. 1 [file 44318_2026_772_MOESM2_ESM.zip › Figure 1/1F/western_dY, dC2-a-tubulin_raw.tif]

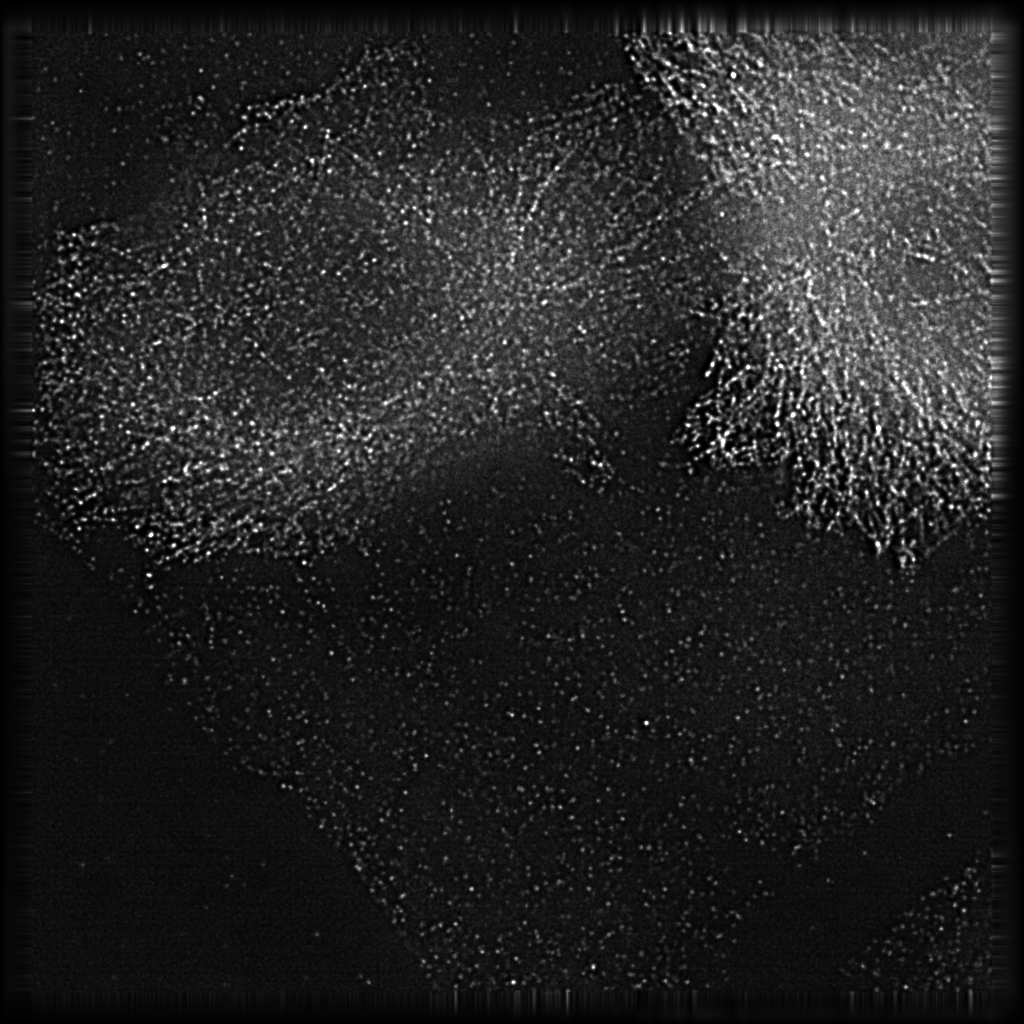

Supplement: Supplementary file 2 — Source data Fig. 1 [file 44318_2026_772_MOESM2_ESM.zip › Figure 1/1A/image_MATCAP1.tif]

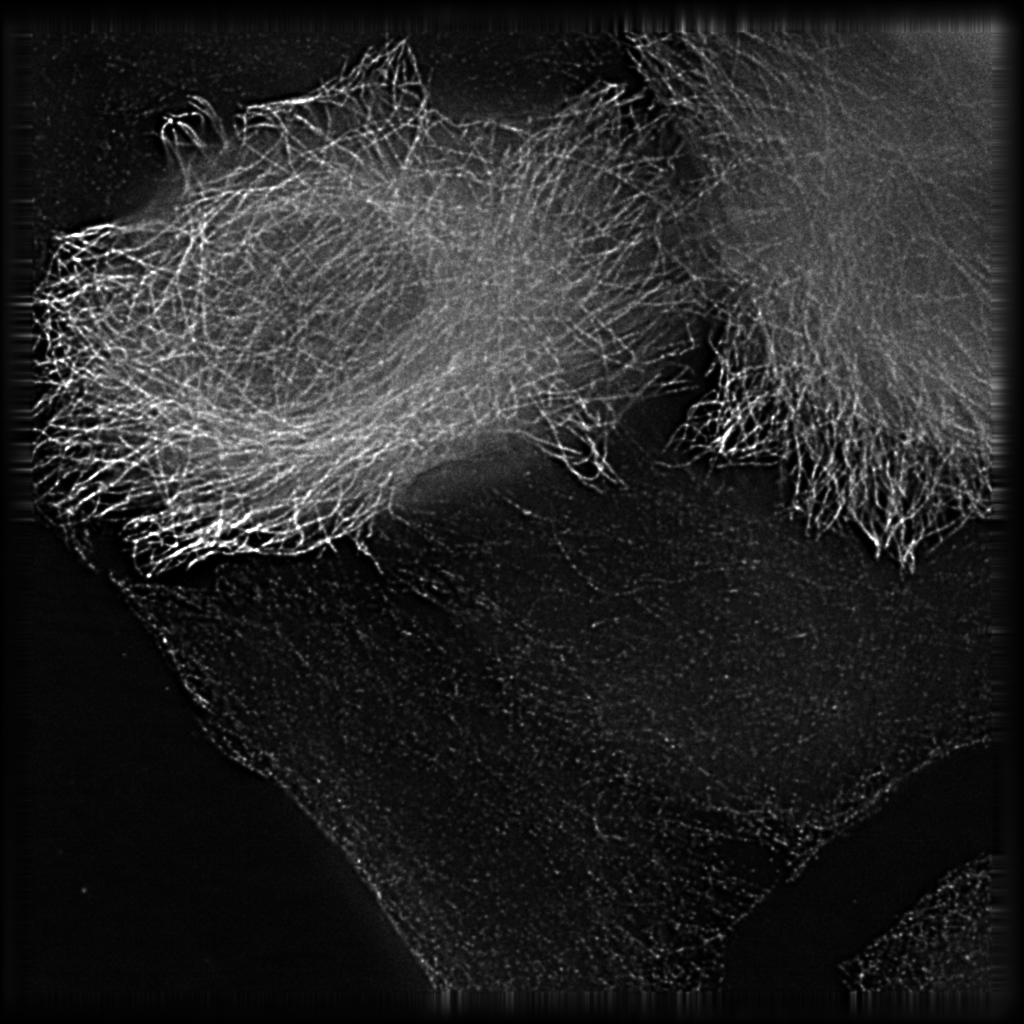

Supplement: Supplementary file 2 — Source data Fig. 1 [file 44318_2026_772_MOESM2_ESM.zip › Figure 1/1A/image_dY-a-tubulin.tif]

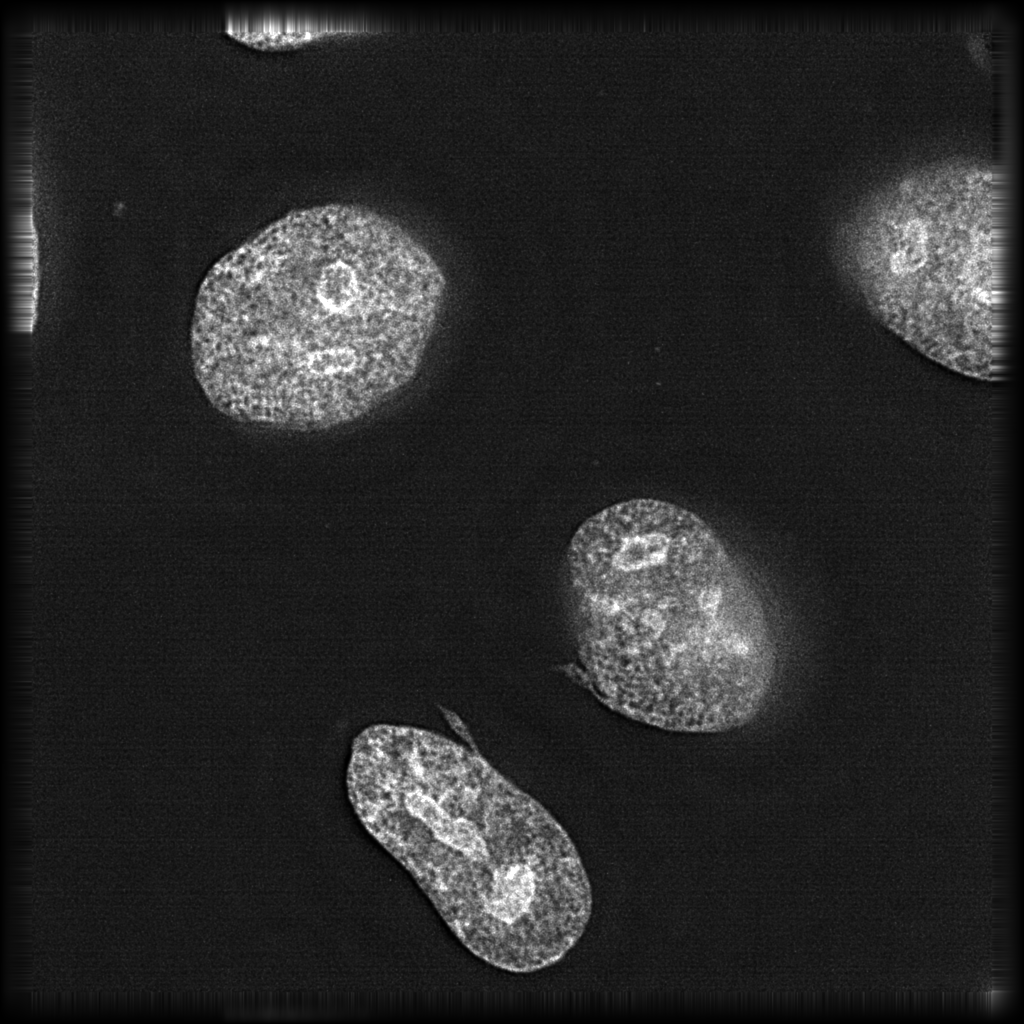

Supplement: Supplementary file 2 — Source data Fig. 1 [file 44318_2026_772_MOESM2_ESM.zip › Figure 1/1A/image_DNA.tif]

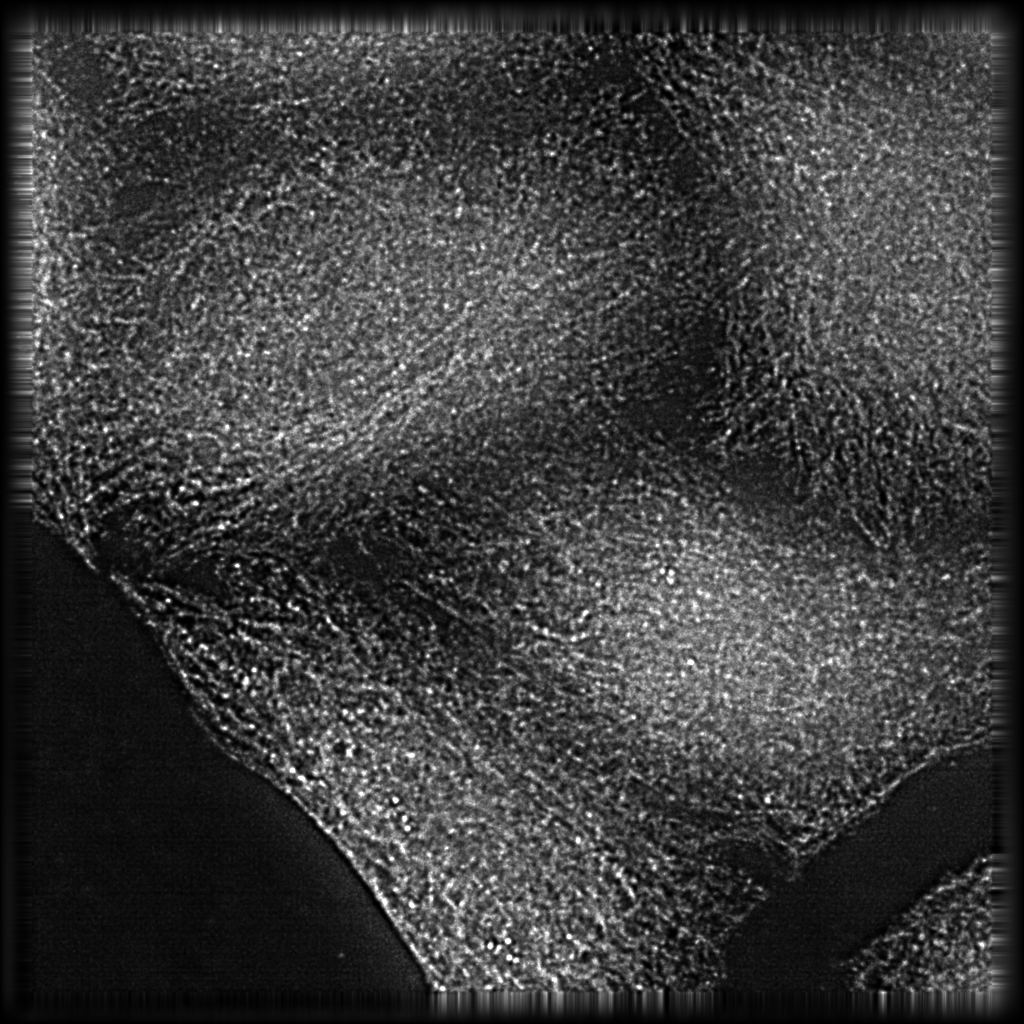

Supplement: Supplementary file 2 — Source data Fig. 1 [file 44318_2026_772_MOESM2_ESM.zip › Figure 1/1A/image_MTs.tif]

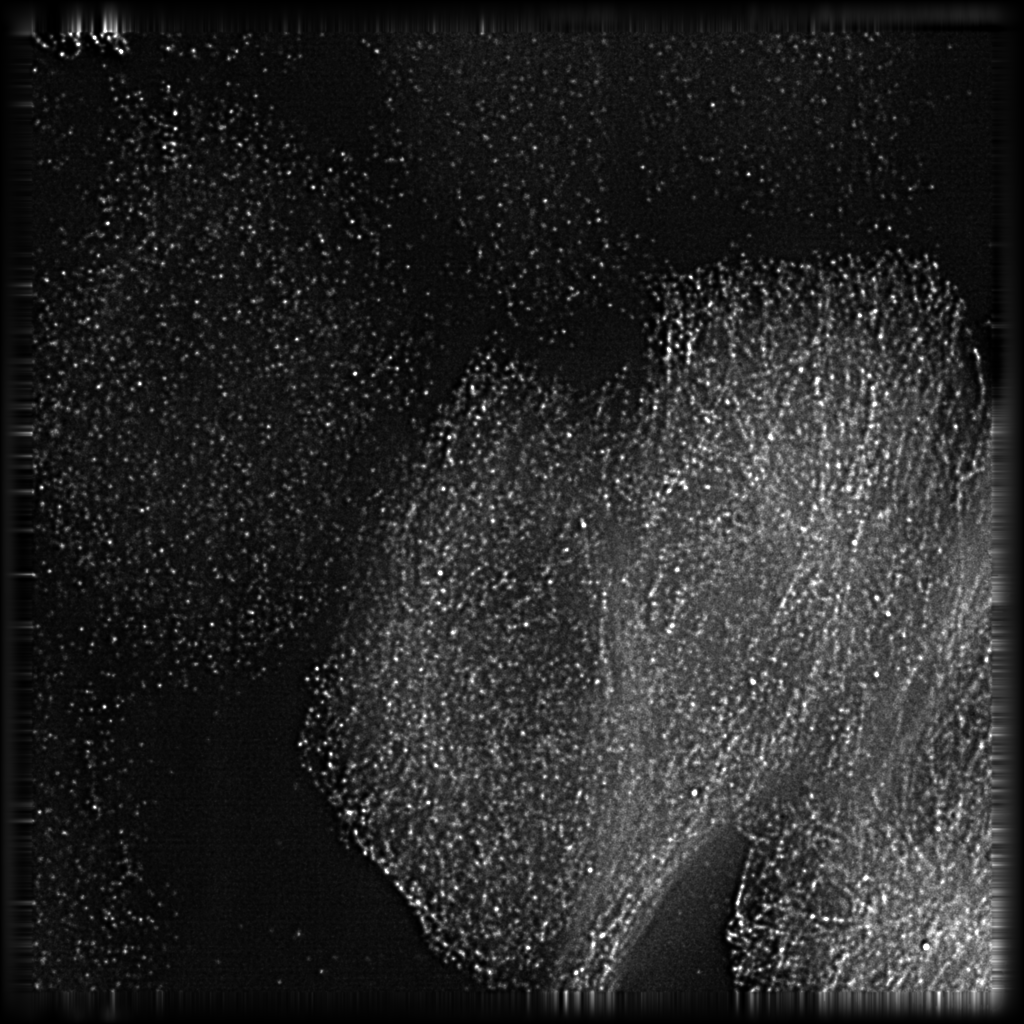

Supplement: Supplementary file 2 — Source data Fig. 1 [file 44318_2026_772_MOESM2_ESM.zip › Figure 1/1B/image_MATCAP1.tif]

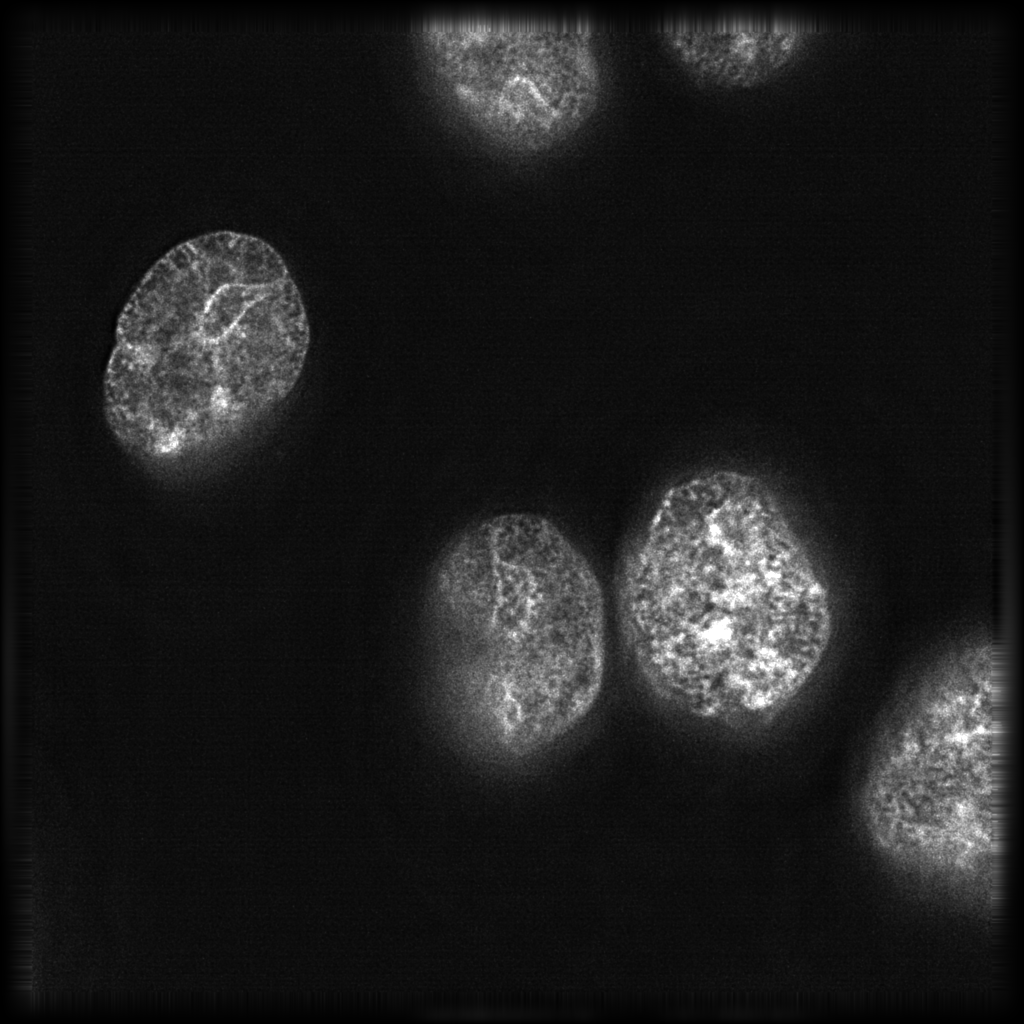

Supplement: Supplementary file 2 — Source data Fig. 1 [file 44318_2026_772_MOESM2_ESM.zip › Figure 1/1B/image_DNA.tif]

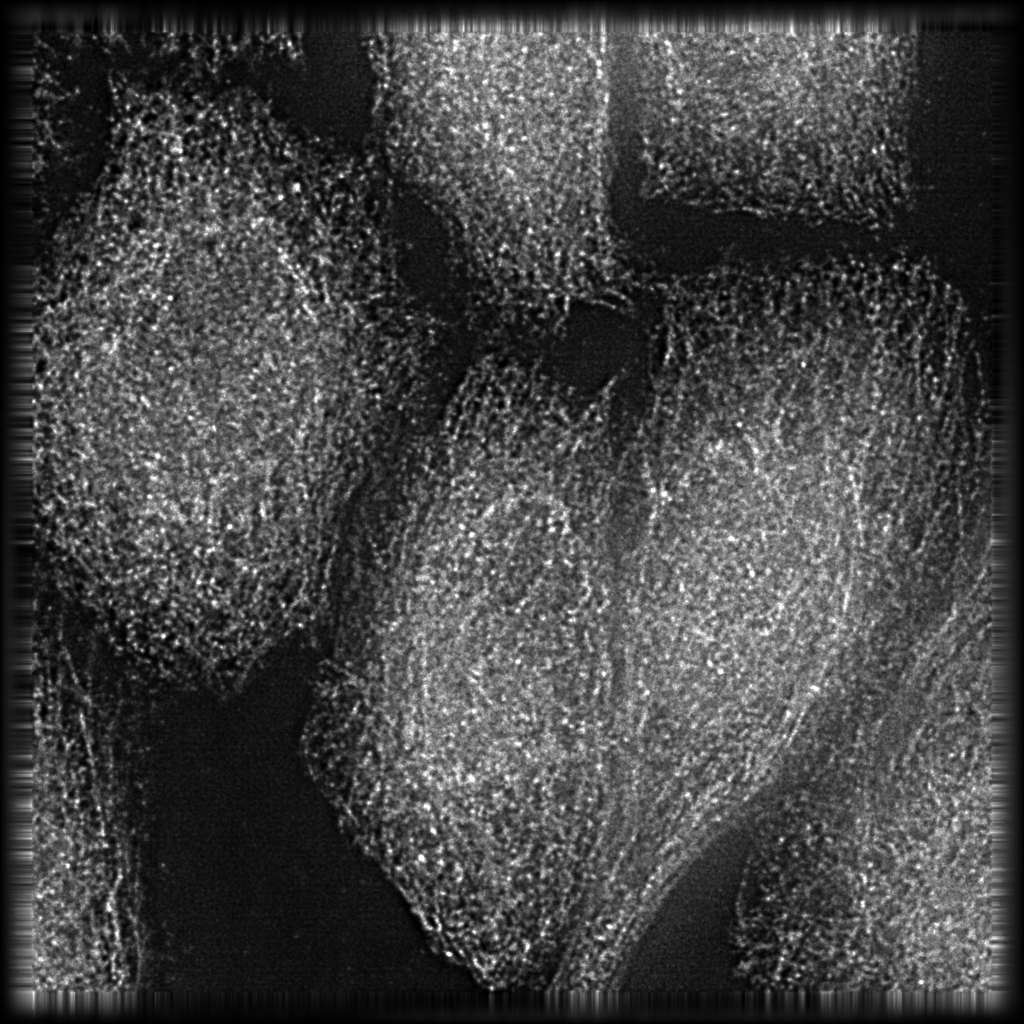

Supplement: Supplementary file 2 — Source data Fig. 1 [file 44318_2026_772_MOESM2_ESM.zip › Figure 1/1B/image_MTs.tif]

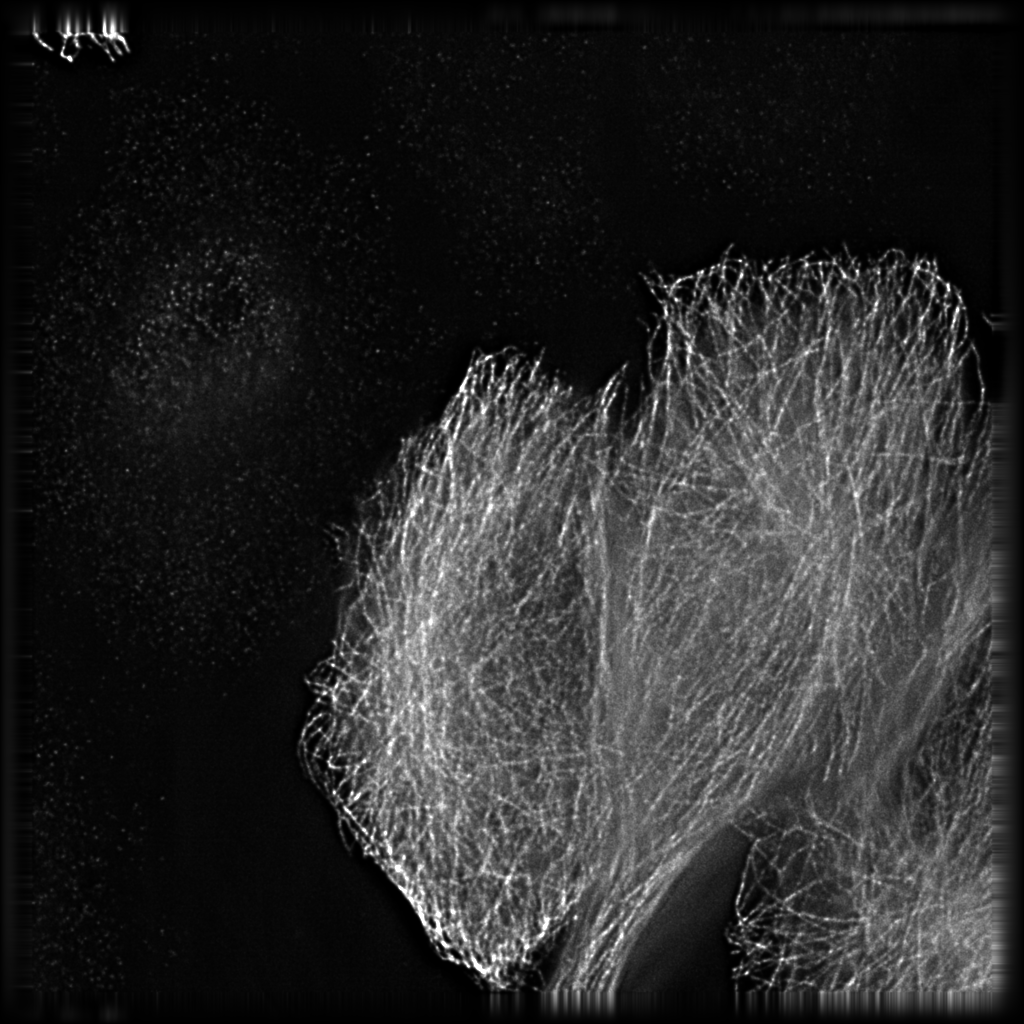

Supplement: Supplementary file 2 — Source data Fig. 1 [file 44318_2026_772_MOESM2_ESM.zip › Figure 1/1B/image_dC2-a-tubulin.tif]

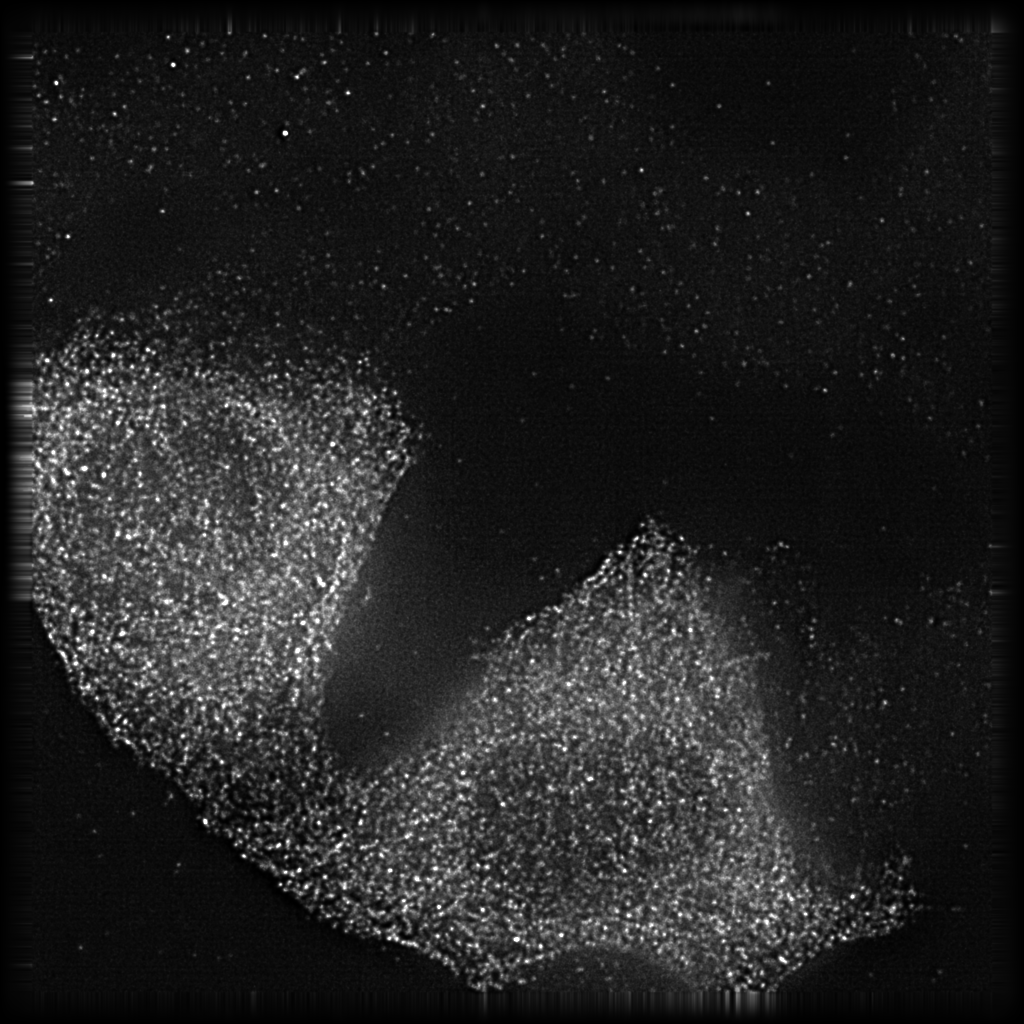

Supplement: Supplementary file 2 — Source data Fig. 1 [file 44318_2026_772_MOESM2_ESM.zip › Figure 1/1E/image_MATCAP1.tif]

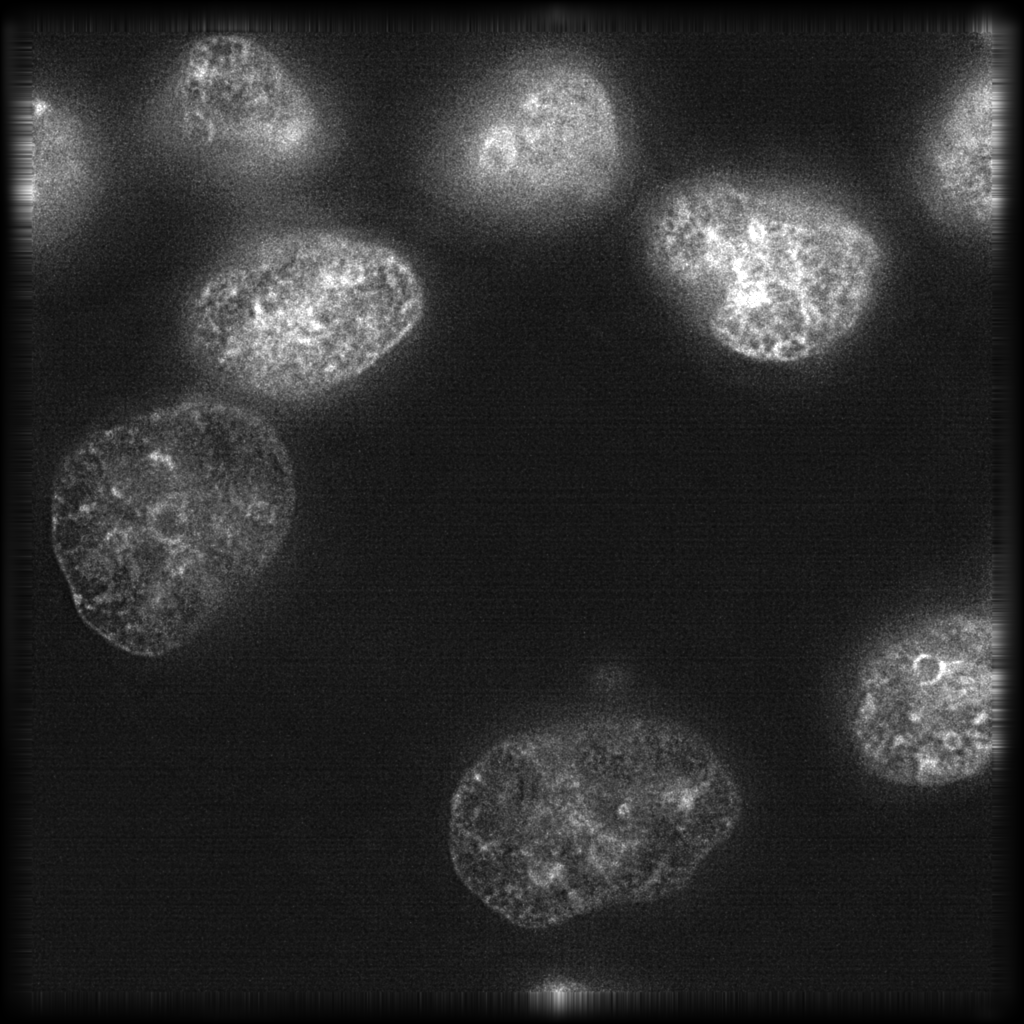

Supplement: Supplementary file 2 — Source data Fig. 1 [file 44318_2026_772_MOESM2_ESM.zip › Figure 1/1E/image_DNA.tif]

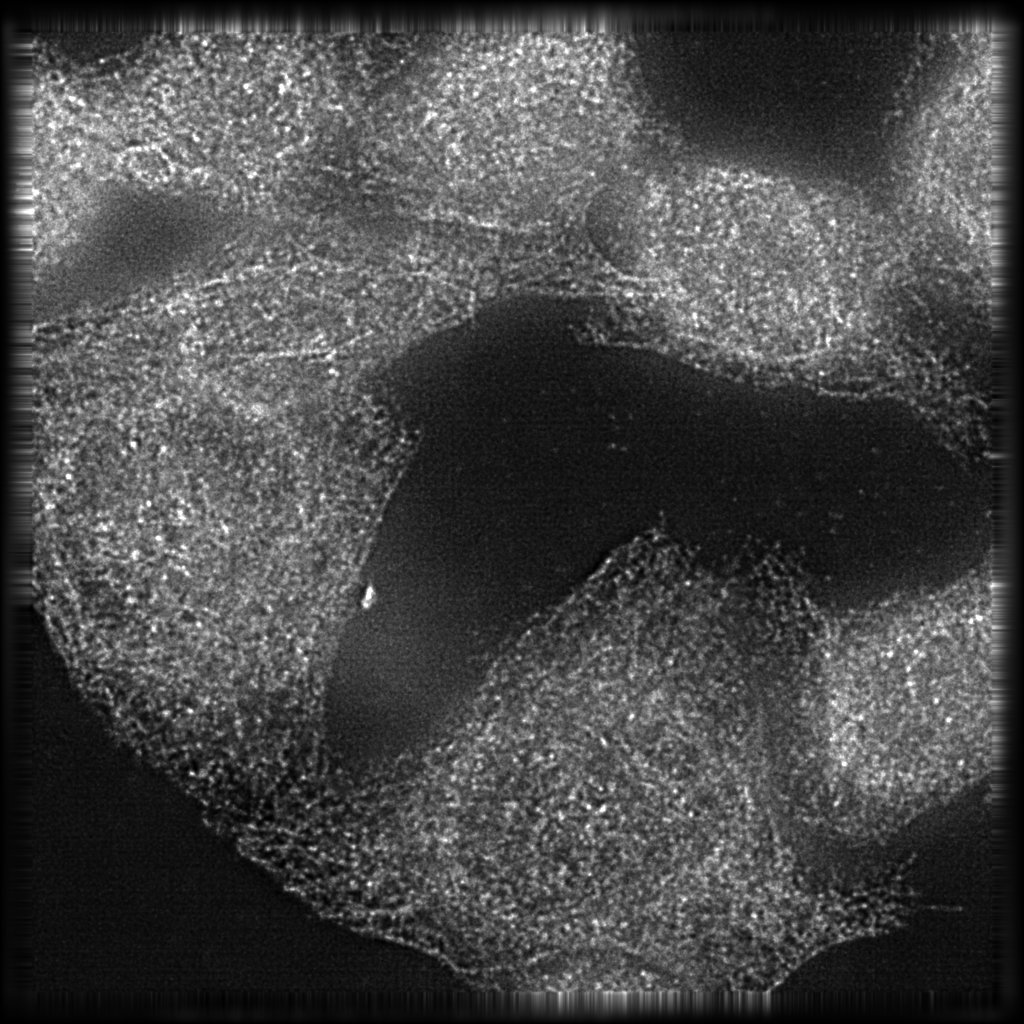

Supplement: Supplementary file 2 — Source data Fig. 1 [file 44318_2026_772_MOESM2_ESM.zip › Figure 1/1E/image_MTs.tif]

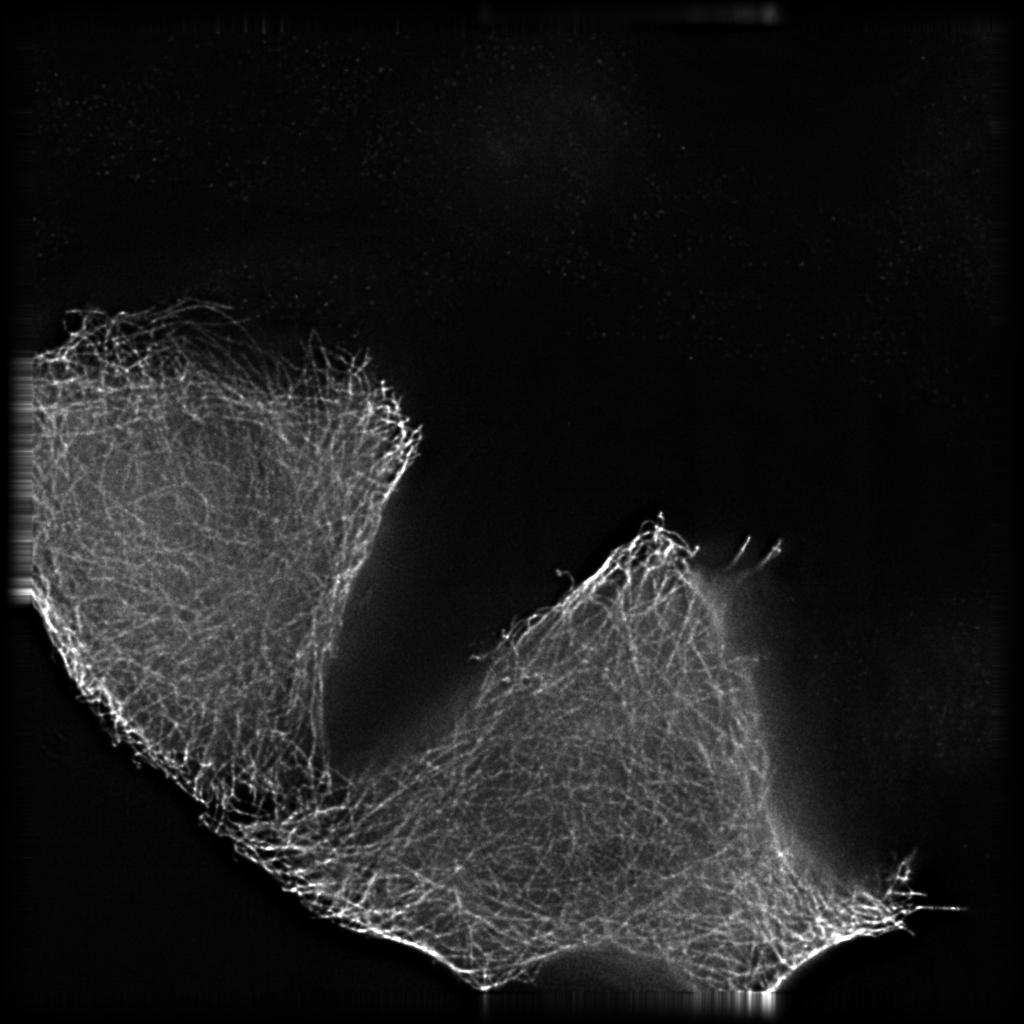

Supplement: Supplementary file 2 — Source data Fig. 1 [file 44318_2026_772_MOESM2_ESM.zip › Figure 1/1E/image_dC2-a-tubulin.tif]

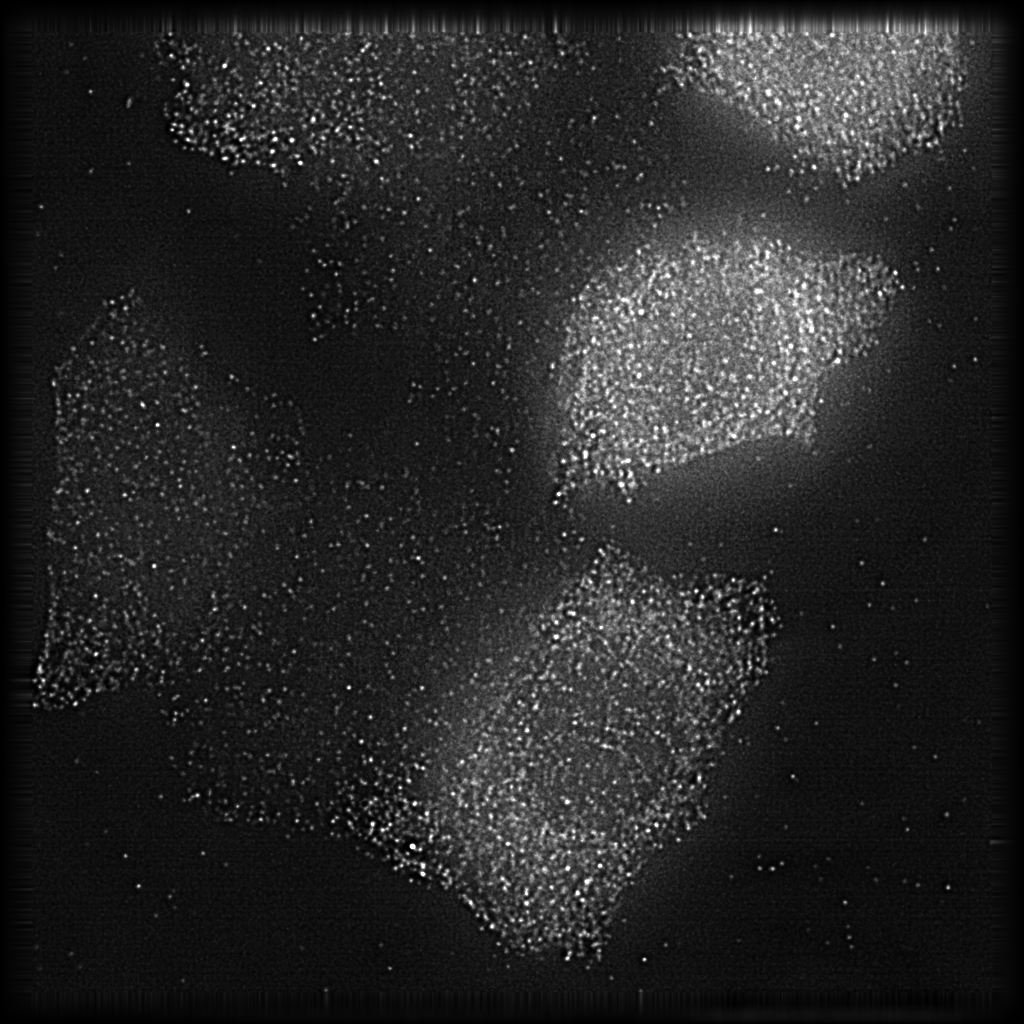

Supplement: Supplementary file 2 — Source data Fig. 1 [file 44318_2026_772_MOESM2_ESM.zip › Figure 1/1D/image_MATCAP1.tif]

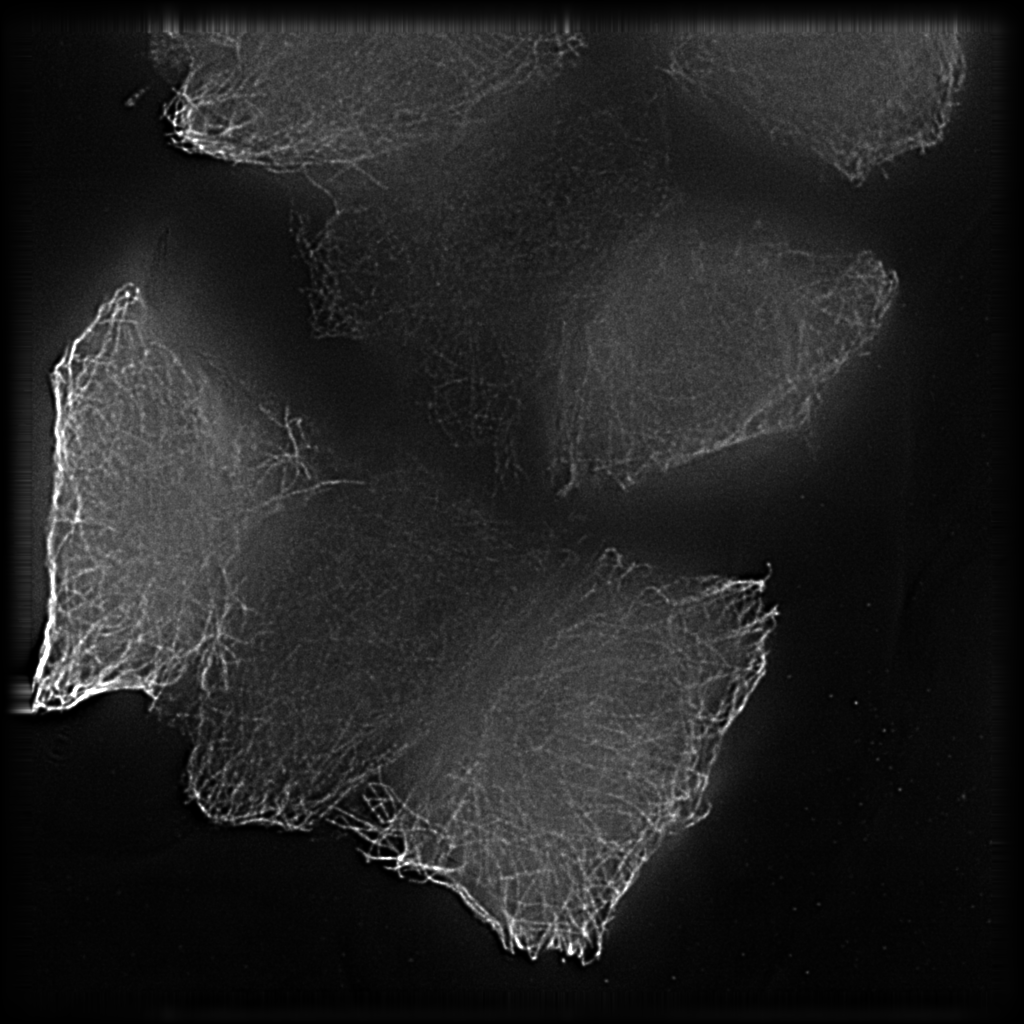

Supplement: Supplementary file 2 — Source data Fig. 1 [file 44318_2026_772_MOESM2_ESM.zip › Figure 1/1D/image_dY-a-tubulin.tif]

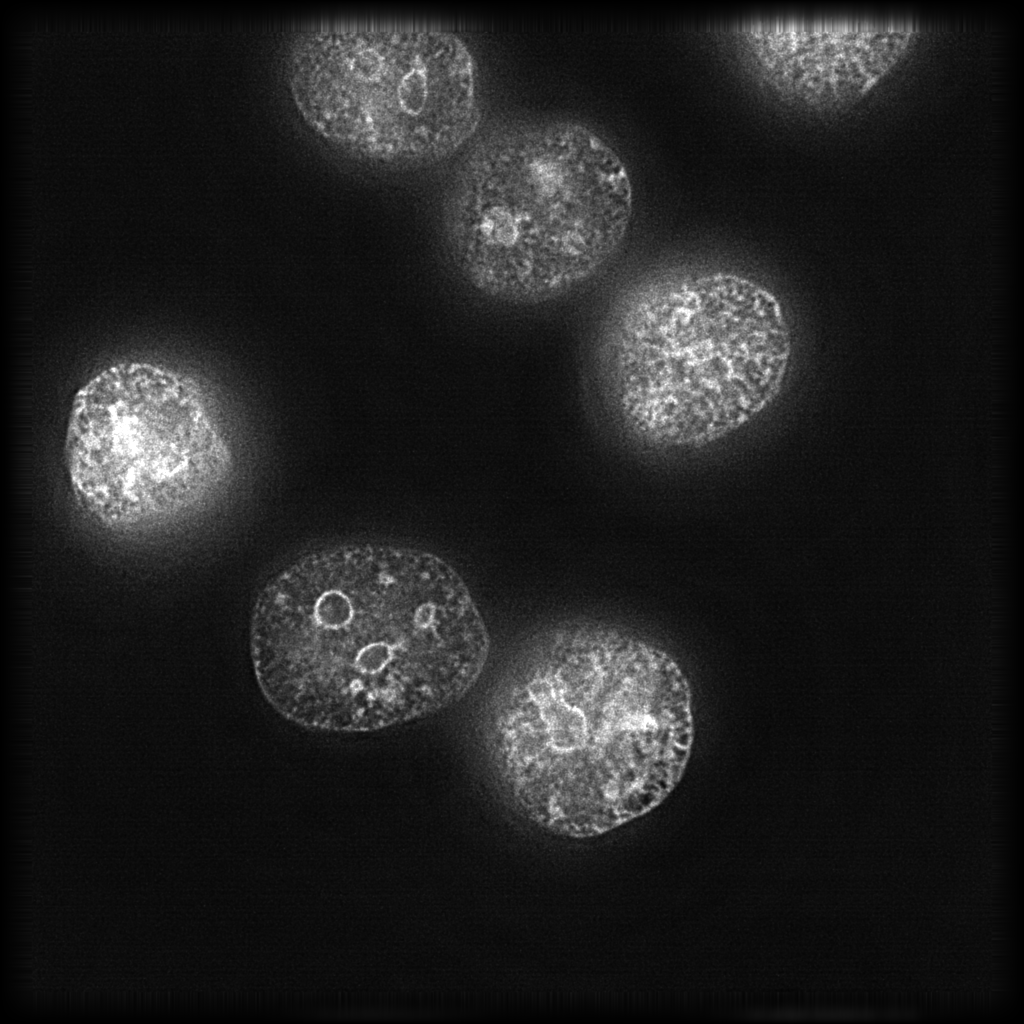

Supplement: Supplementary file 2 — Source data Fig. 1 [file 44318_2026_772_MOESM2_ESM.zip › Figure 1/1D/image_DNA.tif]

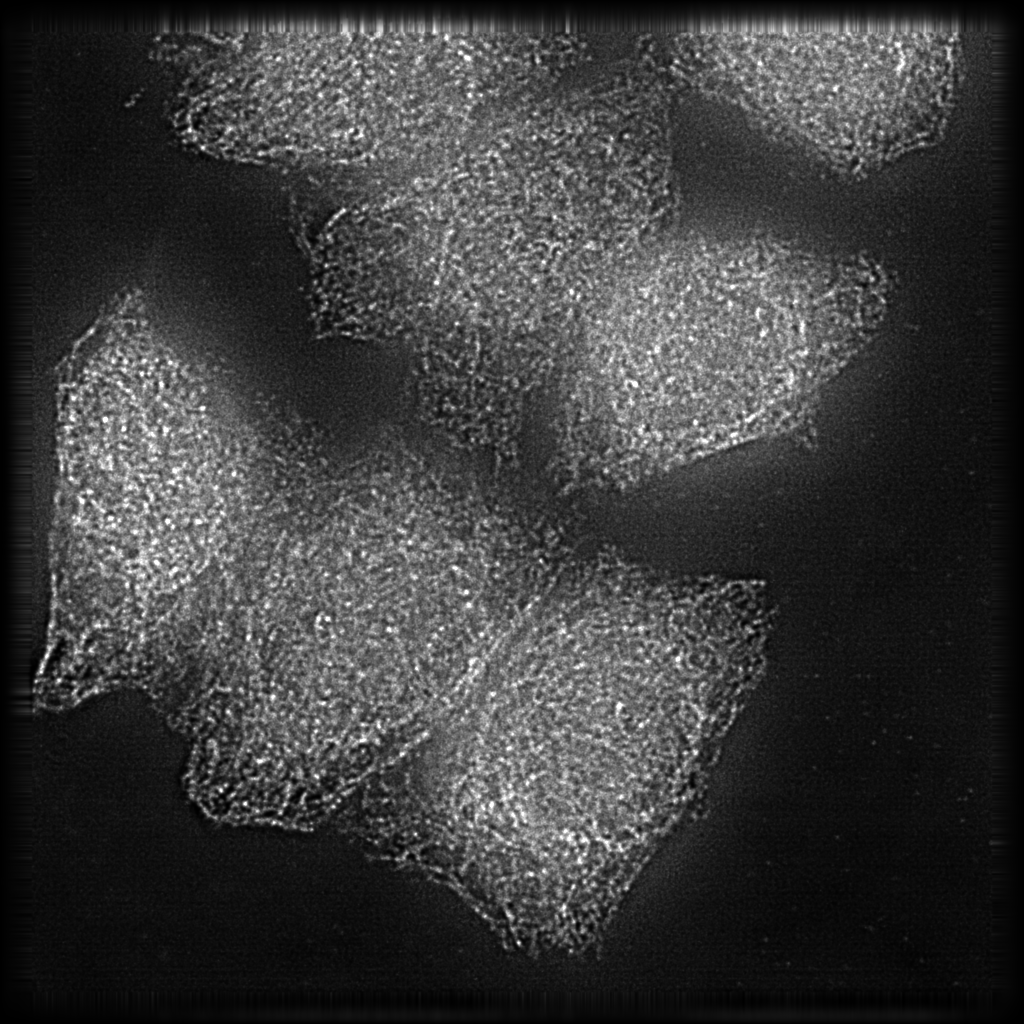

Supplement: Supplementary file 2 — Source data Fig. 1 [file 44318_2026_772_MOESM2_ESM.zip › Figure 1/1D/image_MTs.tif]

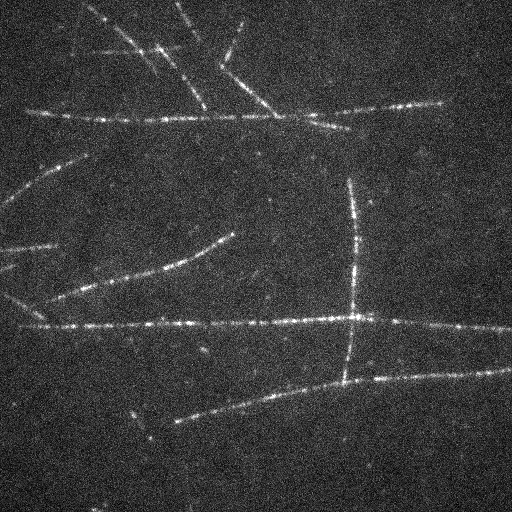

Supplement: Supplementary file 3 — Source data Fig. 2 [file 44318_2026_772_MOESM3_ESM.zip › source data_Figure 2/2G/image_dC2_MATCAP1_10 min.tif]

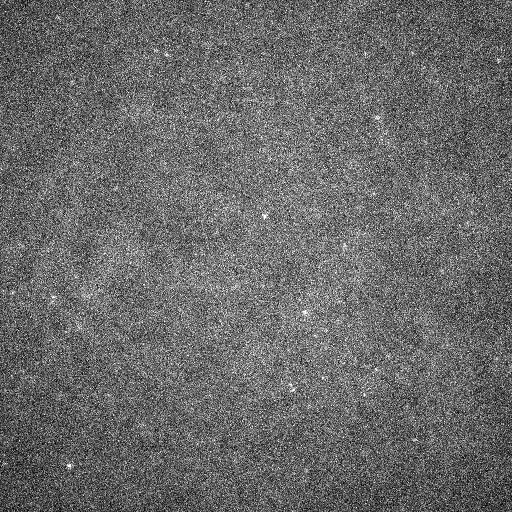

Supplement: Supplementary file 3 — Source data Fig. 2 [file 44318_2026_772_MOESM3_ESM.zip › source data_Figure 2/2G/image_dC2_MATCAP1_0 min.tif]

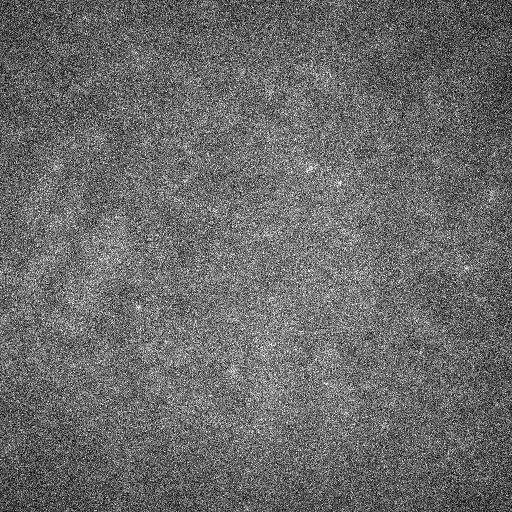

Supplement: Supplementary file 3 — Source data Fig. 2 [file 44318_2026_772_MOESM3_ESM.zip › source data_Figure 2/2G/image_dC2_VASH1_original_10 min.tif]

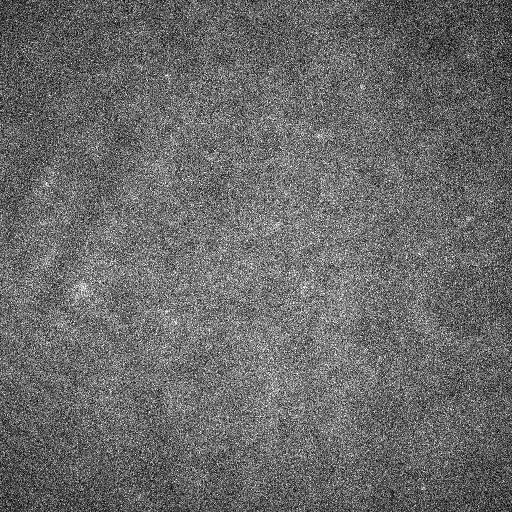

Supplement: Supplementary file 3 — Source data Fig. 2 [file 44318_2026_772_MOESM3_ESM.zip › source data_Figure 2/2G/image_dC2_VASH1_original_0 min.tif]

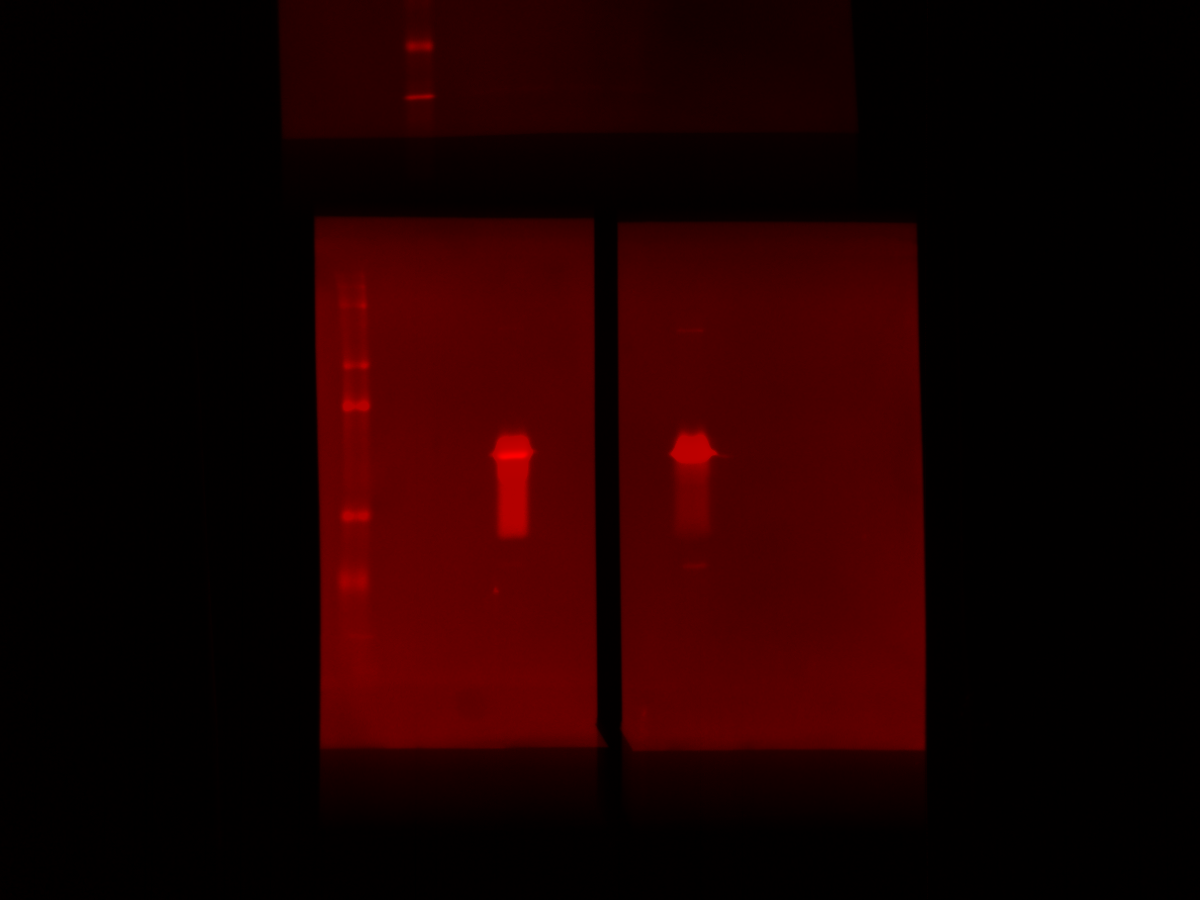

Supplement: Supplementary file 3 — Source data Fig. 2 [file 44318_2026_772_MOESM3_ESM.zip › source data_Figure 2/2F/western_dC2-Fab_raw.tif]

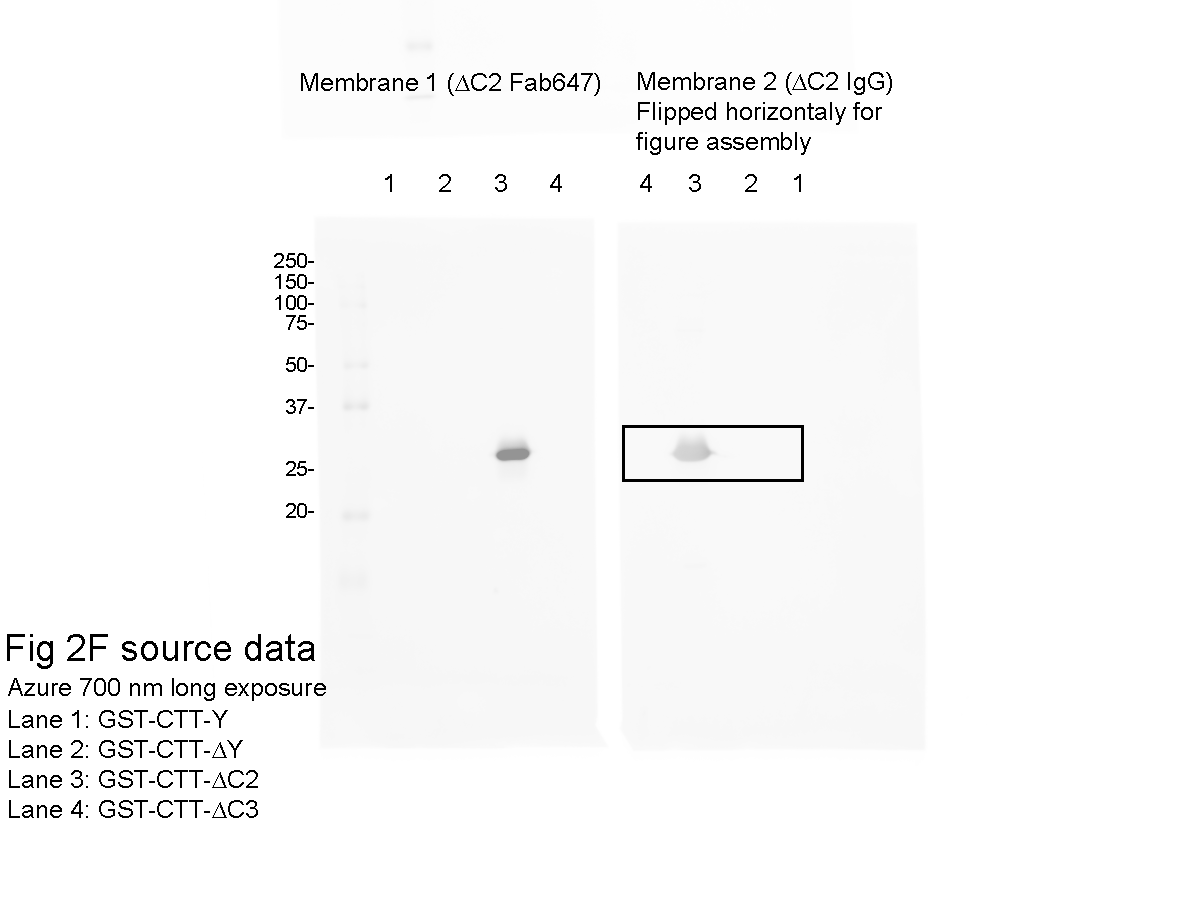

Supplement: Supplementary file 3 — Source data Fig. 2 [file 44318_2026_772_MOESM3_ESM.zip › source data_Figure 2/2F/western_IgG_labeled.tif]

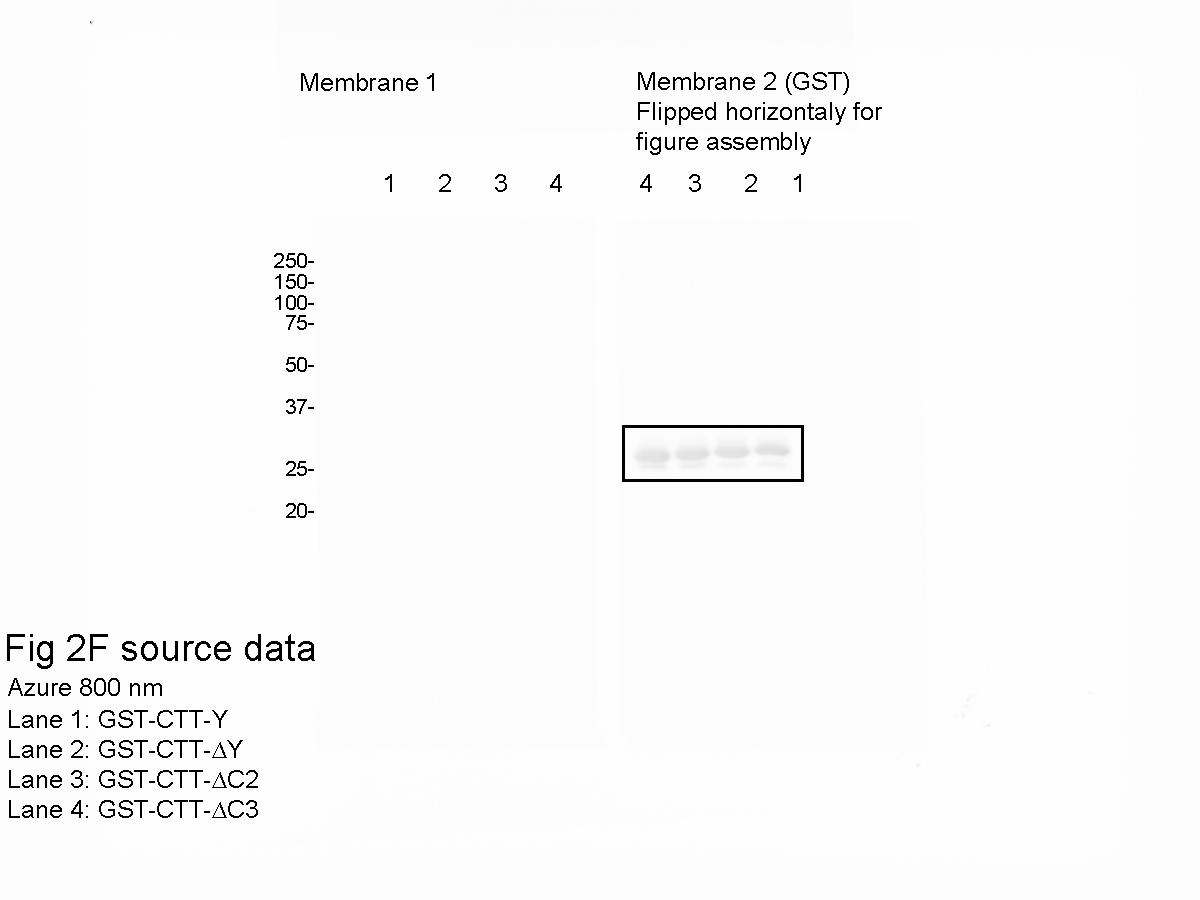

Supplement: Supplementary file 3 — Source data Fig. 2 [file 44318_2026_772_MOESM3_ESM.zip › source data_Figure 2/2F/western_GST_labeled.tif]

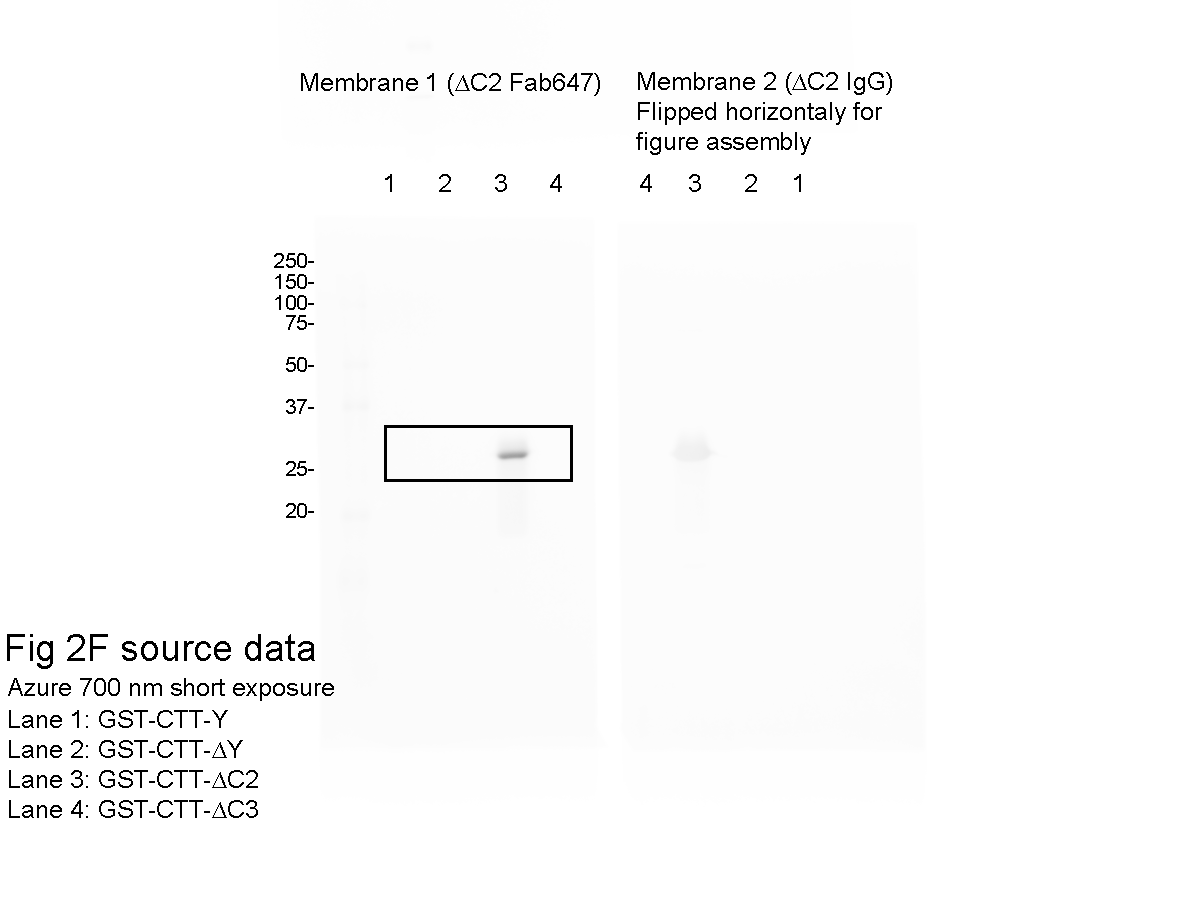

Supplement: Supplementary file 3 — Source data Fig. 2 [file 44318_2026_772_MOESM3_ESM.zip › source data_Figure 2/2F/western_dC2-Fab_labeled.tif]

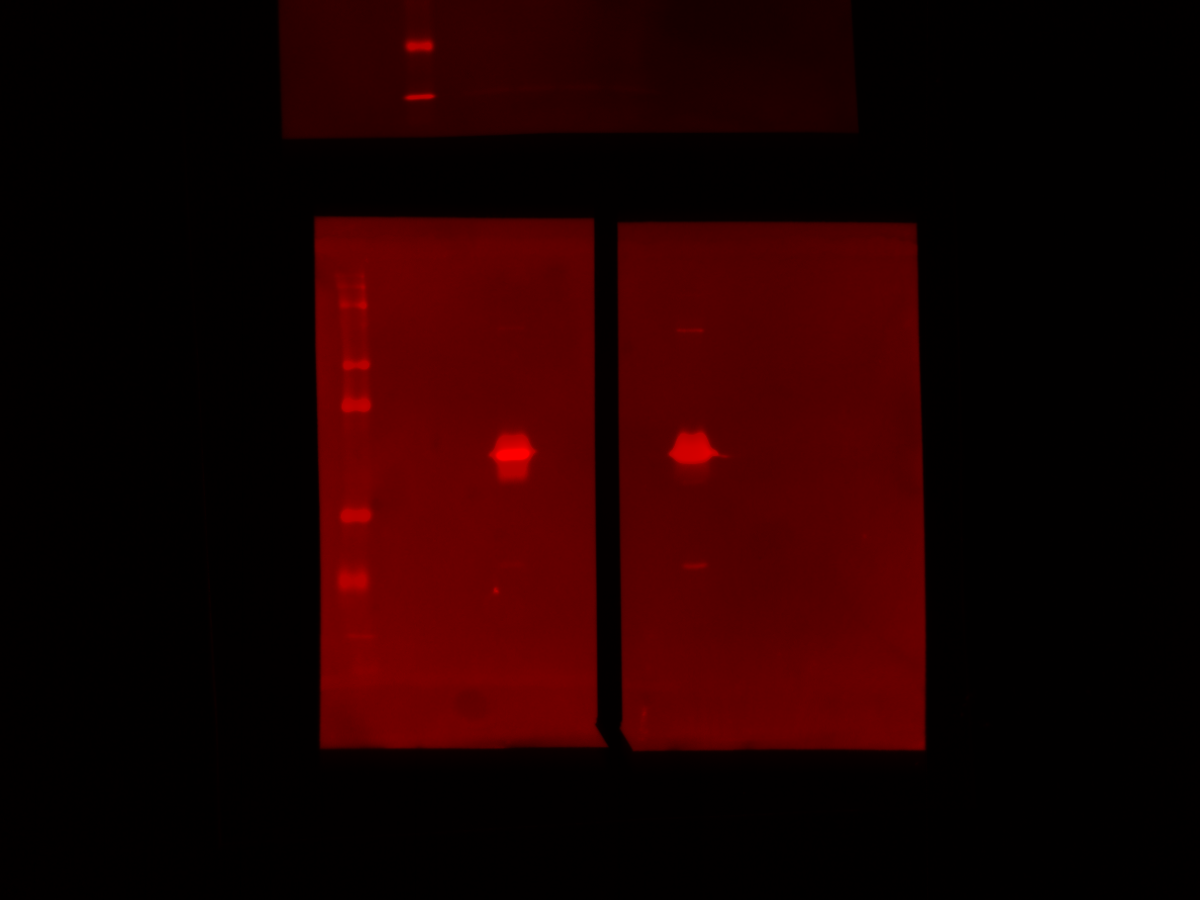

Supplement: Supplementary file 3 — Source data Fig. 2 [file 44318_2026_772_MOESM3_ESM.zip › source data_Figure 2/2F/western_IgG_raw.tif]

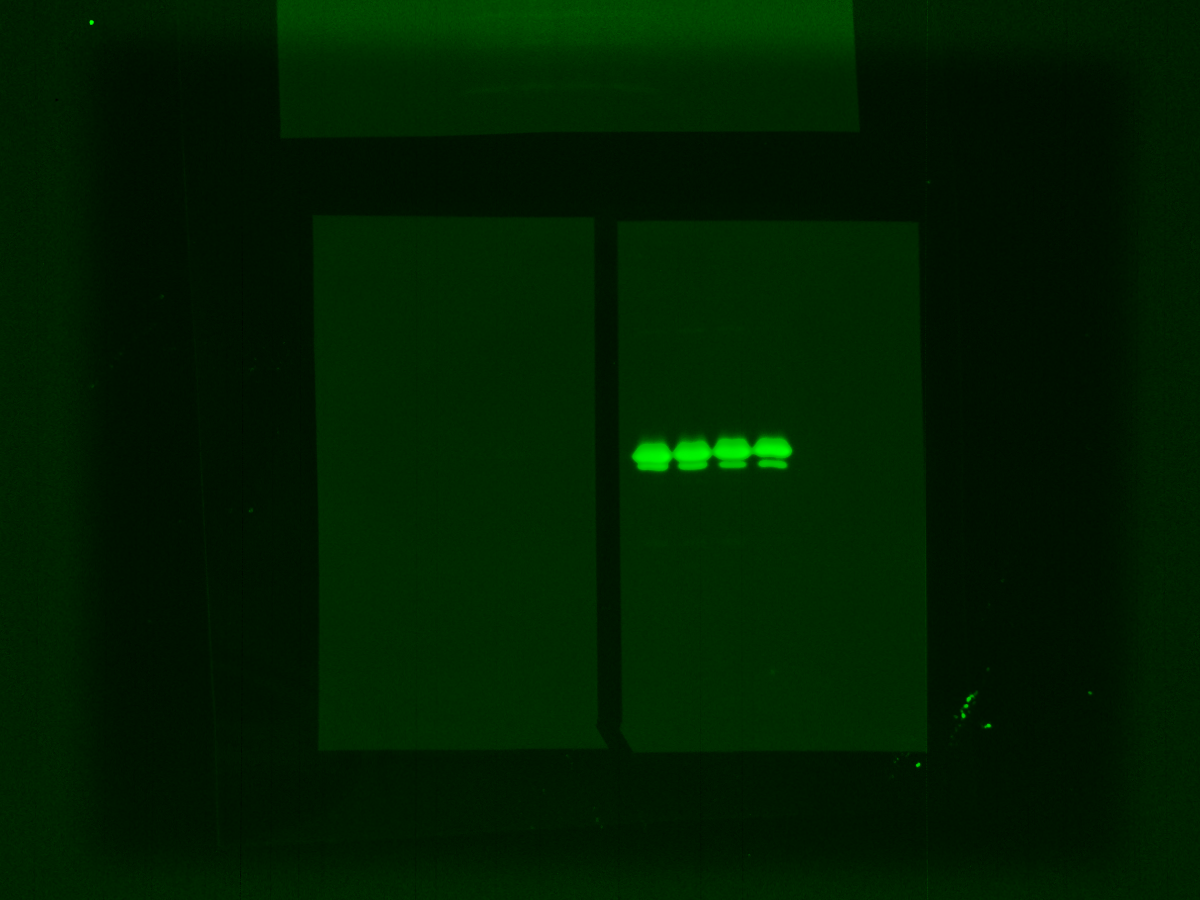

Supplement: Supplementary file 3 — Source data Fig. 2 [file 44318_2026_772_MOESM3_ESM.zip › source data_Figure 2/2F/western_GST_raw.tif]

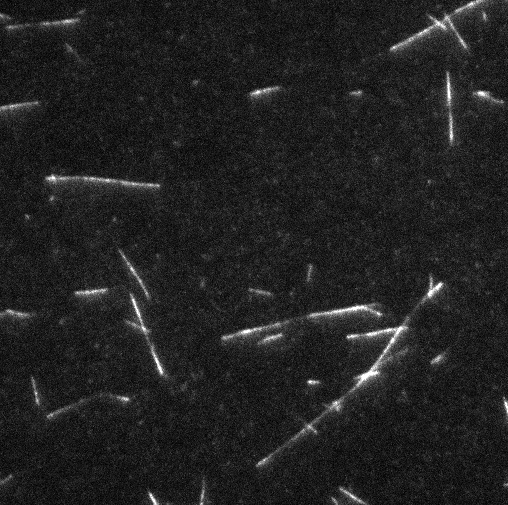

Supplement: Supplementary file 3 — Source data Fig. 2 [file 44318_2026_772_MOESM3_ESM.zip › source data_Figure 2/2B/image_dY_VASH1,10 min.tif]

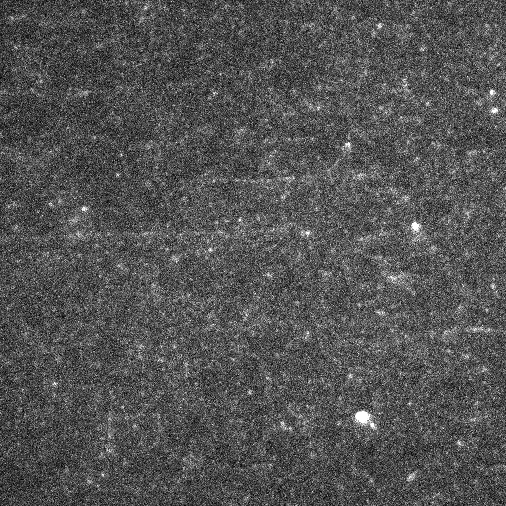

Supplement: Supplementary file 3 — Source data Fig. 2 [file 44318_2026_772_MOESM3_ESM.zip › source data_Figure 2/2B/image_dY_MATCAP1,0 min.tif]

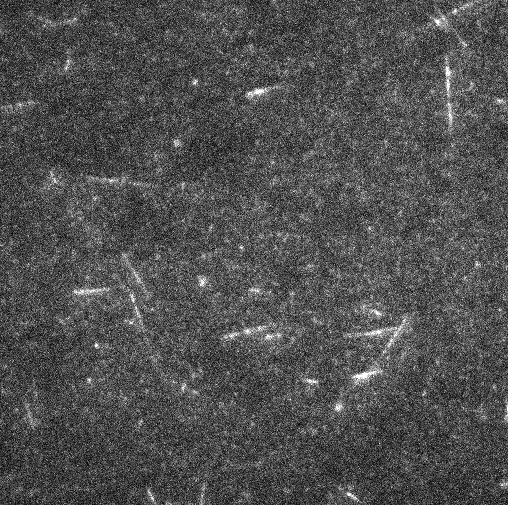

Supplement: Supplementary file 3 — Source data Fig. 2 [file 44318_2026_772_MOESM3_ESM.zip › source data_Figure 2/2B/image_dY_VASH1,0 min.tif]

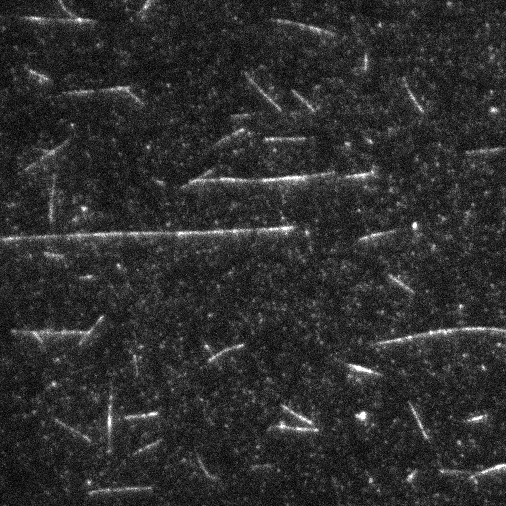

Supplement: Supplementary file 3 — Source data Fig. 2 [file 44318_2026_772_MOESM3_ESM.zip › source data_Figure 2/2B/image_dY_MATCAP1,10 min.tif]

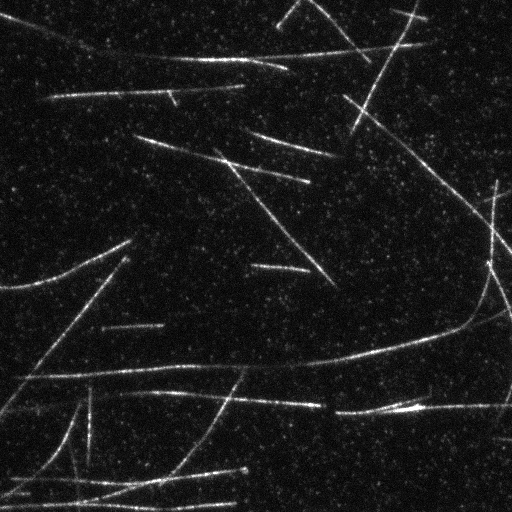

Supplement: Supplementary file 4 — Source data Fig. 3 [file 44318_2026_772_MOESM4_ESM.zip › source data_Figure 3/3B/image_dY, dC2_10 min merge.tif]

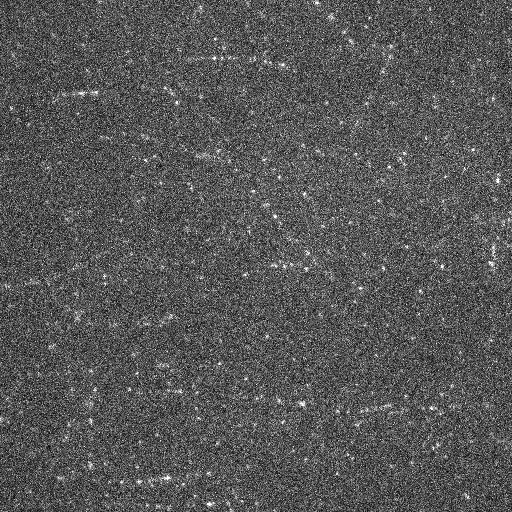

Supplement: Supplementary file 4 — Source data Fig. 3 [file 44318_2026_772_MOESM4_ESM.zip › source data_Figure 3/3B/image_dY, dC2_0 min merge.tif]

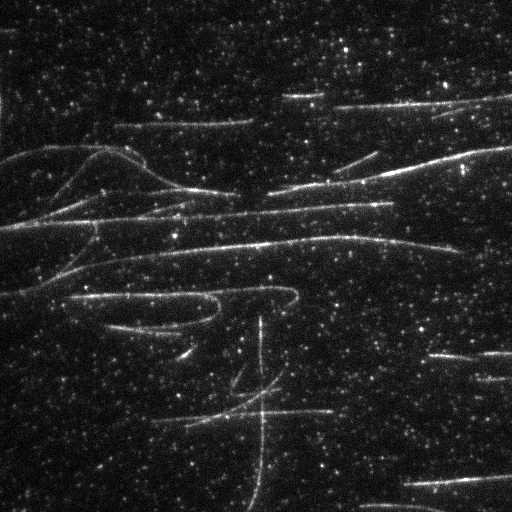

Supplement: Supplementary file 5 — Source data Fig. 4 [file 44318_2026_772_MOESM5_ESM.zip › source data_Figure 4/4D/image_WT on dY-MTs_merge.tif]

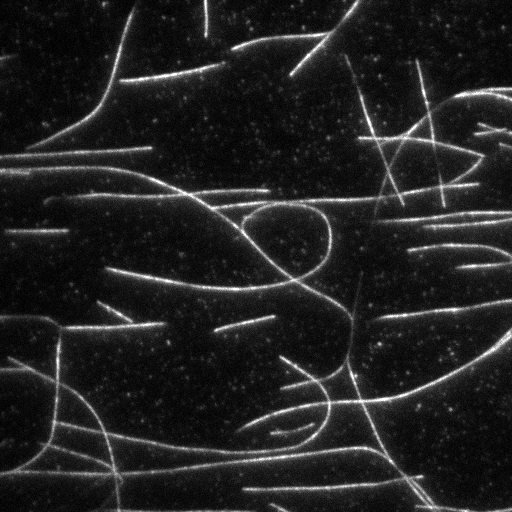

Supplement: Supplementary file 5 — Source data Fig. 4 [file 44318_2026_772_MOESM5_ESM.zip › source data_Figure 4/4D/image_E281Q on Y-MTs_merge.tif]

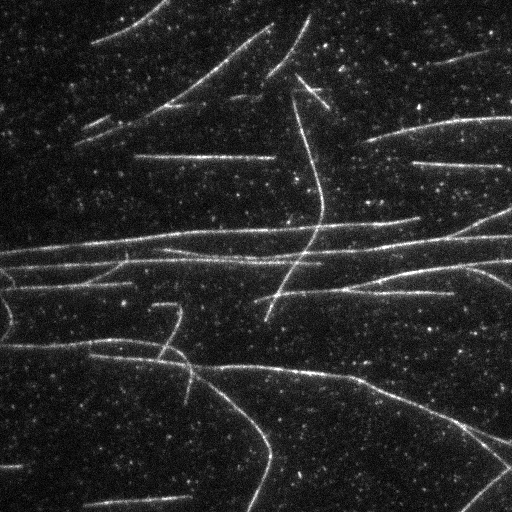

Supplement: Supplementary file 5 — Source data Fig. 4 [file 44318_2026_772_MOESM5_ESM.zip › source data_Figure 4/4D/image_WT on Y-MTs_merge.tif]

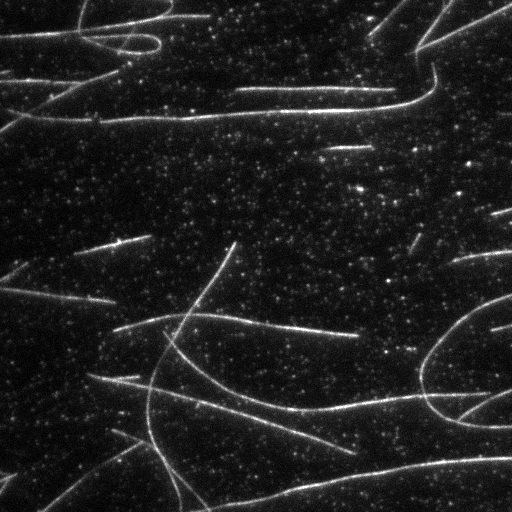

Supplement: Supplementary file 5 — Source data Fig. 4 [file 44318_2026_772_MOESM5_ESM.zip › source data_Figure 4/4D/image_E281Q on dY-MTs_merge.tif]

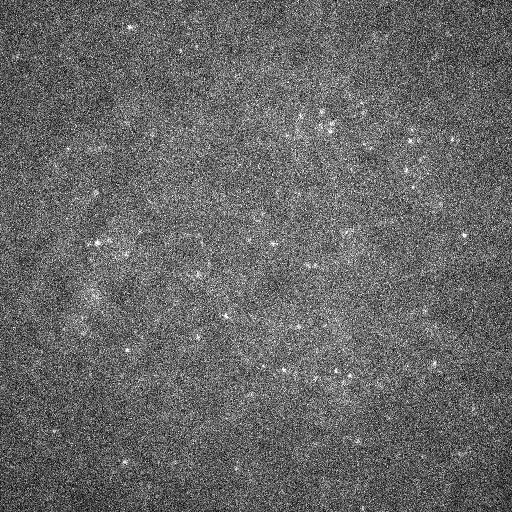

Supplement: Supplementary file 5 — Source data Fig. 4 [file 44318_2026_772_MOESM5_ESM.zip › source data_Figure 4/4F/image_dC2_MATCAP1 on dY-MTs_0 min.tif]

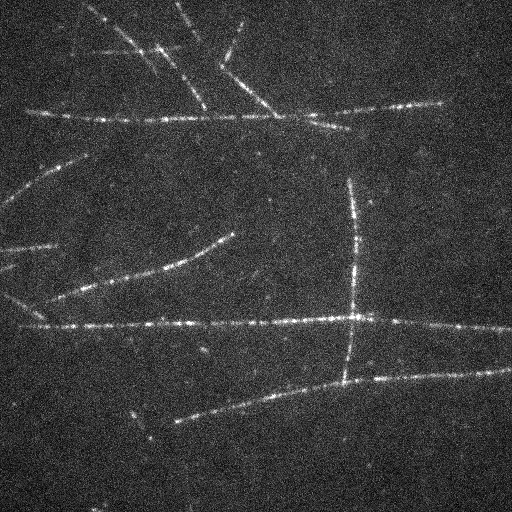

Supplement: Supplementary file 5 — Source data Fig. 4 [file 44318_2026_772_MOESM5_ESM.zip › source data_Figure 4/4F/image_dC2_MATCAP1 on Y-MTs_10 min.tif]

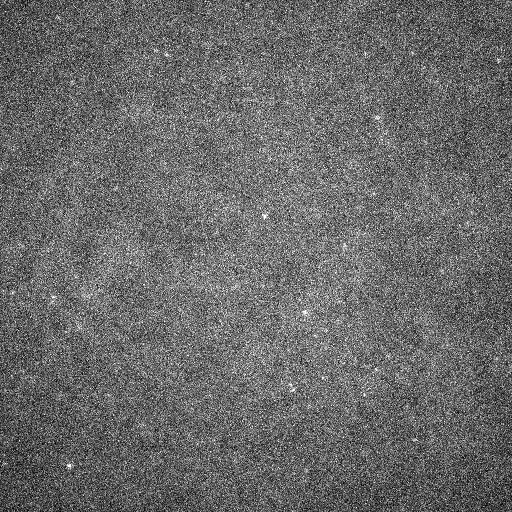

Supplement: Supplementary file 5 — Source data Fig. 4 [file 44318_2026_772_MOESM5_ESM.zip › source data_Figure 4/4F/image_dC2_MATCAP1 on Y-MTs_0 min.tif]

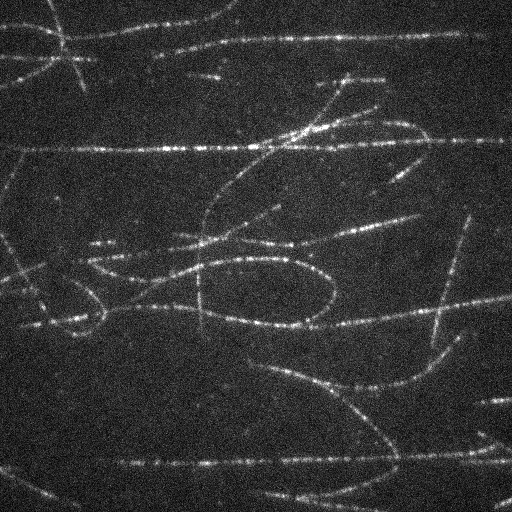

Supplement: Supplementary file 5 — Source data Fig. 4 [file 44318_2026_772_MOESM5_ESM.zip › source data_Figure 4/4F/image_dC2_MATCAP1 on dY-MTs_10 min.tif]

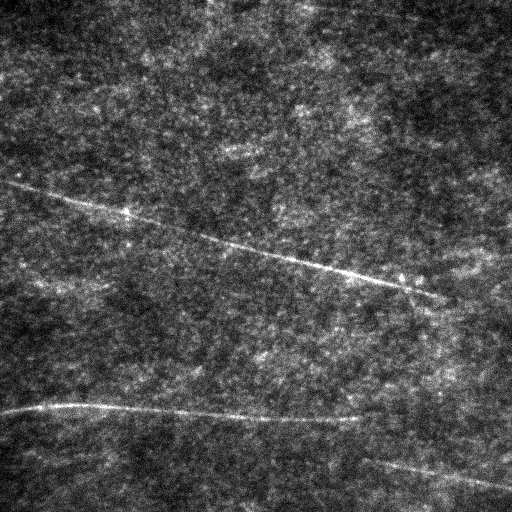

Supplement: Supplementary file 6 — Source data Fig. 5 [file 44318_2026_772_MOESM6_ESM.zip › source data_Figure 5/5G/image_with ATP, KIF5C_merge.tif]

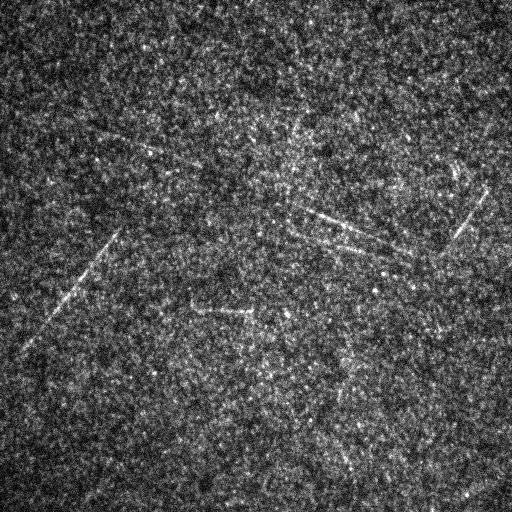

Supplement: Supplementary file 6 — Source data Fig. 5 [file 44318_2026_772_MOESM6_ESM.zip › source data_Figure 5/5G/image_with no KIF5C_merge.tif]

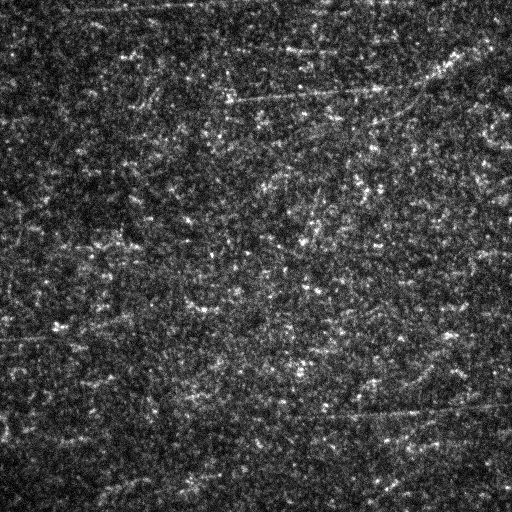

Supplement: Supplementary file 6 — Source data Fig. 5 [file 44318_2026_772_MOESM6_ESM.zip › source data_Figure 5/5G/image_with ADP, KIF5C_merge.tif]

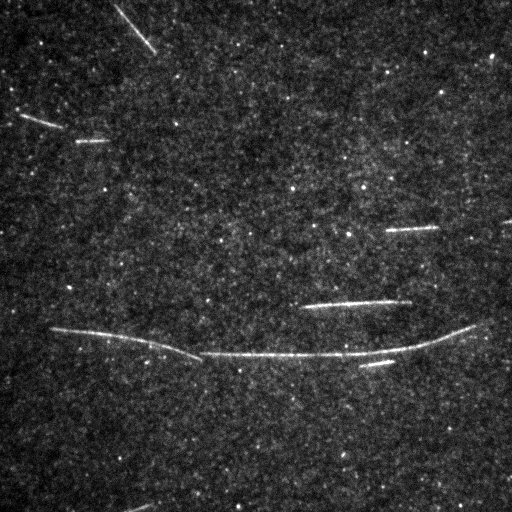

Supplement: Supplementary file 6 — Source data Fig. 5 [file 44318_2026_772_MOESM6_ESM.zip › source data_Figure 5/5C/image_on GDP-MTs with DMSO_merge.tif]

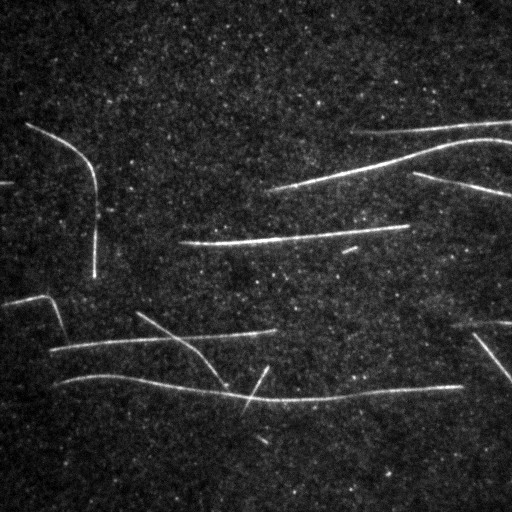

Supplement: Supplementary file 6 — Source data Fig. 5 [file 44318_2026_772_MOESM6_ESM.zip › source data_Figure 5/5C/image_on GDP-MTs with Taxol_merge.tif]

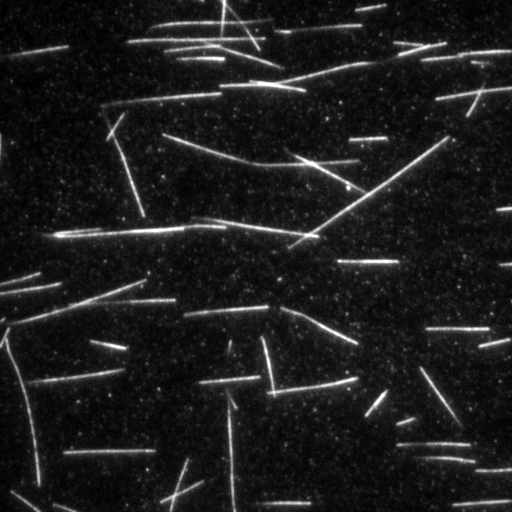

Supplement: Supplementary file 6 — Source data Fig. 5 [file 44318_2026_772_MOESM6_ESM.zip › source data_Figure 5/5C/image_on GMPCPP-MTs with DMSO_merge.tif]

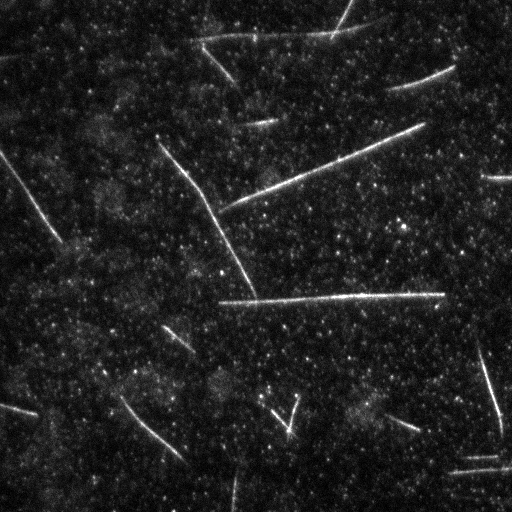

Supplement: Supplementary file 6 — Source data Fig. 5 [file 44318_2026_772_MOESM6_ESM.zip › source data_Figure 5/5E/image_E281Q on Taxol-Y-MTs_merge.tif]

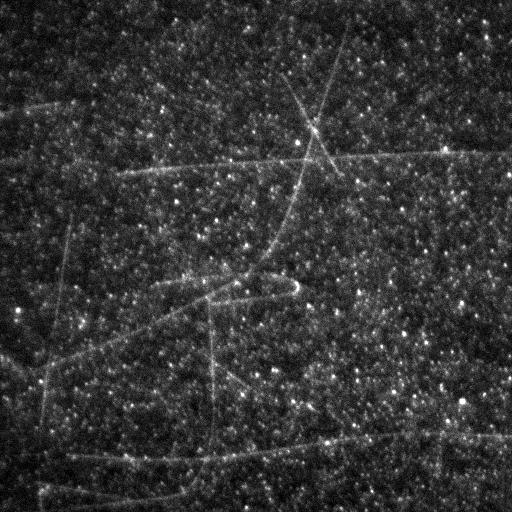

Supplement: Supplementary file 6 — Source data Fig. 5 [file 44318_2026_772_MOESM6_ESM.zip › source data_Figure 5/5E/image_E281Q on PelA-Y-MTs_merge.tif]

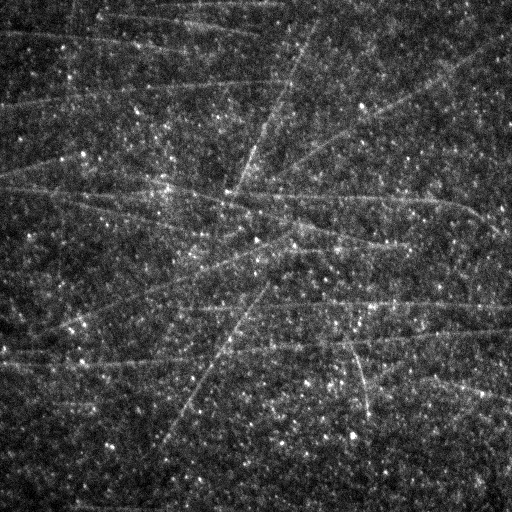

Supplement: Supplementary file 6 — Source data Fig. 5 [file 44318_2026_772_MOESM6_ESM.zip › source data_Figure 5/5E/image_WT on PelA-Y-MTs_merge.tif]

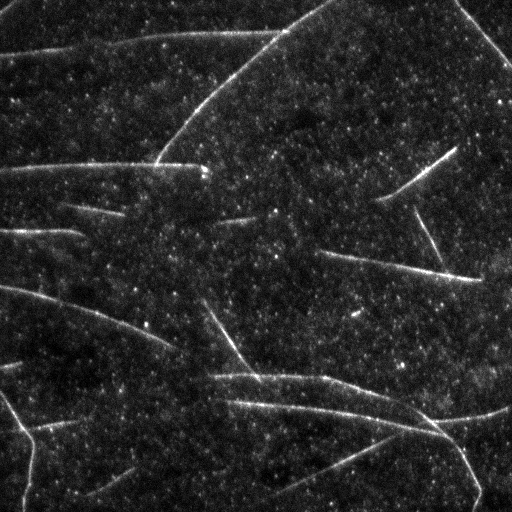

Supplement: Supplementary file 6 — Source data Fig. 5 [file 44318_2026_772_MOESM6_ESM.zip › source data_Figure 5/5E/image_WT on Taxol-Y-MTs_merge.tif]

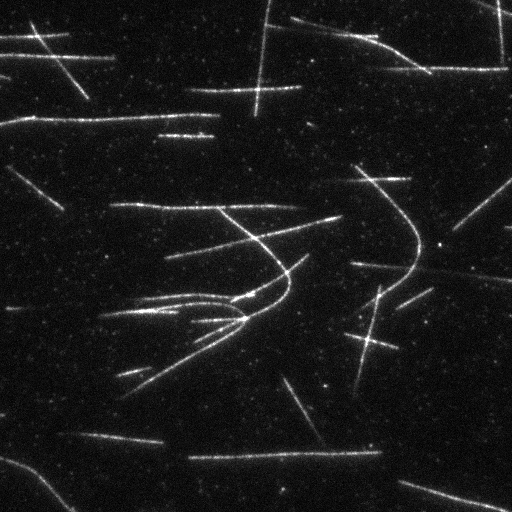

Supplement: Supplementary file 7 — Source data Fig. 6 [file 44318_2026_772_MOESM7_ESM.zip › source data_Figure 6/6B/image_GMPCPP-MTs with MATCAP1, DMSO_merge.tif]

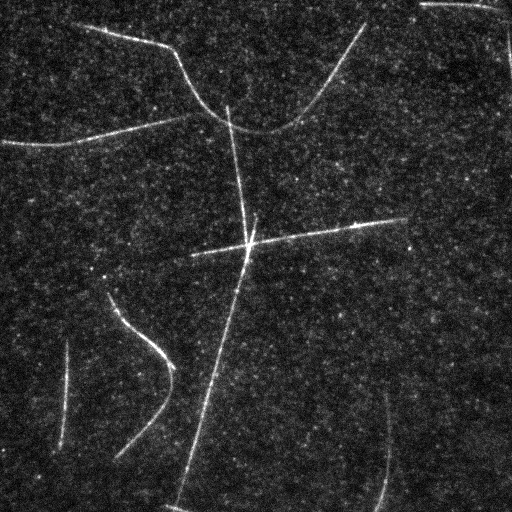

Supplement: Supplementary file 7 — Source data Fig. 6 [file 44318_2026_772_MOESM7_ESM.zip › source data_Figure 6/6B/image_GDP-MTs with MATCAP1, Taxol_merge.tif]

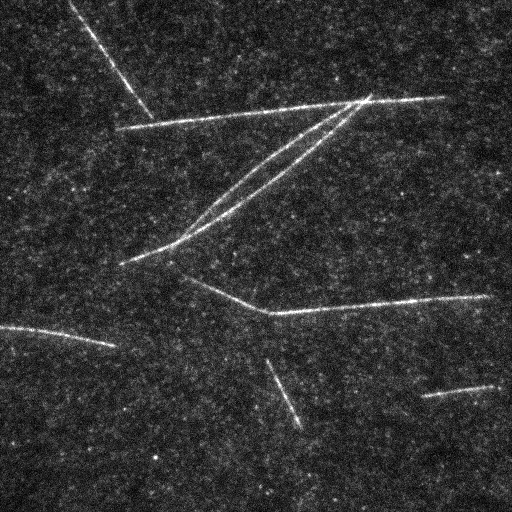

Supplement: Supplementary file 7 — Source data Fig. 6 [file 44318_2026_772_MOESM7_ESM.zip › source data_Figure 6/6B/image_GDP-MTs with MATCAP1, DMSO_merge.tif]

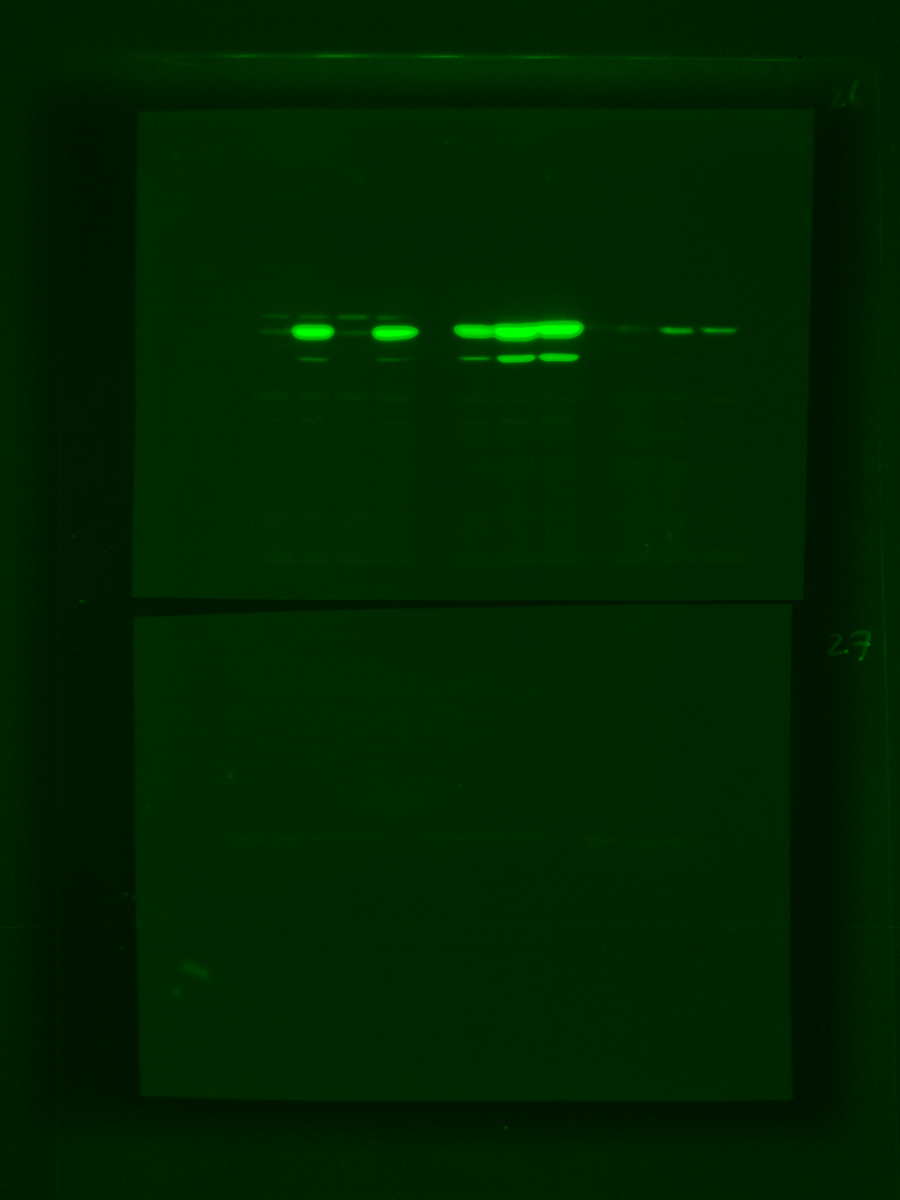

Supplement: Supplementary file 7 — Source data Fig. 6 [file 44318_2026_772_MOESM7_ESM.zip › source data_Figure 6/6D/western_dY-a-tubulin,long exp_raw.tif]

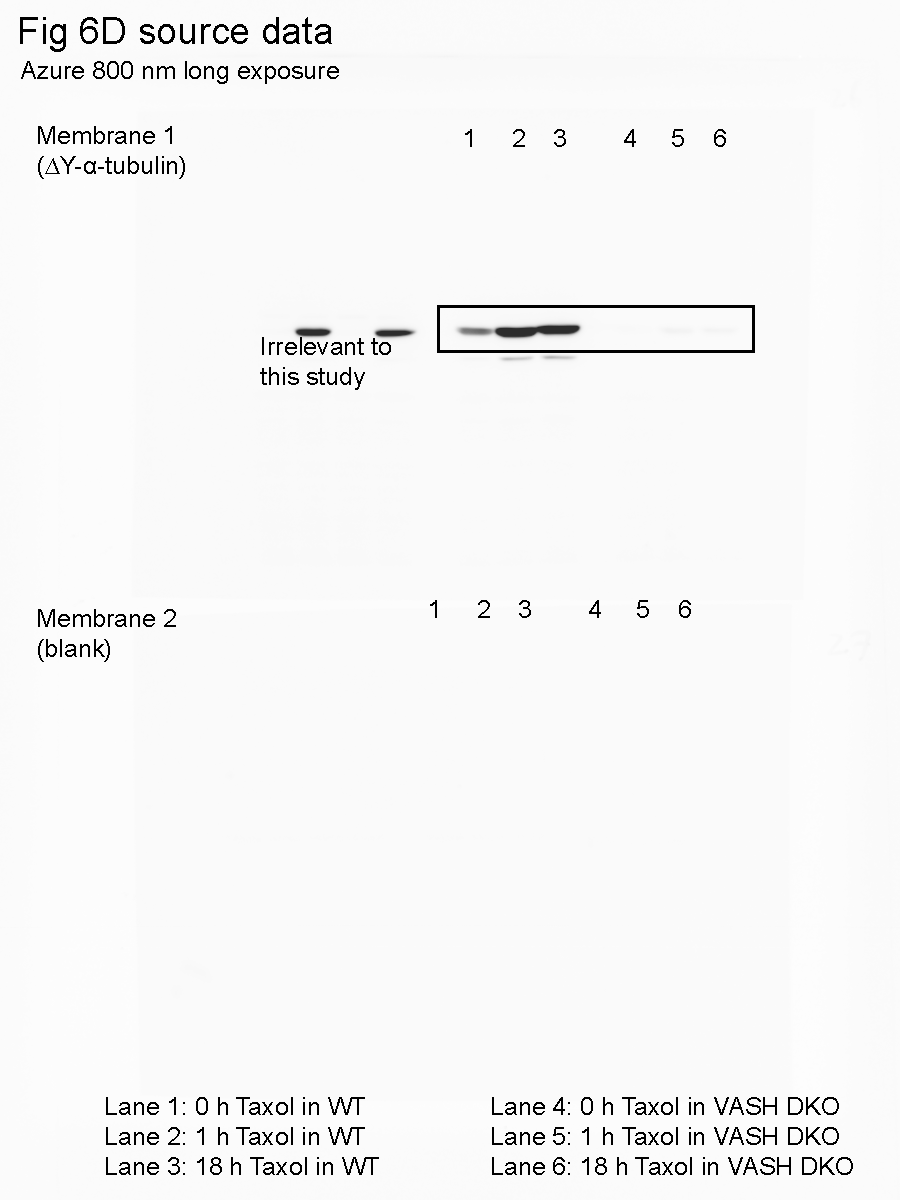

Supplement: Supplementary file 7 — Source data Fig. 6 [file 44318_2026_772_MOESM7_ESM.zip › source data_Figure 6/6D/western_dY-a-tubulin,long exp_labeled.tif]

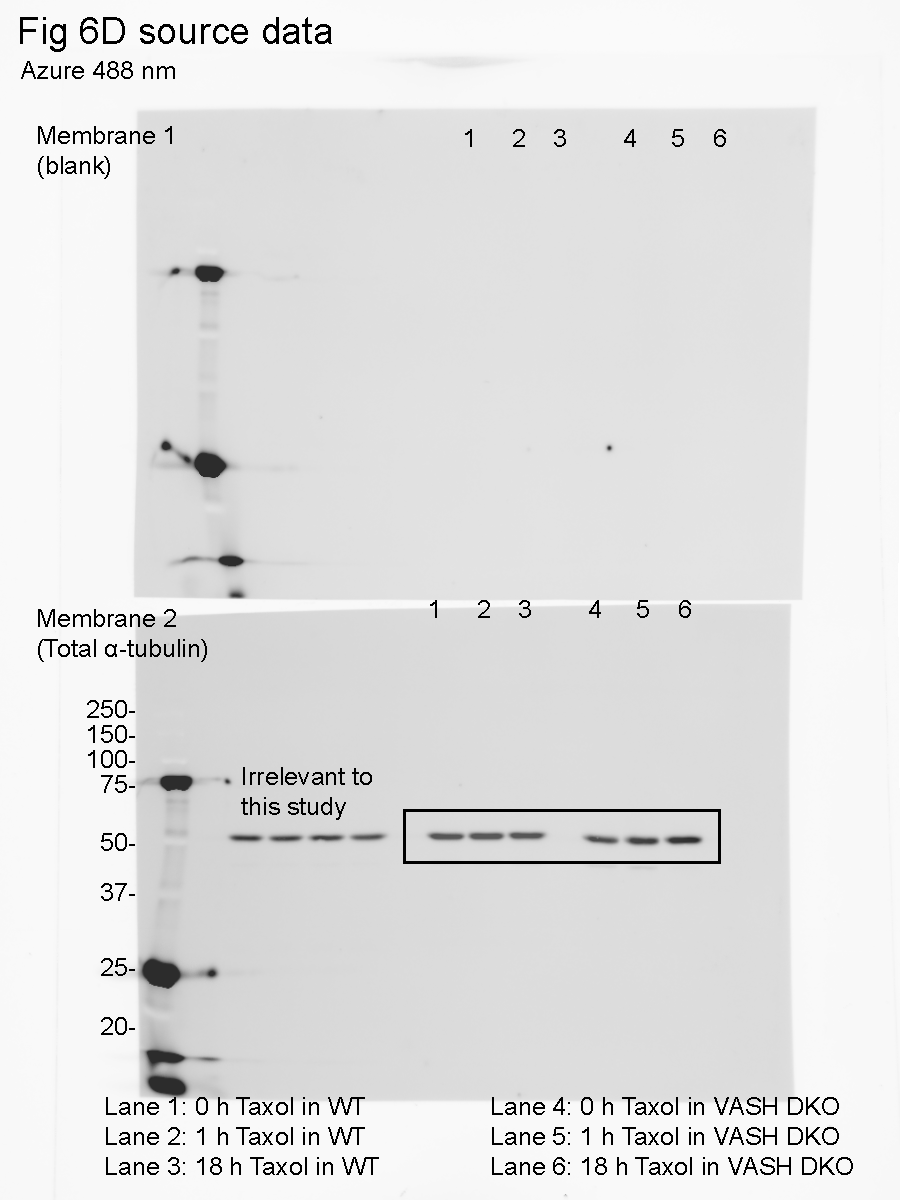

Supplement: Supplementary file 7 — Source data Fig. 6 [file 44318_2026_772_MOESM7_ESM.zip › source data_Figure 6/6D/western_total a-tubulin_labeled.tif]

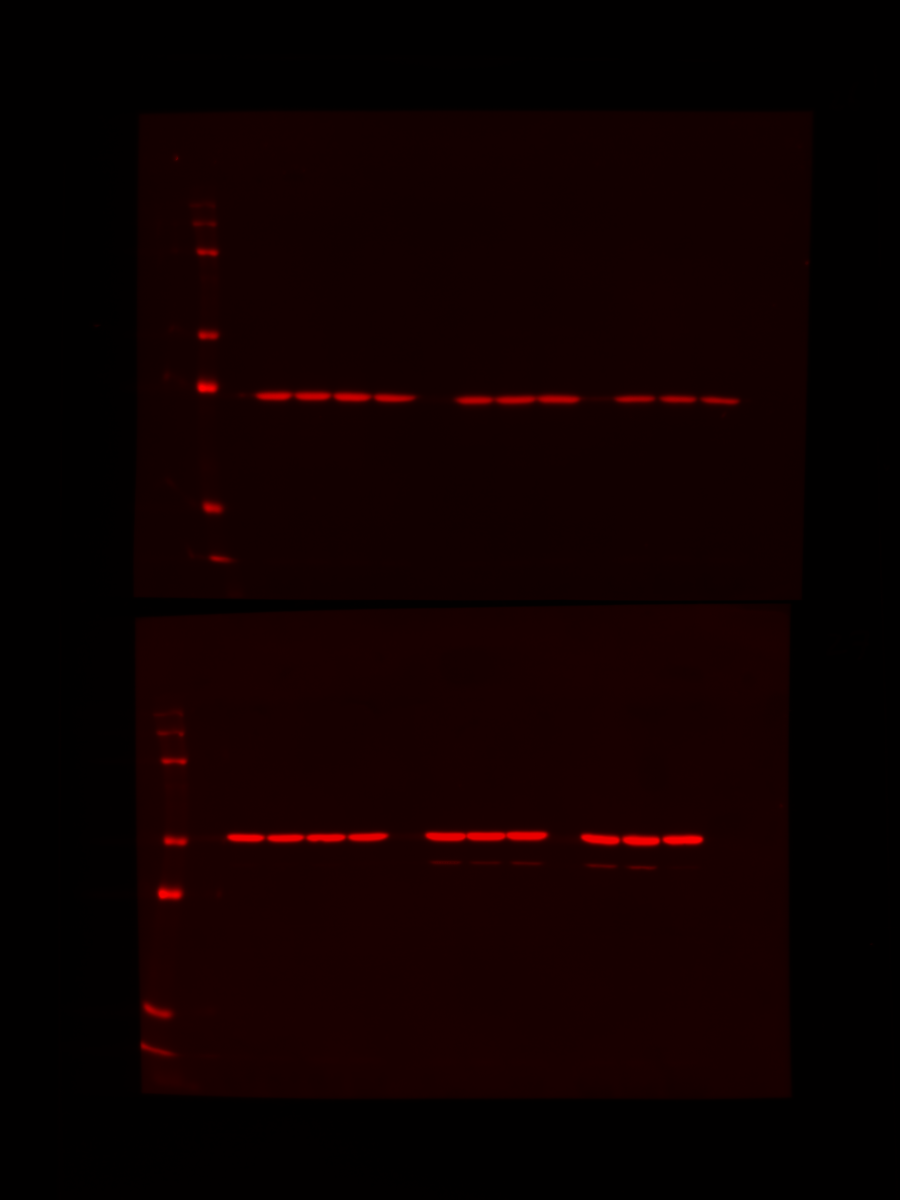

Supplement: Supplementary file 7 — Source data Fig. 6 [file 44318_2026_772_MOESM7_ESM.zip › source data_Figure 6/6D/western_GAPDH_raw.tif]

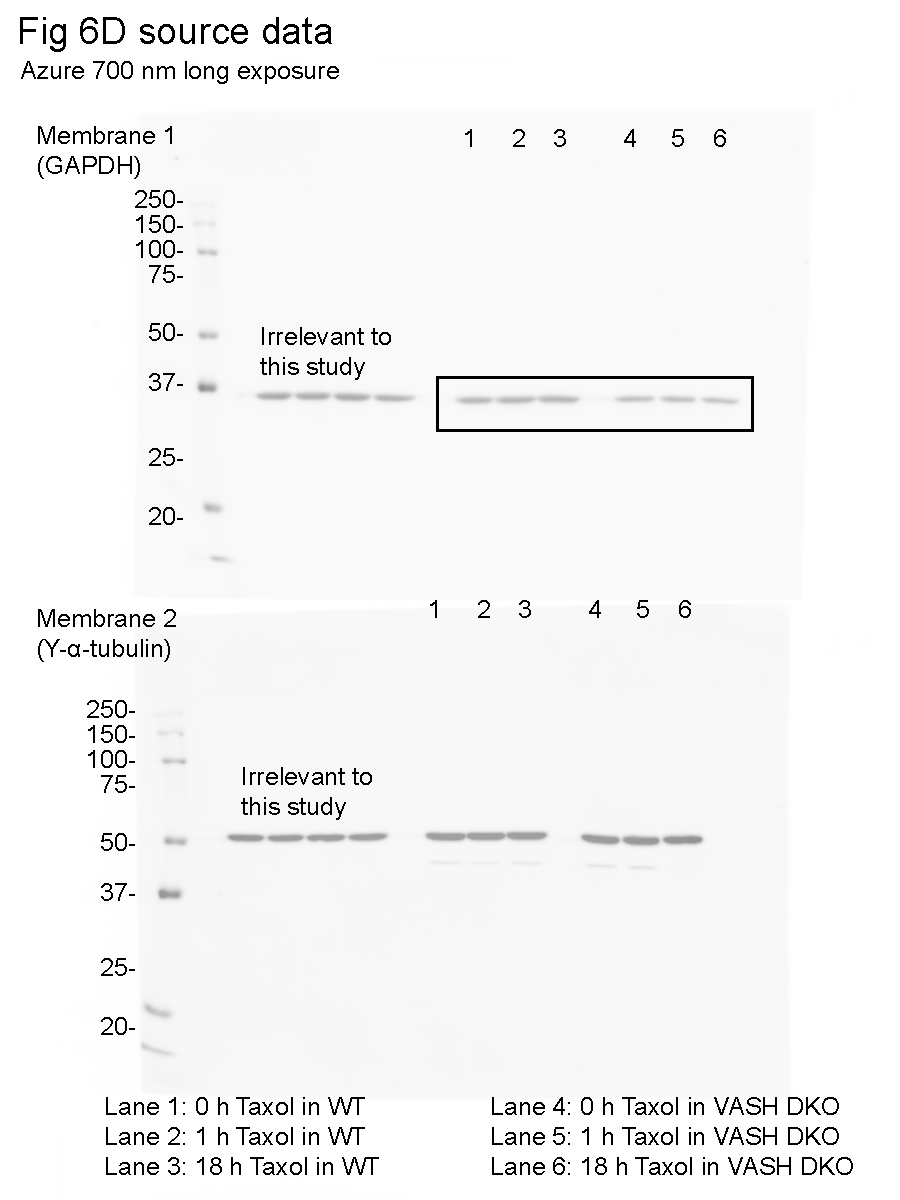

Supplement: Supplementary file 7 — Source data Fig. 6 [file 44318_2026_772_MOESM7_ESM.zip › source data_Figure 6/6D/western_GAPDH_labeled.tif]

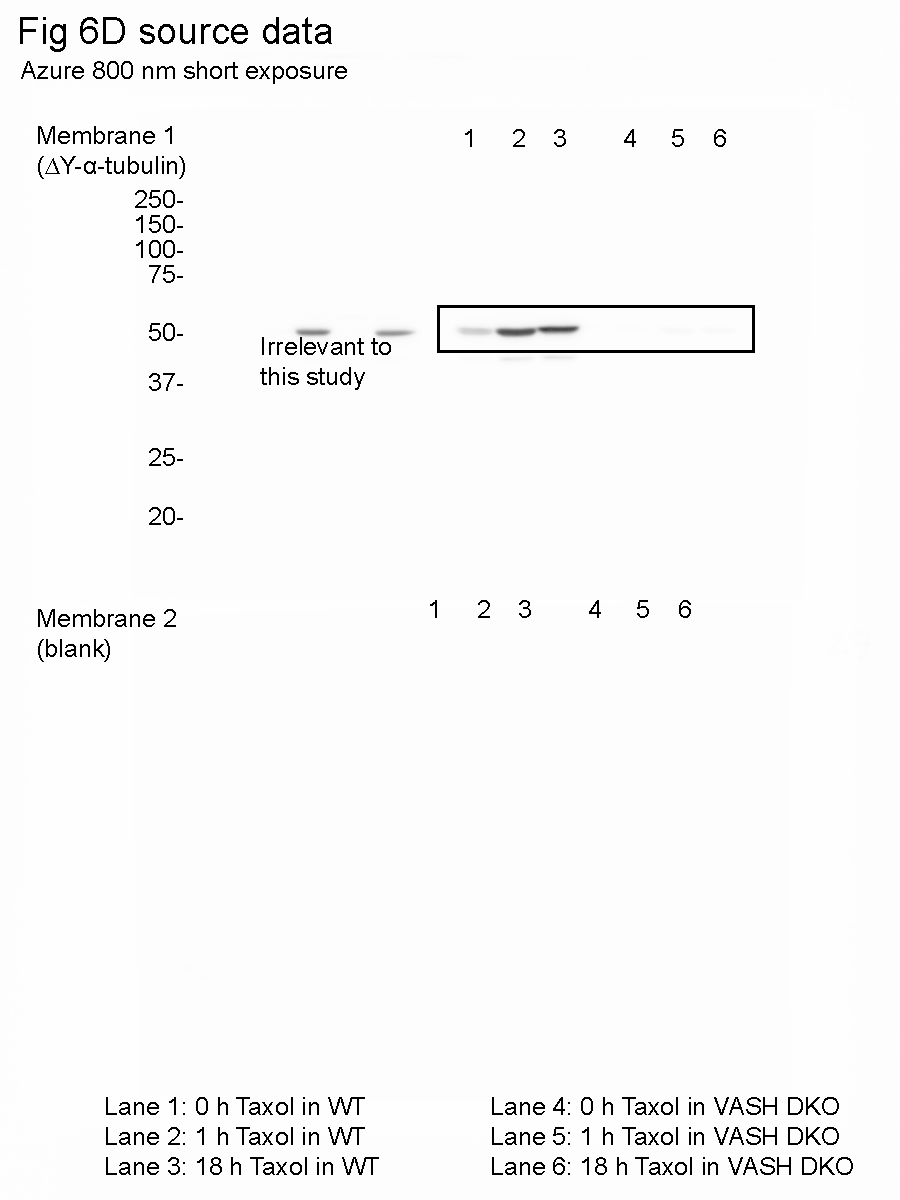

Supplement: Supplementary file 7 — Source data Fig. 6 [file 44318_2026_772_MOESM7_ESM.zip › source data_Figure 6/6D/western_dY-a-tubulin,short exp_labeled.tif]

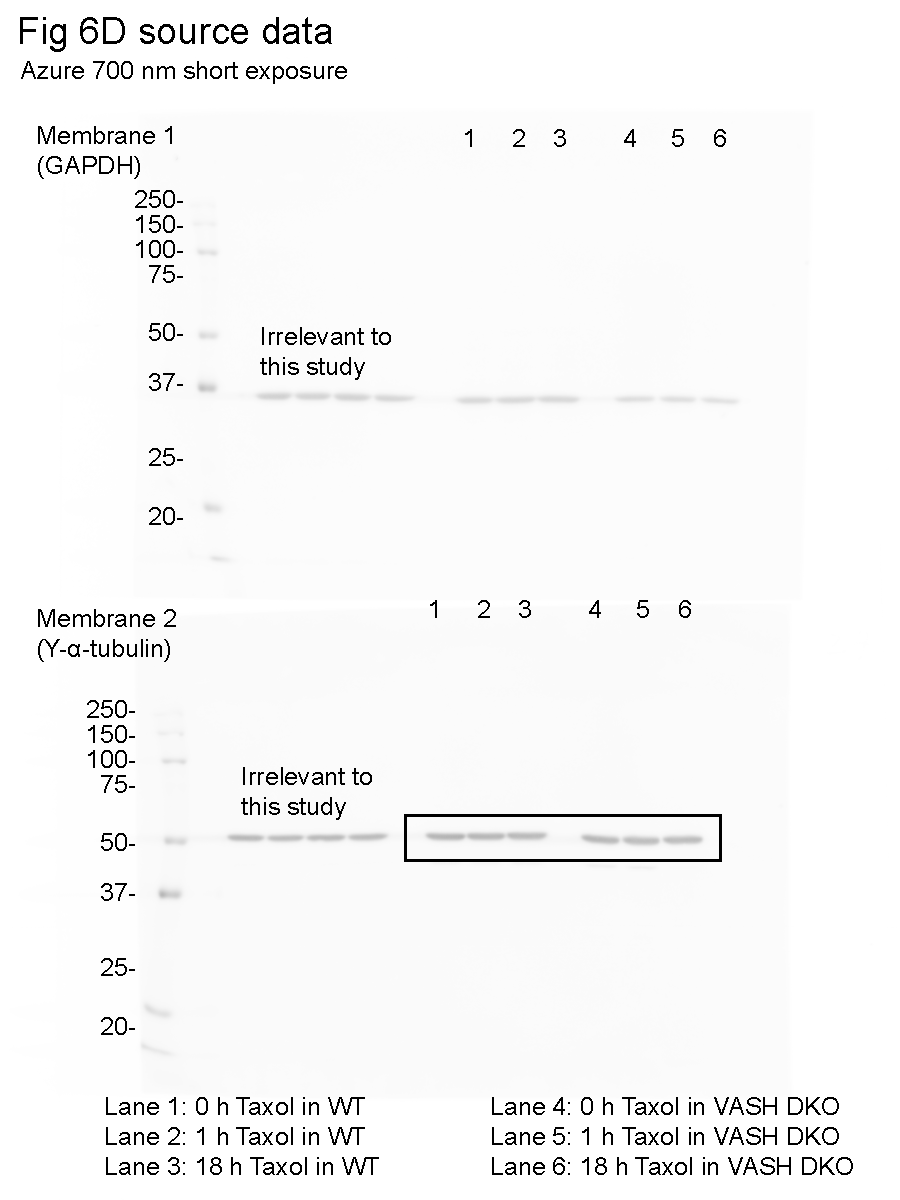

Supplement: Supplementary file 7 — Source data Fig. 6 [file 44318_2026_772_MOESM7_ESM.zip › source data_Figure 6/6D/western_Y-a-tubulin_labeled.tif]

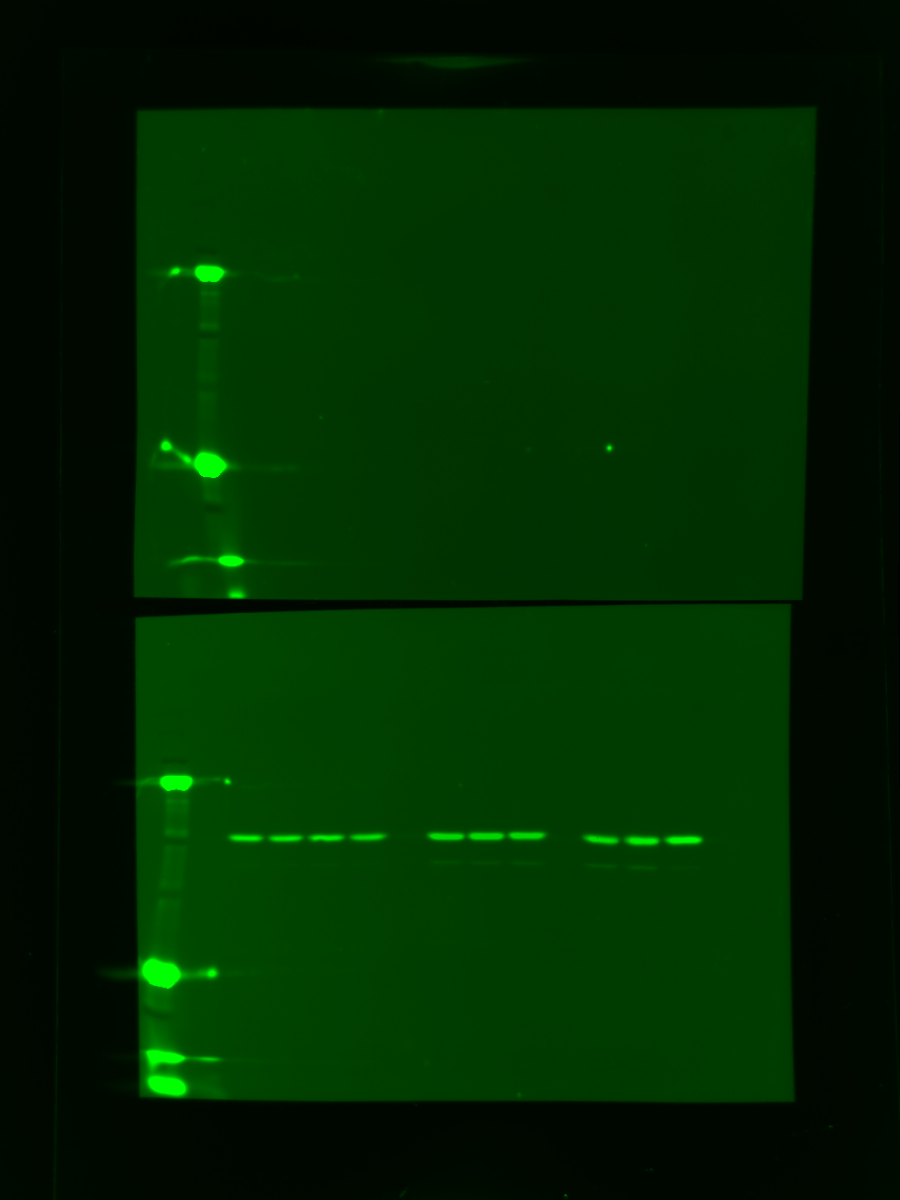

Supplement: Supplementary file 7 — Source data Fig. 6 [file 44318_2026_772_MOESM7_ESM.zip › source data_Figure 6/6D/western_total a-tubulin_raw.tif]

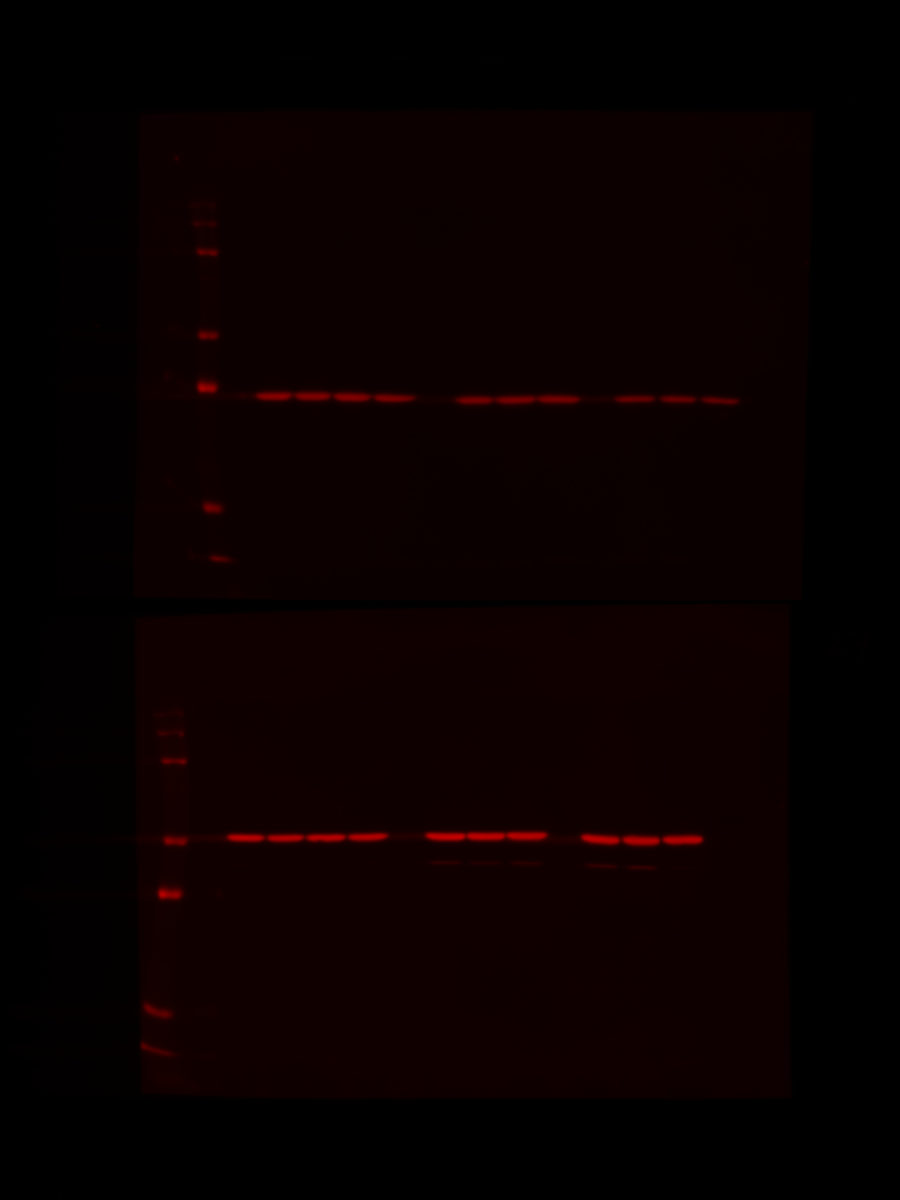

Supplement: Supplementary file 7 — Source data Fig. 6 [file 44318_2026_772_MOESM7_ESM.zip › source data_Figure 6/6D/western_Y-a-tubulin_raw.tif]

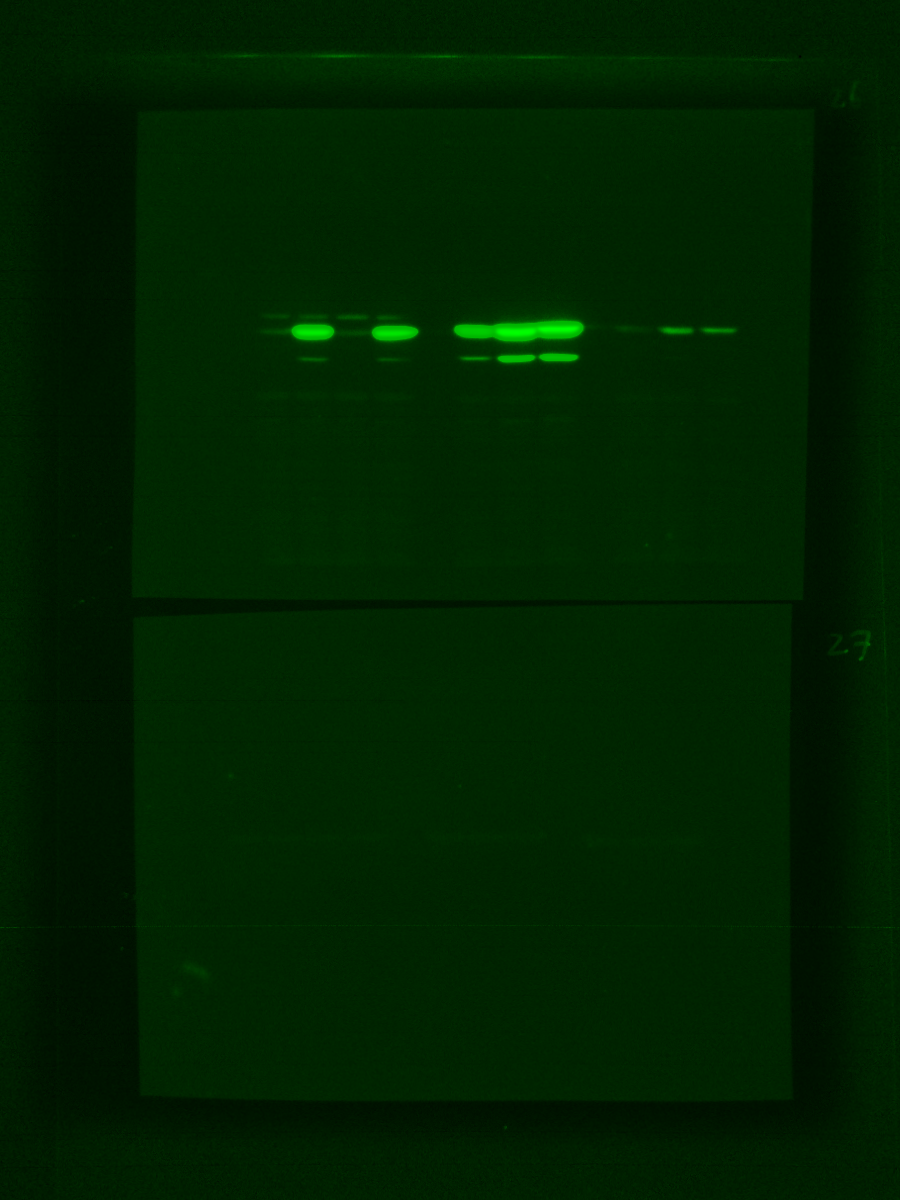

Supplement: Supplementary file 7 — Source data Fig. 6 [file 44318_2026_772_MOESM7_ESM.zip › source data_Figure 6/6D/western_dY-a-tubulin,short exp_raw.tif]
