# Supplementary material for: MATCAP1 preferentially binds an expanded tubulin conformation to generate detyrosinated and ΔC2 α-tubulin
Source: EMBO J. 2026 Apr 13;45(12):4257–82. doi: 10.1038/s44318-026-00772-6 (PMC13269789; doi:10.1038/s44318-026-00772-6)
Supplement: Supplementary file 8 — Expanded View Figures [file 44318_2026_772_MOESM8_ESM.pdf]

# Expanded View Figures

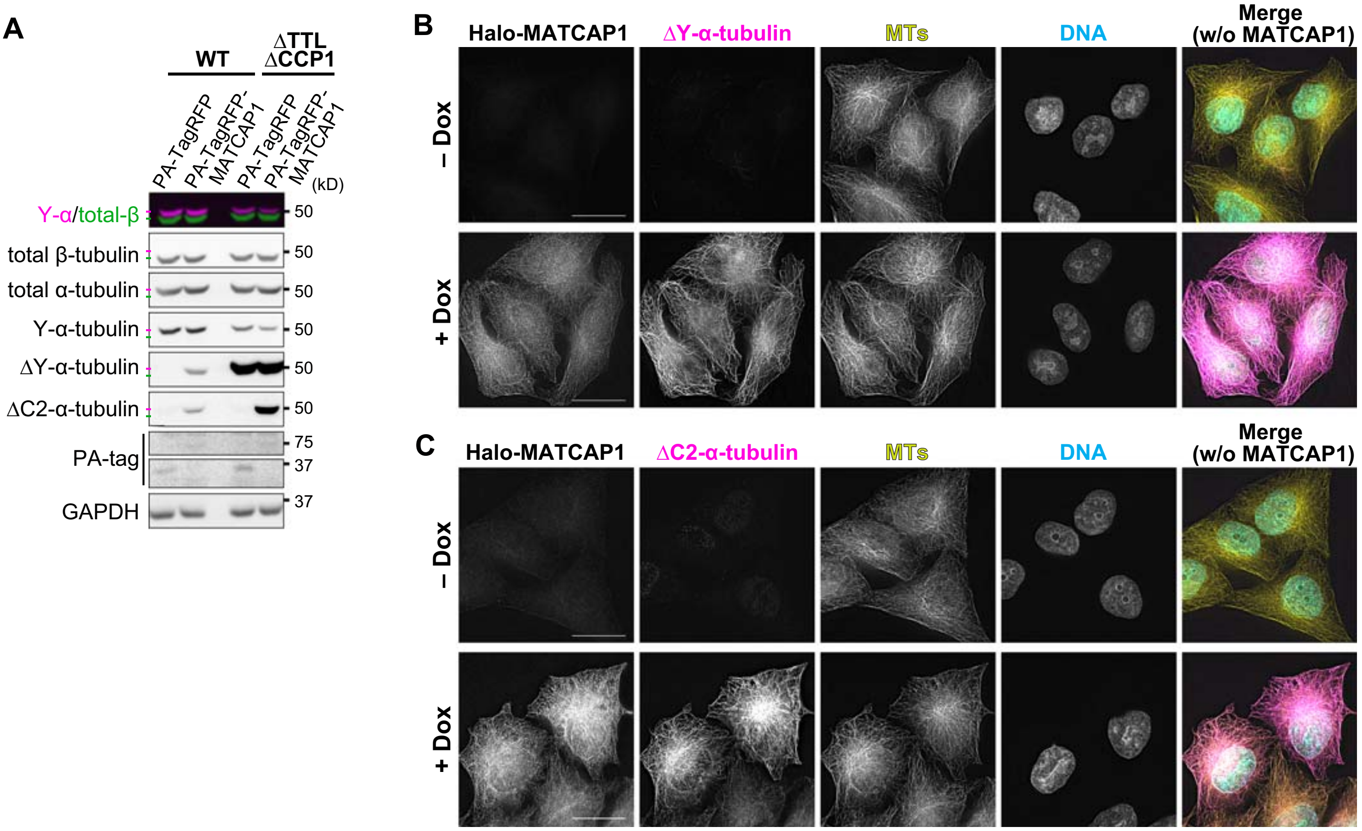


**Figure EV1. MATCAP1 generates both ΔY- and ΔC2-microtubules in cells.**

(**A**) Western blot of cell lysates from WT or Δ*TTL*Δ*CCP1* HeLa cells transiently expressing PA-TagRFP or PA-TagRFP-MATCAP1. The proteins were separated on high-pH gels to resolve α- and β-tubulin, and then the nitrocellulose membranes were blotted with antibodies against tyrosinated α-tubulin (Y-α, magenta) and β-tubulin (green) simultaneously or with antibodies against total α-tubulin, ΔY-α-tubulin, ΔC2-α-tubulin, the PA tag, and GAPDH. (**B**, **C**) Halo-MATCAP1 stable HeLa cells were untreated (-Dox) or treated with doxycycline (+Dox) to induce Halo-MATCAP1 expression. Halo-MATCAP1 was labeled with JFX554 Halo ligand, and then cells were fixed and stained with antibodies against (**B**) ΔY-α-tubulin or (**C**) ΔC2-α-tubulin (magenta) and total α-tubulin (yellow). DNA is shown in cyan. Scale bars, 20 μm.

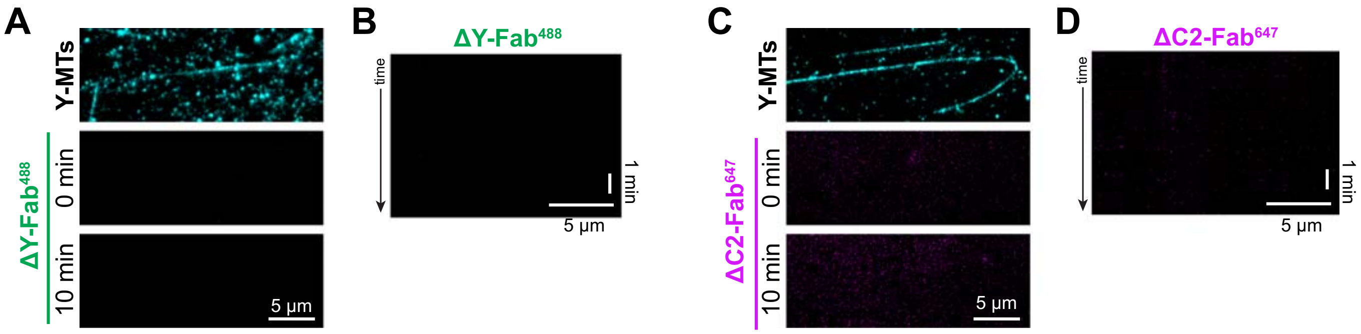


**Figure EV2. Controls using untransfected cell lysates in microscopy-based enzymatic assays.**

(**A**, **B**) Specificity of the ΔY-Fab$^{488}$ probe for microtubules in the presence of untransfected COS-7 cell lysates. (**A**) Representative images of ΔY-Fab$^{488}$ probe labeling of Taxol-stabilized HeLa microtubules (cyan) at 0 min and after 10 min incubation with untransfected COS-7 cell lysates. Scale bar, 5 μm. (**B**) Representative kymographs showing ΔY-Fab$^{488}$ probe labeling of microtubules over time after the addition of untransfected cell lysates. Time is shown on the *y* axis (scale bar, 1 min), and distance along the microtubule is on the *x* axis (scale bar, 5 μm). (**C**, **D**) Specificity of the ΔC2-Fab$^{647}$ probe for microtubules in the presence of untransfected COS-7 cell lysates. (**C**) Representative images of ΔC2-Fab$^{647}$ probe labeling of Taxol-stabilized HeLa microtubules (cyan) at 0 min and after 10 min incubation with untransfected cell lysates. Scale bar, 5 μm. (**D**) Representative kymographs showing ΔC2-Fab$^{647}$ probe labeling of microtubules over time after the addition of untransfected cell lysates. Time is shown on the *y* axis (scale bar, 1 min), and distance along the microtubule is on the *x* axis (scale bar, 5 μm).

 © The Author(s)

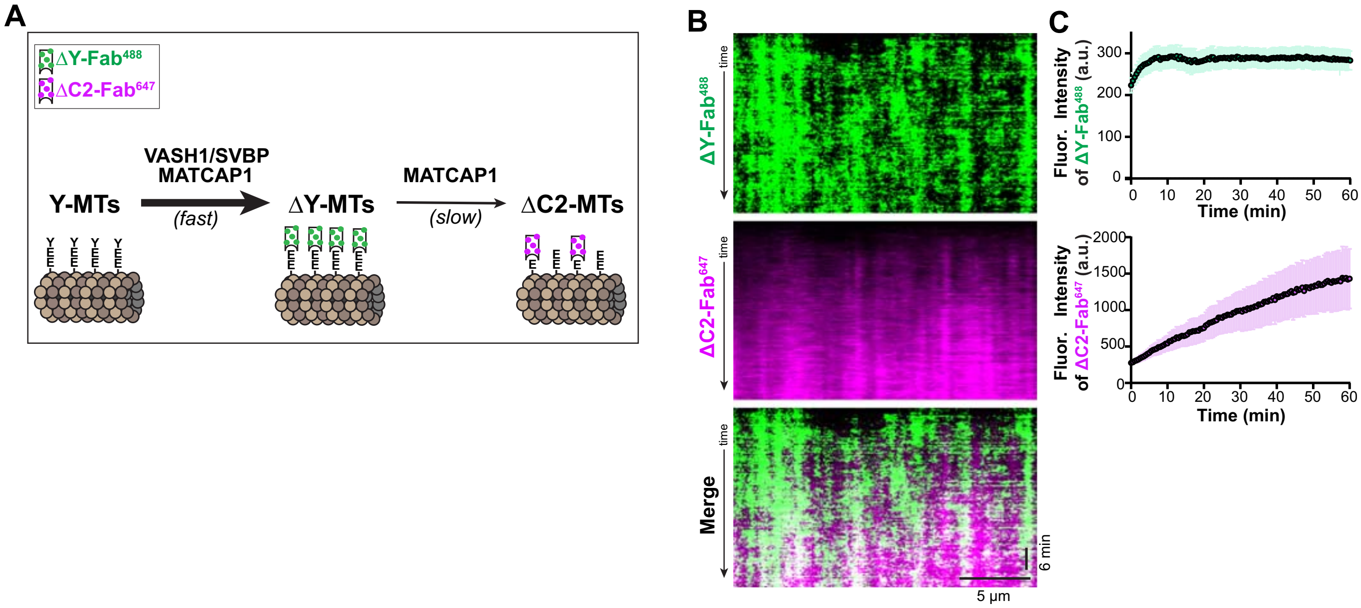


**Figure EV3. MATCAP1 generates ΔY-MTs faster than ΔC2-MTs in vitro.**

(**A**) Schematic illustrating the sequential cleavage of the α-tubulin CTT by MATCAP1 and probe binding. (**B**, **C**) Long imaging time visualizing the generation of the ΔY- and ΔC2-α-tubuilin modifications. (**B**) Representative kymographs showing ΔY-Fab$^{488}$ (green) and ΔC2-Fab$^{647}$ (magenta) labeling of Taxol-stabilized HeLa microtubules over 1 h incubation with 4.2 nM Halo-MATCAP1 in cell lysates. Time is shown on the *y* axis (scale bar, 6 min), and distance along the microtubule is on the *x* axis (scale bar, 5 μm). (**C**) Quantification of the mean fluorescence intensity of ΔY-Fab$^{488}$ (green) and ΔC2-Fab$^{647}$ (magenta) probes along microtubules over time. Data are presented as mean ± SD, with $n = 100$ microtubules from two independent experiments.

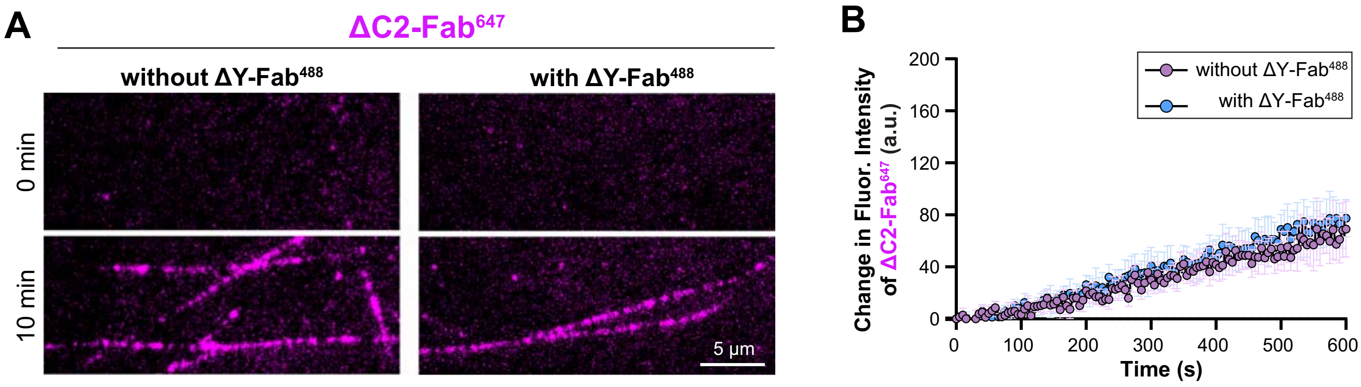


**Figure EV4. The ΔY-Fab does not hinder the binding of the ΔC2-Fab.**

(**A**) Representative images of ΔC2-Fab$^{647}$ labeling of Taxol-stabilized HeLa microtubules at 0 min and after 10 min incubation with 1 nM Halo-MATCAP1 in cell lysates, without or with ΔY-Fab$^{488}$. Scale bar, 5 μm. (**B**) Quantification of the change in fluorescence intensity of ΔC2-Fab$^{647}$ probe labeling along microtubules over time. The data are presented as mean ± SD, with $n = 17$–23 microtubules from two independent experiments.

 © The Author(s)

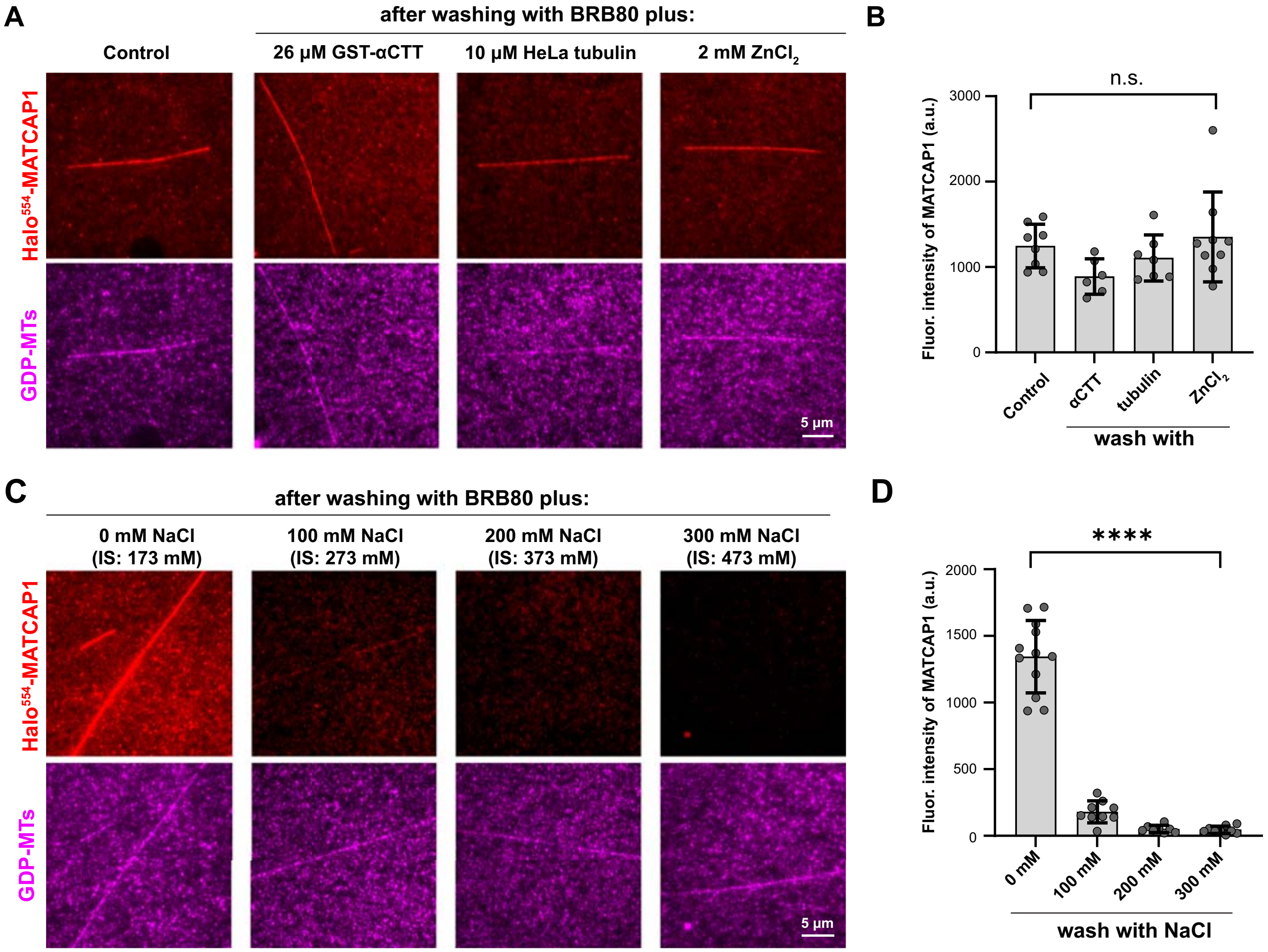


**Figure EV5. MATCAP1 detaches from microtubules in high ionic strength buffer.**

(**A**) Representative images of 1 nM Halo$^{554}$-MATCAP1 (red) in cell lysates bound to glycerol-stabilized HeLa GDP-MTs (magenta) after washing with BRB80 containing 26 µM GST-αCTT, 10 µM HeLa tubulin, or 2 mM $ZnCl_2$. Scale bar, 5 µm. (**B**) Quantification of the mean fluorescence intensity of Halo$^{554}$-MATCAP1 under the indicated conditions is shown in (**A**). Data are presented as mean ± SD, with $n = 6$–9 microtubules. n.s., not significant (one-way ANOVA). (**C**) Representative images of 1 nM Halo$^{554}$-MATCAP1 (red) in cell lysates bound to glycerol-stabilized HeLa GDP-MTs (magenta) after washing with BRB80 containing increasing concentrations of NaCl. IS Ionic strength. Scale bar, 5 µm. (**D**) Quantification of the mean fluorescence intensity of Halo$^{554}$-MATCAP1 under the indicated conditions is shown in (**C**). Data are presented as mean ± SD, with $n = 8$–12 microtubules. ****$P < 0.0001$ (one-way ANOVA).

© The Author(s)

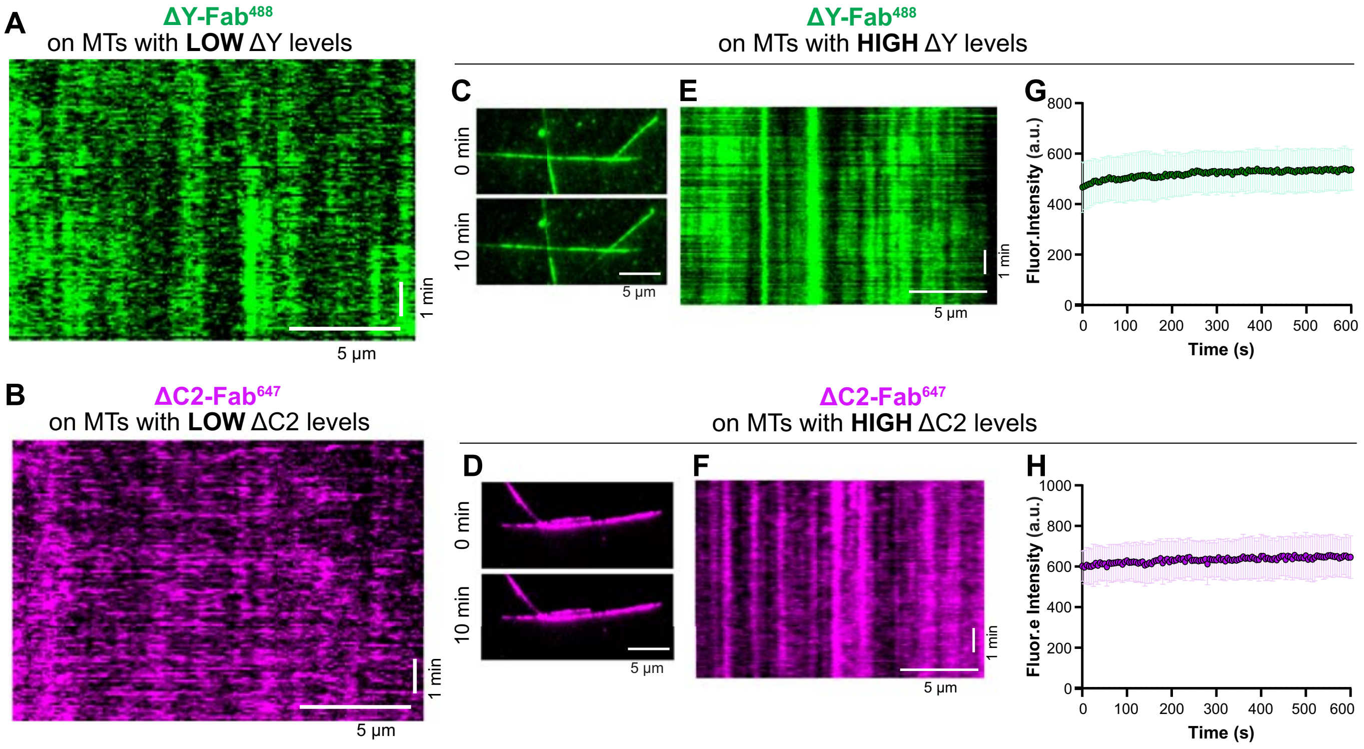


**Figure EV6. Controls for Fab binding to microtubules.**

(**A**, **B**) Single-molecule imaging of probe binding to pre-cleaved microtubules containing low levels of modification. Pre-cleaved microtubules were generated by incubating Taxol-stabilized HeLa microtubules with (**A**) 0.07 nM VASH1/SVBP in cell lysates for 2–3 s or (**B**) 0.7 nM MATCAP1 in cell lysates for 3 min to generate low levels of modification. The enzymes were then washed away with high-salt buffer, and the probes were added to the flow chamber and monitored over time. Representative kymographs are shown for (**A**) ΔY-Fab$^{488}$ or (**B**) ΔC2-Fab$^{647}$ probe labeling. Time is shown on the *y* axis (scale bar, 1 min) and distance along the microtubule is on the *x* axis (scale bar, 5 μm). (**C–H**) Probe binding to pre-cleaved microtubules containing high levels of modification. Pre-modified microtubules were generated by incubation with (**C**, **E**, **G**) 0.7 nM VASH1/SVBP in cell lysates for 2–3 s or (**D**, **F**, **H**) 1.4 nM MATCAP1 in cell lysates for 15 min. The enzymes were then washed away with high-salt buffer, followed by the addition of the Fab probes to the flow chamber. (**C**, **D**) Representative images of (**C**) ΔY-Fab$^{488}$ or (**D**) ΔC2-Fab$^{647}$ probe labeling of pre-cleaved microtubules at 0 min and after 10 min addition of the respective Fab probe. Scale bar, 5 μm. (**E**, **F**) Representative kymographs of (**E**) ΔY-Fab$^{488}$ or (**F**) ΔC2-Fab$^{647}$ probe labeling of pre-cleaved HeLa microtubules over time. Time is shown on the *y* axis (scale bar, 1 min), and distance along the microtubule is on the *x* axis (scale bar, 5 μm). (**G**, **H**) Quantification of the mean fluorescence intensity of (**G**) ΔY-Fab$^{488}$ probe and (**H**) ΔC2-Fab$^{647}$ probe along microtubules. Data are presented as mean ± SD, with $n = 13–21$ microtubules from two independent experiments.

 © The Author(s)

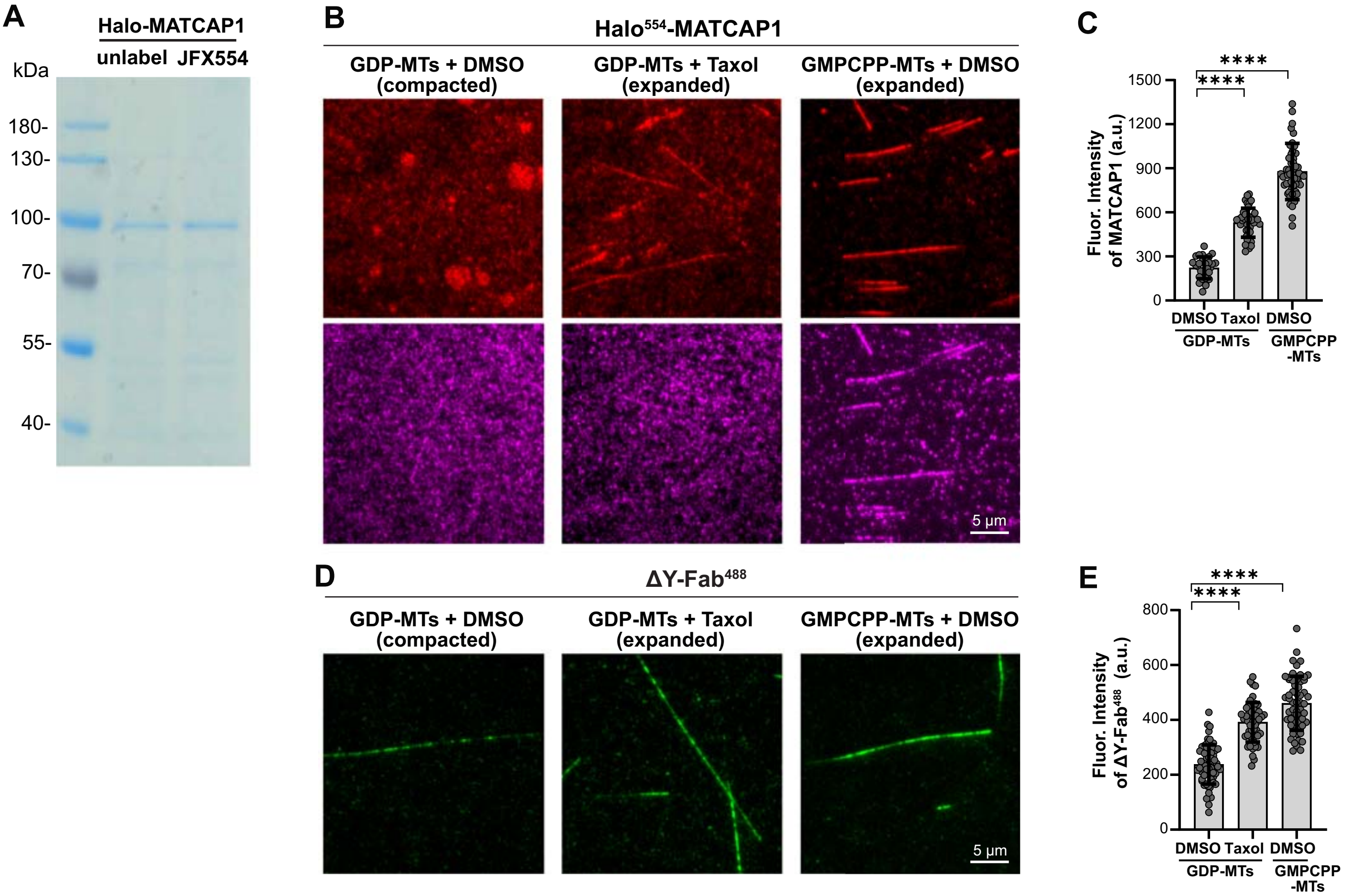


**Figure EV7. The microtubule binding and detyrosination activity of purified MATCAP1 are affected by the conformational state of the microtubule.**

(**A**) Coomassie-stained gel of TwinStrep-Halo-MATCAP1 protein purified from COS-7 cells. (**B**, **C**) Microtubule binding of purified MATCAP1 is regulated by the conformational state of tubulin in the microtubule lattice. (**B**) Representative images of 1.5 nM purified TwinStrep-Halo$^{554}$-MATCAP1 protein binding to glycerol-stabilized GDP-MTs with DMSO, GDP-MTs with Taxol, or GMPCPP-MTs with DMSO. Scale bar, 5 µm. (**C**) Quantification of the mean fluorescence intensity of TwinStrep-Halo$^{554}$-MATCAP1 along the microtubules in (**B**). Each point represents the mean fluorescence intensity of an individual microtubule. Data are presented as mean ± SD, with $n = 30$–56 microtubules from two or three independent experiments. $P = 1.893E-22$ (GDP-MTs, DMSO vs. GDP-MTs, Taxol). $P = 1.330E-30$ (GDP-MTs, DMSO vs. GMPCPP-MTs, DMSO). ****$P < 0.0001$ (two-tailed, *t* test). (**D**, **E**) Detyrosination activity of purified MATCAP1 is regulated by the conformational state of tubulin in the microtubule lattice. (**D**) Representative images of ΔY-Fab$^{488}$ labeling of glycerol-stabilized GDP-MTs with DMSO, GDP-MTs with Taxol, or GMPCPP-MTs with DMSO after incubation with 1.5 nM purified Halo-MATCAP1 protein. MATCAP1 was removed by a high-salt wash step before the addition of the ΔY-Fab$^{488}$ probe. Scale bar, 5 µm. (**E**) Quantification of the mean fluorescence intensity of the ΔY-Fab$^{48}$ probe labeling along the microtubules in (**D**). Each point represents the mean fluorescence intensity of an individual microtubule. Data are presented as mean ± SD, with $n = 52$–65 microtubules from two or three independent experiments. $P = 7.098E-21$ (GDP-MTs, DMSO vs. GDP-MTs, Taxol). $P = 2.317E-28$ (GDP-MTs, DMSO vs. GMPCPP-MTs, DMSO). ****$P < 0.0001$ (two-tailed, *t* test).

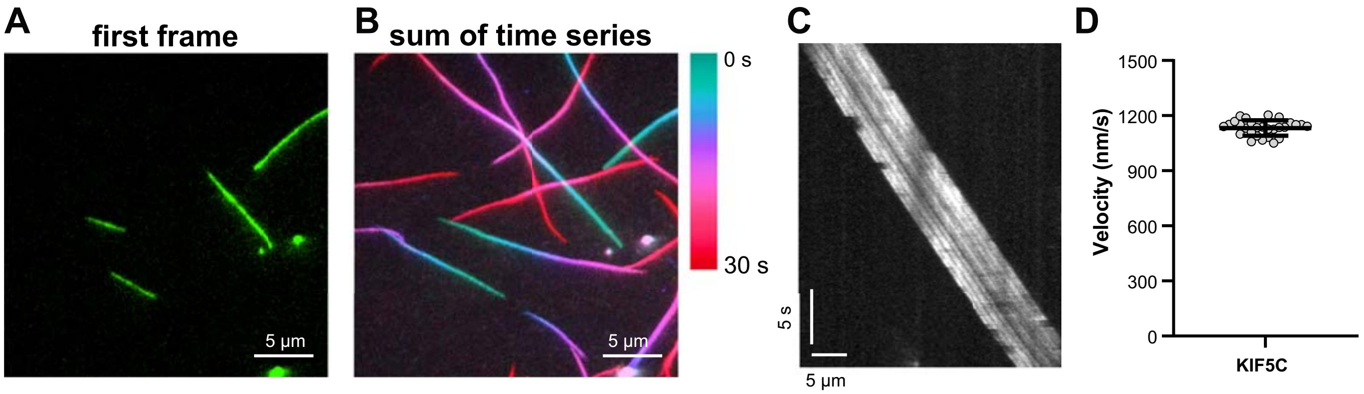


**Figure EV8. Motility properties of purified kinesin-1 protein in microtubule gliding assays.**

(**A**, **B**) Representative images. 200 nM unlabeled purified kinesin-1 protein was attached to the flow cells, and then Taxol-stabilized microtubules were introduced in the presence of ATP, and images were acquired over time. (**A**) First frame of imaging. (**B**) Time-lapse projection. Color bar, imaging time. Scale bars, 5 μm. (**C**) Representative kymograph of an individual microtubule gliding over time. Time is shown on the *y* axis (scale bar, 5 s), and distance along the microtubule is on the *x* axis (scale bar, 5 μm). (**D**) Quantification of the velocity of microtubule gliding driven by purified kinesin-1 protein. Data are presented as mean ± SD, with $n = 28$ microtubules.

 © The Author(s)
