# Supplementary material for: MATCAP1 preferentially binds an expanded tubulin conformation to generate detyrosinated and ΔC2 α-tubulin
Source: EMBO J. 2026 Apr 13;45(12):4257–82. doi: 10.1038/s44318-026-00772-6 (PMC13269789; doi:10.1038/s44318-026-00772-6)
Supplement: Supplementary file 1 — Peer Review File [file 44318_2026_772_MOESM1_ESM.pdf]

MATCAP1 preferentially binds an expanded tubulin conformation to generate detyrosinated and ΔC2 α-tubulin

Yang Yue, Takashi Hotta, Ryoma Ohi, and Kristen Verhey

Corresponding authors: Kristen Verhey (kjverhey@umich.edu) , Ryoma Ohi (oryoma@umich.edu)

Review Timeline:

Transferred from Review Commons:	29th Dec 25
Editorial Decision:	27th Feb 26
Revision Received:	12th Mar 26
Accepted:	26th Mar 26

Editor: Ieva Gailite

Transaction Report:

This manuscript was transferred to The EMBO Journal following peer review at Review Commons.

(Note: With the exception of the correction of typographical or spelling errors that could be a source of ambiguity, letters and reports are not edited. Depending on transfer agreements, referee reports obtained elsewhere may or may not be included in this compilation. Referee reports are anonymous unless the Referee chooses to sign their reports.)

Review #1

1. Evidence, reproducibility and clarity:

Evidence, reproducibility and clarity (Required)

Summary

This paper presents data evaluating the microtubule binding and activity of MAPCAP1, a metallopeptidase that cleaves the C-terminus of alpha tubulin when incorporated into microtubules (MT), and that both binding and activity are dependent on the expanded vs compacted state of the MT lattice. Known modifications of the terminus are removal of the terminal tyrosine (ΔY), or the terminal two residues (ΔC2), and authors show that MATCAP1 can perform both of these modifications, unlike the other known carboxypeptidase, VASH(1/2) / SVBP (hereafter: VASH), which only produces ΔY, and which was evaluated in a previous publication by the same authors. Authors use studies of cells (HeLa and engineered HeLa), as well as invitro studies with purified proteins, to show that MATCAP1 preferentially binds to the expanded MT lattice with long dwell time, and producing both ΔY and ΔC2, in contrast to VASH, which binds to both expanded and compacted lattices, binds with short dwell time and produces only ΔY.

Major comments

In general, the work is well thought out and well done. The context and background are clearly spelled out and well referenced. It is well-presented and doesn't go beyond the data (but see small point below). The methods are remarkably detailed and would be easy to follow, if one had the reagents that authors do.

Minor comments

Prior studies are well referenced. The text and figures are well composed and easy to follow.
.
A small point relates to Figure 6D, in which the production of ΔY is evaluated from MT from HeLa cells treated with DMSO or Taxol, in the absence or presence of EpoY, a VASH1/SVBP inhibitor. The point of the figure is that EpoY doesn't prevent all ΔY formation, therefore there must be another enzyme (i.e. MATCAP1) present to perform this. The problem is that it isn't clear, at first glance, that EpoY did anything. Closer inspection shows that the band for ΔY is dimmer in the lane with EpoY, but I missed it on first read. A further sample with cells lacking MATCAP1 would show that in that case the EpoY should knock out the ΔY completely.
I estimate that this will take less than one month to accomplish, if authors have a ΔMATCAP1 cell line.

2. Significance:

Significance (Required)

This paper is very well done - it clearly lays out the relevant questions and the progress of experiments, and then presents them clearly and interprets them clearly and in context.

It will be of interest to those who study MT structure, regulation, and post-translational modifications (PTM), such as myself. It will also be of interest to those who are concerned with the broader point of how enzyme activity is regulated by structural details of binding surfaces.

3. How much time do you estimate the authors will need to complete the suggested revisions:

Estimated time to Complete Revisions (Required)

(Decision Recommendation)

Less than 1 month

4. *Review Commons* values the work of reviewers and encourages them to get credit for their work. Select 'Yes' below to register your reviewing activity at Web of Science Reviewer Recognition Service (formerly Publons); note that the content of your review will not be visible on Web of Science.

Web of Science Reviewer Recognition

No

Review #2

1. Evidence, reproducibility and clarity:

Evidence, reproducibility and clarity (Required)

The manuscript by Yue et al. investigates the molecular mechanisms by which MATCAP1, a metalloprotease enzyme, selectively modifies microtubules through detyrosination and ΔC2 modifications of α-tubulin. Using sophisticated microscopy-based enzymatic assays with newly developed fluorescent Fab probes specific for ΔY- and ΔC2-tubulin modifications, the authors

demonstrate that MATCAP1 preferentially binds to microtubules in an expanded conformational state, achieved through preventing β-tubulin GTP hydrolysis (GMPCPP), Taxol treatment, or active kinesin-1 stepping, and exhibits remarkably long dwell times (>30 seconds) on these substrates. Through real-time visualization experiments in both cellular and reconstituted in vitro systems, they show that MATCAP1 sequentially removes amino acids from the α-tubulin C-terminal tail, generating ΔY modifications faster than ΔC2 modifications, and can operate independently of cytosolic carboxypeptidases. The findings significantly advance our understanding of tubulin post-translational modification regulation by revealing that microtubule lattice conformation serves as a critical gating mechanism for enzymatic activity. This work fits into the broader "tubulin code" framework, where specific combinations of post-translational modifications create functionally distinct microtubule populations that selectively recruit different motor proteins and microtubule-associated proteins. The discovery that MATCAP1's activity is conformationally gated provides a molecular explanation for how cells might spatially and temporally control tubulin modifications, complementing recent structural studies and adding to the growing understanding of how mechanical and conformational states of the cytoskeleton influence biochemical processes.
The paper should be of high interest to the cytoskeleton and cell biology communities and I support publication after the authors address a few concerns below.

Major points:

1. There is no explicit demonstration that the Fab probes do not bind to microtubules in the absence of MATCAP1 or VASH1/SVBP. It is critical, particularly for the ΔC2 Fab647 probe, to show that incubation for 10 minutes with the Fab but without added modifying enzyme results in negligible labeling. This would rule out two key confounders: (a) pre-existing ΔY or ΔC2 PTMs in the tubulin preparation, and (b) non-specific Fab probe binding.
2. Figure 3: The non-saturation of ΔC2-Fab647 signal over time suggests potential issues with probe specificity and/or Fab probe affinity. Controls in which the ΔC2 probe is incubated with microtubules lacking any enzyme treatment should be shown, ideally on the same batch/preparation of tubulin as used in experimental samples. The specificity and occupancy properties of these probes (especially ΔC2-Fab647) should be clarified.
3. It is unclear when and why the authors add MATCAP1 or VASH1/SVBP enzymes as cell lysates rather than as purified proteins. This raises some concerns
- Lysates can contain endogenous enzymes, nucleases, or additional PTM-modifying activities that may confound the interpretation of results. It would be more rigorous to demonstrate key findings with purified MATCAP1 and VASH1/SVBP proteins, at least as a confirmatory step.
- The justification for using lysates rather than purified proteins should be clearly stated in the Results and rationales provided in the Methods. If purified proteins were limiting, this should be acknowledged, and the purity and concentration of MATCAP1 in lysates should be quantified and controlled across experiments.
4. The major conclusions of the paper rest on rate measurements for fluorescently labeled MATCAP1 binding to microtubules with different PTM states. However, inspection of the kymographs shows that most particles appear stably bound before imaging starts, leading to some confusion. The authors must clarify in both the Results and Methods whether only particles landing de novo during image acquisition were counted when calculating landing rates. If so, the precise criteria used to define a "landing event" versus pre-bound molecules should be detailed.

Given the use of cell lysates with fluorophore-conjugated Halo ligand, a negative control is essential: add Halo ligand to lysates from cells never transfected with (or expressing) MATCAP1, then perform the same TIRF binding and landing analysis. This would control for non-specific labeling of cellular proteins and verify that any observed binding is genuinely due to fluorescent MATCAP1, not other endogenous or labeled species. Without this clarification, it is difficult to interpret the main results of lattice preference as measured by landing rates, although some of this concern is alleviated by average fluorescence measurements as in Fig. 5.

Minor comments:

1. Figure S4 examines MATCAP1 detachment from microtubules in response to buffer changes, but critical technical details are missing. The basis for calculating ionic strength (IS) across the different buffer conditions should be documented, including the formula used and all constituent ions considered. The abbreviation "IS" is not defined in the figure or legend and should be added.
2. The interpretation that ATP-bound KIF5C can promote an expanded microtubule lattice, enhancing MATCAP1 binding, is important to the authors' conclusions. Yet, there are no data presented to demonstrate that the purified KIF5C used is functionally active as a motor. The manuscript should report a direct activity assay showing that the KIF5C preparation is capable of ATP-driven stepping along microtubules, e.g., via gliding or single-molecule motility assays. If not performed, a statement should be added to justify the assumption of activity.

2. Significance:

Significance (Required)

This is a nice paper that should be of interest to a broad community of cell biologists and biochemists interested in the cytoskeleton and tubulin code. The reagents and assays developed will also be of interest to this community.

3. How much time do you estimate the authors will need to complete the suggested revisions:

Estimated time to Complete Revisions (Required)

(Decision Recommendation)

Between 3 and 6 months

4. *Review Commons* values the work of reviewers and encourages them to get credit for their work. Select 'Yes' below to register your reviewing activity at [Web of Science]

Reviewer Recognition Service (formerly Publons); note that the content of your review will not be visible on Web of Science.

Web of Science Reviewer Recognition

Yes

Review #3

1. Evidence, reproducibility and clarity:

Evidence, reproducibility and clarity (Required)

This manuscript examines the mechanism of MATCAP1, a tubulin-modifying enzyme that removes residues from the C-terminal tails. The authors find that MATCAP1 generates both dY and dC2 tubulin using a combination of cell-based assays, in vitro assays with cell lysates, and in vitro assays with purified proteins. They find that MATCAP1 acts preferentially on microtubules in the expanded conformation, which is consistent with this team's recently-published work on VASH/SVBP, a tubulin-modifying assay that generated dY tubulin, suggesting a general mechanism for regulation of the dY and dC2 modifications. Overall, the manuscript is excellent, with high-quality data that strongly supports the central conclusions.

Concerns / Areas for Improvement

- The paper discusses the kinetics of MATCAP enzymatic activity in a few places. For example, in Figure 2, the authors show that the binding of their dY probe to microtubules is relatively fast (Fig. 2D), while the binding of their dC2 probe to microtubules is relatively slow (Fig. 2I). One obvious interpretation of this data is that the reaction is sequential: MATCAP first cleaves the Y (quickly) and then cleaves the E to generate dC2 (slowly). The Abstract and Introduction use the phrase "sequentially", although the authors do not make an explicit link between their data in Figure 2 and the concept of sequential cleavage. This is a minor writing issue. A more significant issue is how the data in Figure 2 interacts with the data in Figure 4. In Figure 4F, the binding of the dC2 probe is identical regardless of whether the microtubules were Y or dY to start with. I'm confused by this finding. If the microtubules are Y-MTs, then they need to first be cleaved to dY, then cleaved to dC2, and then the probe can bind. The data in Figure 4F can be explained a few ways. Option 1 is that the cleavage of the Y to generate the dY microtubules is really really really fast, such that it's not detectable in the binding kinetics of the dC2 probe. But this option is not consistent with Figure 2D and Figure 3D, which shows that the dY probe takes 10's to 100's of seconds to reach saturation. In other words, cleavage of the Y is fast, but it's not THAT fast. Option 2 to explain Figure 4F could be that the binding of the dC2 probe is actually rather slow, such that the kinetics of dC2 probe binding are a measure only of the probe's binding kinetics,

not the rate of generation of the epitope. However, if that's the case, then the data in Figure 2D and 3D is not sufficient to demonstrate the "MATCAP1 generates dY-MTs faster than dC2-MTs in vitro", because that conclusion interprets the slow binding of the dC2 probe as evidence of slow generation of epitope. In Figure S2, the authors measure the binding kinetics of the probes to pre-cleaved MTs. Indeed, the binding of the dC2 probe does appear slower than binding of the dY probe -- but also that the dC2 probe never reaches saturation, even after 1 hour of incubation.

I hope the paragraph above makes sense -- it's hard to describe kinetics w/out just writing down a reaction scheme. But I do believe there is an issue to resolve. Either the dC2 probe is very slow and can't reach saturation for some reason--in which case the title of Figure 3 is not support. Alternatively, the binding of the probe is not very slow, in which case Figure 4F doesn't make sense to me. Perhaps the authors would benefit from writing down the kinetic scheme for a sequential cleavage reaction, including probe binding. The curves they have generated (e.g. in Figure S2B) might then be sufficient to see what's going on.
- Some experiments in the paper are performed with MATCAP1 in cell lysates while other experiments used purified proteins. The rationale for which experiments used which system were unclear to me. At key points, the switch to purified protein was described as a way to exclude potential artifacts that come from the lysate (e.g. another MAP that is present, etc). But why is that a concern in some experiments but not others? Maybe the lysate is actually advantageous? The paper would benefit from a clearer rationale and explanation for the switch between the different systems.
- Similarly, the authors use a catalytically-inactive MATCAP late in the paper (Figure 5)--could the E281Q construct be used elsewhere to measure, e.g., in the experiments using Y vs. dY-MTs? This suggestion is not essential but might strengthen the idea that the Y-status of the lattice is not important to recruitment.
- In Figure 5D, the results show that MATCAP binding to GMPCPP microtubules is higher relative to Taxol-stabilized microtubules, even though (presumably) both microtubules are fully expanded. Does this finding suggest there are other structural features besides expansion that are at work? What could those features be?

2. Significance:

Significance (Required)

Overall, only the first of the concerns above is important relative to the experimental support for the authors' conclusions, namely the conclusion stated in the title of Figure 3. Otherwise, it's an outstanding paper that will make an important contribution to our understanding of how the tubulin code is regulated.

3. How much time do you estimate the authors will need to complete the suggested revisions:

Estimated time to Complete Revisions (Required)

(Decision Recommendation)

Between 1 and 3 months

4. *Review Commons* values the work of reviewers and encourages them to get credit for their work. Select 'Yes' below to register your reviewing activity at Web of Science Reviewer Recognition Service (formerly Publons); note that the content of your review will not be visible on Web of Science.

Web of Science Reviewer Recognition

Yes

We thank the reviewers for their time and effort in reviewing our manuscript and for their many helpful suggestions for improving the strength of our conclusions as well as the presentation of the data. Below we provide a point-by-point response to the reviewers comments with our response in blue text. Changes to the manuscript text are also indicated by blue text.

Reviewer #1

Evidence, reproducibility and clarity

Summary
This paper presents data evaluating the microtubule binding and activity of MAPCAP1, a metallopeptidase that cleaves the C-terminus of alpha tubulin when incorporated into microtubules (MT), and that both binding and activity are dependent on the expanded vs compacted state of the MT lattice. Known modifications of the terminus are removal of the terminal tyrosine (ΔY), or the terminal two residues (ΔC2), and authors show that MATCAP1 can perform both of these modifications, unlike the other known carboxypeptidase, VASH(1/2) / SVBP (hereafter: VASH), which only produces ΔY, and which was evaluated in a previous publication by the same authors. Authors use studies of cells (HeLa and engineered HeLa), as well as invitro studies with purified proteins, to show that MATCAP1 preferentially binds to the expanded MT lattice with long dwell time, and producing both ΔY and ΔC2, in contrast to VASH, which binds to both expanded and compacted lattices, binds with short dwell time and produces only ΔY.

Major comments
In general, the work is well thought out and well done. The context and background are clearly spelled out and well referenced. It is well-presented and doesn't go beyond the data (but see small point below). The methods are remarkably detailed and would be easy to follow, if one had the reagents that authors do.

Response: We thank the reviewer for these positive comments.

Minor comments
Prior studies are well referenced. The text and figures are well composed and easy to follow.

A small point relates to Figure 6D, in which the production of ΔY is evaluated from MT from HeLa cells treated with DMSO or Taxol, in the absence or presence of EpoY, a VASH1/SVBP inhibitor. The point of the figure is that EpoY doesn't prevent all ΔY formation, therefore there must be another enzyme (i.e. MATCAP1) present to perform this. The problem is that it isn't clear, at first glance, that EpoY did anything. Closer inspection shows that the band for ΔY is dimmer in the lane with EpoY, but I missed it on first read. A further sample with cells lacking MATCAP1 would show that in that case the EpoY should knock out the ΔY completely. I

estimate that this will take less than one month to accomplish, if authors have a ∆MATCAP1 cell line.

Response: The reviewer raises an important point concerning the activity of EpoY. As we currently do not have cell lines lacking MATCAP1, we considered an alternative approach to address the reviewer's concern. Instead of EpoY to abolish the activity of VASH1 and VASH2, we used CHL-1 cells in which the expression of both VASH1 and VASH2 has been eliminated [ΔVASH1/2 double knockout (DKO) cells]. Although detyrosination activity was dramatically decreased in the ΔVASH1/2 DKO cells, a small but observable increase in ∆Y-microtubules was observed after treatment with Taxol for 1 and 18 h (**new Figure 6D**). This result indicates that microtubule expansion mediated by Taxol treatment regulates the detyrosination activity of MATCAP1.

This result is consistent with previous work showing that the VASH enzymes are major contributors to the detyrosination of microtubules in CHL-1 cells (Niewenhuis et al 2017). This result is also consistent with previous work showing that Taxol treatment causes a small by measurable increase in the levels of ∆Y-microtubules in ΔVASH1/2 double knockout cells but not in ΔVASH1/2 and ΔMATCAP1 triple knockout cells (Landskron et al., 2022).

In the revised manuscript, we have included the new data, rewritten the Results section, and added supporting references.

Significance

This paper is very well done - it clearly lays out the relevant questions and the progress of experiments, and then presents them clearly and interprets them clearly and in context.

It will be of interest to those who study MT structure, regulation, and post-translational modifications (PTM), such as myself. It will also be of interest to those who are concerned with the broader point of how enzyme activity is regulated by structural details of binding surfaces.

Reviewer #2

Evidence, reproducibility and clarity

The manuscript by Yue et al. investigates the molecular mechanisms by which MATCAP1, a metalloprotease enzyme, selectively modifies microtubules through detyrosination and ΔC2 modifications of α-tubulin. Using sophisticated microscopy-based enzymatic assays with newly developed fluorescent Fab probes specific for ΔY- and ΔC2-tubulin modifications, the authors demonstrate that MATCAP1 preferentially binds to microtubules in an expanded conformational state, achieved through preventing β-tubulin GTP hydrolysis (GMPCPP), Taxol treatment, or active kinesin-1 stepping, and exhibits remarkably long dwell times (>30 seconds) on these substrates. Through real-time visualization experiments in both cellular and reconstituted in vitro systems, they show that MATCAP1 sequentially removes amino acids from the α-tubulin C-terminal tail, generating ΔY modifications faster than ΔC2 modifications, and can operate independently of cytosolic carboxypeptidases. The findings significantly advance our

understanding of tubulin post-translational modification regulation by revealing that microtubule lattice conformation serves as a critical gating mechanism for enzymatic activity. This work fits into the broader "tubulin code" framework, where specific combinations of post-translational modifications create functionally distinct microtubule populations that selectively recruit different motor proteins and microtubule-associated proteins. The discovery that MATCAP1's activity is conformationally gated provides a molecular explanation for how cells might spatially and temporally control tubulin modifications, complementing recent structural studies and adding to the growing understanding of how mechanical and conformational states of the cytoskeleton influence biochemical processes.

The paper should be of high interest to the cytoskeleton and cell biology communities and I support publication after the authors address a few concerns below.

Response: We thank the reviewer for these positive comments.

Major points:

1. There is no explicit demonstration that the Fab probes do not bind to microtubules in the absence of MATCAP1 or VASH1/SVBP. It is critical, particularly for the ΔC2 Fab647 probe, to show that incubation for 10 minutes with the Fab but without added modifying enzyme results in negligible labeling. This would rule out two key confounders: (a) pre-existing ΔY or ΔC2 PTMs in the tubulin preparation, and (b) non-specific Fab probe binding.

Response: We thank the reviewer for this important comment. We believe that our original Figure 2D provides this critical control data for the ΔY-Fab, demonstrating that the Fab does not bind to microtubules in the absence of the detyrosination enzymes. In the revised version, we have carried out the requested experiments for the ΔC2-Fab. We now show that the ΔC2-Fab does not bind to microtubules in the absence of the MATCAP1 enzyme (**new Figure 2I**).

To make these critical controls more explicit, we have added representative images and kymographs showing negligible binding of both ΔY-Fab and ΔC2-Fab probes to microtubules in untransfected cell lysate controls (**new Figure EV2**). These data rule out the possibility of pre-existing PTMs in our tubulin preparation and non-specific Fab binding confounding our results and interpretations.

2. Figure 3: The non-saturation of ΔC2-Fab647 signal over time suggests potential issues with probe specificity and/or Fab probe affinity. Controls in which the ΔC2 probe is incubated with microtubules lacking any enzyme treatment should be shown, ideally on the same batch/preparation of tubulin as used in experimental samples. The specificity and occupancy properties of these probes (especially ΔC2-Fab647) should be clarified.

Response: As addressed in our response to the reviewer's point #1 (above), the controls in which Fab probes were incubated with microtubules lacking any enzyme treatment (using untransfected cell lysates) are provided in Figure 2D, the **new Figure 2I**, and the **new Figure**

EV2. We note that the HeLa tubulin used for both control and experimental samples was from the same batch and preparation and has been validated to lack pre-existing ΔY and ΔC2 PTMs (see Figure 10 of Thomas et al., 2025).

The specificity of the ΔY-Fab probe for ΔY-α-tubulin can be found in Yue et al 2023 and the specificity of the ΔC2-Fab probe for ΔC2-α-tubulin can be found in Figure 2F.

The occupancy and affinity of the Fab probes are illustrated in the supplemental figures. Figure EV4A,B (previously Figure S3A,B) shows that the presence of the ΔY-Fab probe does not affect the occupancy of the ΔC2-Fab probe. Figure EV4C,D (previously Figure S3C,D) shows that both probes display rapid binding to and unbinding from modified microtubules at the single molecule level. Figure EV4E-J (previously Figure S3E,F) shows that both ΔY-Fab and ΔC2-Fab probes rapidly saturate pre-modified microtubules. Thus, the non-saturation of the DC2-Fab probe when incubated with microtubules and low concentrations of MATCAP1 or for shorter periods of time reflects the enzymatic activity of MATCAP1 rather than probe binding kinetics.

3. It is unclear when and why the authors add MATCAP1 or VASH1/SVBP enzymes as cell lysates rather than as purified proteins. This raises some concerns - Lysates can contain endogenous enzymes, nucleases, or additional PTM-modifying activities that may confound the interpretation of results. It would be more rigorous to demonstrate key findings with purified MATCAP1 and VASH1/SVBP proteins, at least as a confirmatory step.

- The justification for using lysates rather than purified proteins should be clearly stated in the Results and rationales provided in the Methods. If purified proteins were limiting, this should be acknowledged, and the purity and concentration of MATCAP1 in lysates should be quantified and controlled across experiments.

Response: We thank the reviewer for raising this important point. Cell lysates are much easier to obtain than purified proteins, and their use in in vitro assays is well-established in the field (e.g. Cai et al 2007, Cai et al 2009, Norris et al 2014, Fenton et al 2021, Jijumon et al 2022, Torvi et al 2022, Yue et al 2023). However, we agree that their usage comes with some concerns. We started this study using cell lysate to examine MATCAP1 activity to validate of the probes, compare MATCAP1 activity to VASH1/SVBP, and examine MATCAP1 binding to Y-microtubules vs ΔY-microtubules (Figures 1-4). Even in these experiments, we considered the potential for lysate-induced artifacts. For example, in comparing the enzymatic properties of MATCAP1 to VASH1/SVBP, we included untransfected cell lysate as a control (e.g., Figures 2D, 4B, and **new Figures 2I and EV2**). We were careful to quantify the amount of MATCAP1 or VASH1/SVBP in the lysates and to report the concentration of enzyme added to each experiment.

Once we obtained preliminary data indicating that MATCAP1 binding is sensitive to the expanded vs compacted state of the microtubule lattice, we proceeded to demonstrate these key findings with purified MATCAP1 protein (Figure 5 and Figure EV6).

4. The major conclusions of the paper rest on rate measurements for fluorescently labeled MATCAP1 binding to microtubules with different PTM states. However, inspection of the kymographs shows that most particles appear stably bound before imaging starts, leading to some confusion. The authors must clarify in both the Results and Methods whether only particles landing de novo during image acquisition were counted when calculating landing rates. If so, the precise criteria used to define a "landing event" versus pre-bound molecules should be detailed. Given the use of cell lysates with fluorophore-conjugated Halo ligand, a negative control is essential: add Halo ligand to lysates from cells never transfected with (or expressing) MATCAP1, then perform the same TIRF binding and landing analysis. This would control for non-specific labeling of cellular proteins and verify that any observed binding is genuinely due to fluorescent MATCAP1, not other endogenous or labeled species. Without this clarification, it is difficult to interpret the main results of lattice preference as measured by landing rates, although some of this concern is alleviated by average fluorescence measurements as in Fig. 5.

Response: We thank the reviewer for these suggestions.

With respect to the suggestion that "The authors must clarify in both the Results and Methods whether only particles landing de novo during image acquisition were counted when calculating landing rates. If so, the precise criteria used to define a "landing event" versus pre-bound molecules should be detailed.": We have clarified in the Results and Methods sections that the landing rate includes all MATCAP1 binding events observed on the microtubules, specifically those which occurred before image acquisition and those that occurred during image acquisition.

With respect to the suggestion that "Given the use of cell lysates with fluorophore-conjugated Halo ligand, a negative control is essential: add Halo ligand to lysates from cells never transfected with (or expressing) MATCAP1, then perform the same TIRF binding and landing analysis.": We have carried out the requested experiment (**new Figure 4B**). We find that no microtubule binding was observed when using untransfected cell lysate containing Halo JFX554 ligand.

Minor comments:

1. Figure S4 examines MATCAP1 detachment from microtubules in response to buffer changes, but critical technical details are missing. The basis for calculating ionic strength (IS) across the different buffer conditions should be documented, including the formula used and all constituent ions considered. The abbreviation "IS" is not defined in the figure or legend and should be added.

Response: We thank the reviewer for raising this point and apologize for the omission of technical details in the original manuscript. In the revised manuscript, we have added the details of the ionic strength calculation to the Methods section. The definition of the abbreviation 'IS' has been added in the figure legend of Figure EV5 (previously Figure S4).

2. The interpretation that ATP-bound KIF5C can promote an expanded microtubule lattice, enhancing MATCAP1 binding, is important to the authors' conclusions. Yet, there are no data presented to demonstrate that the purified KIF5C used is functionally active as a motor. The manuscript should report a direct activity assay showing that the KIF5C preparation is capable of ATP-driven stepping along microtubules, e.g., via gliding or single-molecule motility assays. If not performed, a statement should be added to justify the assumption of activity.

Response: We thank the reviewer for this comment. Per the reviewer's suggestion, we carried out microtubule gliding assays with the same preparation of purified KIF5C(1-560)-Halo-TwinStrep protein that was used in Figure 5. The data demonstrate that the purified KIF5C is an active motor (**new Figure EV7**).

Significance

This is a nice paper that should be of interest to a broad community of cell biologists and biochemists interested in the cytoskeleton and tubulin code. The reagents and assays developed will also be of interest to this community.

Reviewer #3

Evidence, reproducibility and clarity (Required)):

This manuscript examines the mechanism of MATCAP1, a tubulin-modifying enzyme that removes residues from the C-terminal tails. The authors find that MATCAP1 generates both dY and dC2 tubulin using a combination of cell-based assays, in vitro assays with cell lysates, and in vitro assays with purified proteins. They find that MATCAP1 acts preferentially on microtubules in the expanded conformation, which is consistent with this team's recently-published work on VASH/SVBP, a tubulin-modifying assay that generated dY tubulin, suggesting a general mechanism for regulation of the dY and dC2 modifications. Overall, the manuscript is excellent, with high-quality data that strongly supports the central conclusions.

Concerns / Areas for Improvement

- The paper discusses the kinetics of MATCAP enzymatic activity in a few places. For example, in Figure 2, the authors show that the binding of their dY probe to microtubules is relatively fast (Fig. 2D), while the binding of their dC2 probe to microtubules is relatively slow (Fig. 2I). One obvious interpretation of this data is that the reaction is sequential: MATCAP first cleaves the Y (quickly) and then cleaves the E to generate dC2 (slowly). The Abstract and Introduction use the phrase "sequentially", although the authors do not make an explicit link between their data in Figure 2 and the concept of sequential cleavage. This is a minor writing issue.

Response: We thank the reviewer for these thoughtful and insightful comments. We have added new text (last paragraph on p. 6) to make an explicit link between the data in Figure 2

and the concept of sequential cleavage. We have also added a schematic of the sequential cleavage reaction (**new Figure EV3A**).

A more significant issue is how the data in Figure 2 interacts with the data in Figure 4. In Figure 4F, the binding of the dC2 probe is identical regardless of whether the microtubules were Y or dY to start with. I'm confused by this finding. If the microtubules are Y-MTs, then they need to first be cleaved to dY, then cleaved to dC2, and then the probe can bind.

Response: We also expected there to be a delay in ΔC2-Fab decoration of Y-microtubules compared to ΔY-microtubules and thus we also struggled with understanding the data in the previous Figure 4F (**now Figure 4H**). The reviewer's comments urged us undertake a careful review and re-analysis of our data. The previous data showed the mean fluorescence intensity of ΔC2-Fab on Y- and ΔY-microtubules over time. However, our review of the data showed that the results were confounded by day-to-day background variation and experimental noise. To address this, we re-analyzed the data to normalize of the mean fluorescence intensity of the ΔC2-Fab in each frame against the mean intensity measured in the first frame. This brings the analysis in line with the way the data are collected, analyzed, and reported in Figure 3D. Our **new Figure 4H** shows that the increase in mean fluorescence intensity of the ΔC2-Fab along Y-microtubules exhibits a slight lag compared to ΔY-microtubules in the presence of MATCAP1:

Original Figure 4F

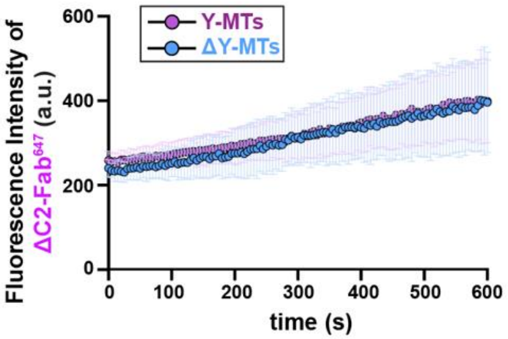

Revised Figure 4H

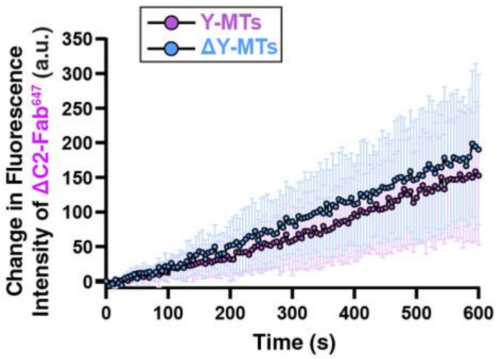

The data in Figure 4F can be explained a few ways. Option 1 is that the cleavage of the Y to generate the dY microtubules is really really really fast, such that it's not detectable in the binding kinetics of the dC2 probe. But this option is not consistent with Figure 2D and Figure 3D, which shows that the dY probe takes 10's to 100's of seconds to reach saturation. In other words, cleavage of the Y is fast, but it's not THAT fast.

To investigate this, we performed a simulation of the sequential enzymatic reactions (see figure below). Here, k_1 is the rate constant for the first reaction (Y → ΔY), and k_2 is the rate constant for the second reaction (ΔY → ΔC2). If the first reaction is much much faster than the second reaction (k_1/k_2 ratio = 50), then the curve for MATCAP1 activity starting with Y-MTs (Y → ΔC2, orange line) is similar to the curve for MATCAP1 activity starting with ΔY-MTs (ΔY → ΔC2, purple line). However, if the first reaction is only a bit faster than the second reaction (k_1/k_2 ratio

= 5), then the curve for MATCAP1 activity starting with Y-MTs (Y → ΔC2, green line) shows a significant delay compared to the curve for MATCAP1 activity starting with ΔY-MTs (ΔY → ΔC2, purple line).

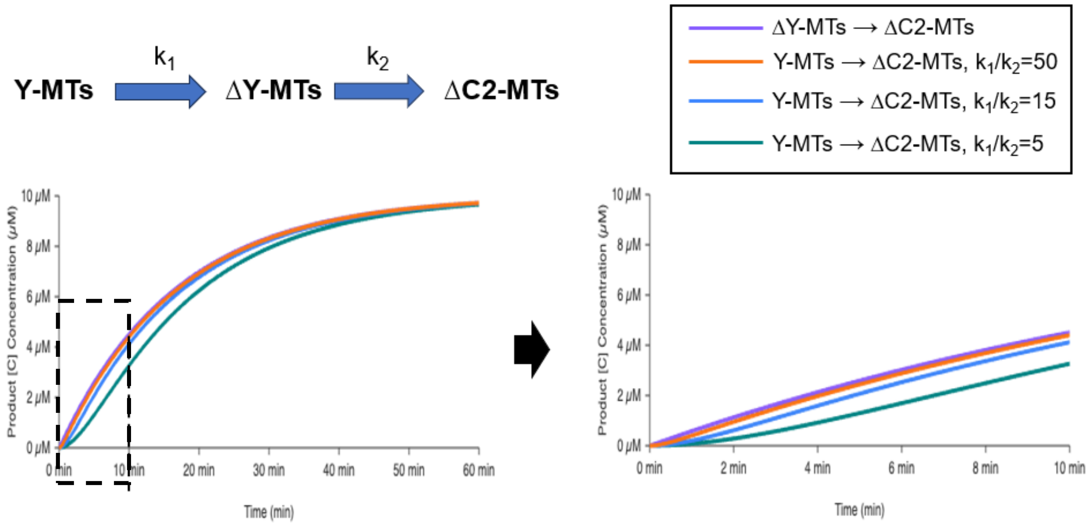

We used our data shown in Figure EV3B,C (previously Figure S2) to approximate the k_1/k_2 ratio. In this assay, the levels of ΔY and ΔC2 modifications on HeLa microtubules were measured following the addition of high concentrations (4.2 nM) of MATCAP1 during 1 h of imaging. The fluorescence intensity of the ΔY-Fab plateaus at around 5 min, while the fluorescence intensity of the ΔC2-Fab continues to increase throughout the 60 min imaging period. These results suggest that the k_1/k_2 ratio is greater than 12. If we use a k_1/k_2 ratio = 15 in the simulations, then the curve for MATCAP1 activity starting with Y-MTs (Y → ΔC2, blue line) shows a slight delay compared to the curve for MATCAP1 activity starting with ΔY-MTs (ΔY → ΔC2, purple line). Thus, the simulation results support our experimental findings (revised Figure 4H) showing that there is only a slight delay in the generation of ΔC2-microtubules when starting from ΔY-microtubules as compared to Y-microtubules.

Option 2 to explain Figure 4F could be that the binding of the dC2 probe is actually rather slow, such that the kinetics of dC2 probe binding are a measure only of the probe's binding kinetics, not the rate of generation of the epitope. However, if that's the case, then the data in Figure 2D and 3D is not sufficient to demonstrate the "MATCAP1 generates dY-MTs faster than dC2-MTs in vitro", because that conclusion interprets the slow binding of the dC2 probe as evidence of slow generation of epitope. In Figure S2, the authors measure the binding kinetics of the probes to pre-cleaved MTs. Indeed, the binding of the dC2 probe does appear slower than binding of the dY probe -- but also that the dC2 probe never reaches saturation, even after 1 hour of incubation.

Response: In the previous Figure S2 (now Figure EV3B,C), we did not use pre-cleaved microtubules. Rather, this experiment used HeLa microtubules and both probes to directly compare the ability of MATCAP1 to generate the ΔY and ΔC2 modifications. The point of Figure

EV3B,C is to examine DC2 generation at higher levels (4.2 nM) and longer times (60 min) of MATCAP1 enzyme than is shown in Figure 3 (1 nM of MATCAP1 over 10 min).

In the previous Figure S3 (now Figure EV4C-J), we measured the binding kinetics of the probes to pre-cleaved microtubules. On microtubules with low levels of modifications, both probes under rapid binding and unbinding events to their respective microtubules (Figure EV4C,D). On microtubules with high levels of modifications, both probes rapidly (within 2 min) saturate binding to their respective microtubules (Figure EV4I,J).

In the revised manuscript, we have rewritten the statement to highlight this property of the Fab probes and have included representative images showing Fab labeling of the pre-cleaved ΔY- and ΔC2-microtubules at 0 min and 10 min after Fab probe addition for clarification (new Figure EV4E,F).

I hope the paragraph above makes sense -- it's hard to describe kinetics w/out just writing down a reaction scheme. But I do believe there is an issue to resolve. Either the dC2 probe is very slow and can't reach saturation for some reason--in which case the title of Figure 3 is not support. Alternatively, the binding of the probe is not very slow, in which case Figure 4F doesn't make sense to me. Perhaps the authors would benefit from writing down the kinetic scheme for a sequential cleavage reaction, including probe binding. The curves they have generated (e.g. in Figure S2B) might then be sufficient to see what's going on.

Response: We again thank the reviewer for these thoughtful and insightful comments. In the revised manuscript, we have included a kinetic scheme illustrating the sequential cleavage reaction of MATCAP1 on microtubules including probe binding (**new Figure EV3A**).

- Some experiments in the paper are performed with MATCAP1 in cell lysates while other experiments used purified proteins. The rationale for which experiments used which system were unclear to me. At key points, the switch to purified protein was described as a way to exclude potential artifacts that come from the lysate (e.g. another MAP that is present, etc). But why is that a concern in some experiments but not others? Maybe the lysate is actually advantageous? The paper would benefit from a clearer rationale and explanation for the switch between the different systems.

Response: We thank the reviewer for raising this important point. Cell lysates are much easier to obtain than purified proteins, and their use in in vitro assays is well-established in the field (e.g. Cai et al 2007, Cai et al 2009, Norris et al 2014, Fenton et al 2021, Jijumon et al 2022, Torvi et al 2022, Yue et al 2023). However, we agree that their usage comes with some concerns. We started this study using cell lysate to examine MATCAP1 activity to validate of the probes, compare MATCAP1 activity to VASH1/SVBP, and examine MATCAP1 binding to Y-microtubules vs ΔY-microtubules (Figures 1-4). Even in these experiments, we considered the potential for lysate-induced artifacts. For example, in comparing the enzymatic properties of MATCAP1 to VASH1/SVBP, we included untransfected cell lysate as a control (e.g., Figures 2D, 4B, and **new Figures 2I and EV2**). We were careful to quantify the amount of MATCAP1 or

VASH1/SVBP in the lysates and to report the concentration of enzyme added to each experiment.

Once we obtained preliminary data indicating that MATCAP1 binding is sensitive to the expanded vs compacted state of the microtubule lattice, we proceeded to demonstrate these key findings with purified MATCAP1 protein (Figure 5 and Figure EV6).

- Similarly, the authors use a catalytically-inactive MATCAP late in the paper (Figure 5)--could the E281Q construct be used elsewhere to measure, e.g., in the experiments using Y vs. dY-MTs? This suggestion is not essential but might strengthen the idea that the Y-status of the lattice is not important to recruitment.

Response: We thank the reviewer for this suggestion. We carried out new experiments to examine the microtubule binding of WT and E281Q mNG-MATCAP1 (**new Figure 4D,E**). We find that both WT and E281Q mNG-MATCAP1 show similar affinities for Y-microtubules and ∆Y-microtubules. We agree that these results strengthen our conclusion that the microtubule binding behavior of MATCAP1 is not affected by the tyrosination state of the lattice.

- In Figure 5D, the results show that MATCAP binding to GMPCPP microtubules is higher relative to Taxol-stabilized microtubules, even though (presumably) both microtubules are fully expanded. Does this finding suggest there are other structural features besides expansion that are at work? What could those features be?

Response: The reviewer raises an excellent point. We have added text to the Discussion noting that several differences have been reported between GMPCPP microtubules and Taxol-stabilized microtubules that could affect the binding and activity of microtubule-modifying enzymes. For example, GMPCPP microtubules are more expanded, contain more protofilaments, and exhibit a more extensive degree of positive twist (Alushin et al 2014, Zhang et al 2015, LaFrance et al 2019). All of these features could potentially affect MATCAP1 binding. Further investigation is needed to determine whether these properties act synergistically with lattice expansion to contribute to the observed differences in MATCAP1 microtubule affinity.

Reviewer #3 (Significance (Required)):

Overall, only the first of the concerns above is important relative to the experimental support for the authors' conclusions, namely the conclusion stated in the title of Figure 3. Otherwise, it's an outstanding paper that will make an important contribution to our understanding of how the tubulin code is regulated.

Literature Cited

Alushin, G. M., Lander, G. C., Kellogg, E. H., Zhang, R., Baker, D. and Nogales, E. (2014). High-resolution microtubule structures reveal the structural transitions in alpha/beta-tubulin upon GTP hydrolysis. *Cell*, 157, 1117-1129.

Cai, D., Verhey, K. J., & Meyhöfer, E. (2007). Tracking single Kinesin molecules in the cytoplasm of mammalian cells. *Biophysical journal*, *92*(12), 4137–4144.

Cai, D., McEwen, D. P., Martens, J. R., Meyhofer, E., & Verhey, K. J. (2009). Single molecule imaging reveals differences in microtubule track selection between Kinesin motors. *PLoS biology*, *7*(10), e1000216.

Fenton, A. R., Cason, S. E., & Holzbaur, E. L. F. (2023). Single-Molecule Studies of Motor Adaptors Using Cell Lysates. *Methods in molecular biology 2623*, 97–111.

Hammond, J. W., Cai, D., Blasius, T. L., Li, Z., Jiang, Y., Jih, G. T., Meyhofer, E., & Verhey, K. J. (2009). Mammalian Kinesin-3 motors are dimeric in vivo and move by processive motility upon release of autoinhibition. *PLoS biology*, *7*(3), e72.

Jijumon, A. S., Bodakuntla, S., Genova, M., Bangera, M., Sackett, V., Besse, L., Maksut, F., Henriot, V., Magiera, M. M., Sirajuddin, M., & Janke, C. (2022). Lysate-based pipeline to characterize microtubule-associated proteins uncovers unique microtubule behaviours. *Nature cell biology*, *24*(2), 253–267.

LaFrance, B. J., Roostalu, J., Henkin, G., Greber, B. J., Zhang, R., Normanno, D., McCollum, C. O., Surrey, T. and Nogales, E. (2022). Structural transitions in the GTP cap visualized by cryo-electron microscopy of catalytically inactive microtubules. *Proc Natl Acad Sci U S A*, 119, e2114994119.

Landskron, L., Bak, J., Adamopoulos, A., Kaplani, K., Moraiti, M., van den Hengel, L. G., Song, J. Y., Bleijerveld, O. B., Nieuwenhuis, J., Heidebrecht, T., Henneman, L., Moutin, M. J., Barisic, M., Taraviras, S., Perrakis, A. and Brummelkamp, T. R. (2022). Posttranslational modification of microtubules by the MATCAP detyrosinase. *Science*, 376, eabn6020.

Nieuwenhuis, J., Adamopoulos, A., Bleijerveld, O. B., Mazouzi, A., Stickel, E., Celie, P., Altelaar, M., Knipscheer, P., Perrakis, A., Blomen, V. A. and Brummelkamp, T. R. (2017). Vasohibins encode tubulin detyrosinating activity. *Science*, 358, 1453-1456.

Norris, S. R., Soppina, V., Dizaji, A. S., Schimert, K. I., Sept, D., Cai, D., Sivaramakrishnan, S., & Verhey, K. J. (2014). A method for multiprotein assembly in cells reveals independent action of kinesins in complex. *The Journal of cell biology*, *207*(3), 393–406.

Thomas, E. C., Yue, Y., Pimm, M. L., Hotta, T., Ohi, R. and Verhey, K. J. (2025). Purification, fluorescent labelling, and detyrosination of mammalian cell tubulin for biochemical assays. *Cytoskeleton,* 1–19.

Torvi, J. R., Wong, J., Serwas, D., Moayed, A., Drubin, D. G., & Barnes, G. (2022). Reconstitution of kinetochore motility and microtubule dynamics reveals a role for a kinesin-8 in establishing end-on attachments. *eLife*, *11*, e78450.

Yue, Y., Hotta, T., Higaki, T., Verhey, K. J. and Ohi, R. (2023). Microtubule detyrosination by VASH1/SVBP is regulated by the conformational state of tubulin in the lattice. *Curr Biol*, 33, 4111-4123.

Zhang, R., LaFrance, B. and Nogales, E. (2018). Separating the effects of nucleotide and EB binding on microtubule structure. *Proc Natl Acad Sci U S A*, 115, E6191-E6200.

1st Editorial Decision 27th Feb 2026

Dear Kristen,

Thank you for submitting your fully revised Review Commons manuscript to The EMBO Journal. I apologise for the delays in the assessment process due to delays in reviewer report submission and the standard revision checks that we have to perform on revised manuscripts at the pre-decision stage.

I have now received positive re-assessment from the original reviewer #2. I have gone through your responses to the comments by other reviewers and find them generally reasonable. Therefore, there now remain only a few editorial points that need to be addressed before I can extend official acceptance of the manuscript:

1. Please submit a complete author checklist, which you can download from our author guidelines (https://media.springernature.com/original/springer-cms/rest/v1/content/27825796/data/v1). Please insert information in the checklist that is also reflected in the manuscript. The completed author checklist will also be part of the Review Process File.
2. As part of the EMBO Press transparent editorial process, The EMBO Journal will publish online a Peer Review File to accompany accepted manuscripts. This file will be published in conjunction with your paper and will include the anonymous referee reports, your point-by-point response and all pertinent correspondence relating to the manuscript, including decision letters. Please note that the Author Checklist will be published at the end of the Peer Review File.
Please let us know if you want to remove or not any figures or data from the Peer Review File prior to publication. Please note that retaining unpublished data in the Peer Review File means that these count as published and that the Peer Review File would need to be referenced in future publications.
3. At EMBO Press we ask authors to provide source data for the main manuscript figures. You will receive a separate email with instructions for providing source data with your revised manuscript, including how to upload and organise the files.
4. Please remove the abbreviations list from the manuscript text file.
5. We are missing the ORCID iD for the co-corresponding author Ryoma Ohi. In order to link the ORCID iD to the account in our manuscript tracking system, the author in question has to do the following:
- Click the 'Modify Profile' link at the bottom of your homepage in our system.
- On the next page you will see a box halfway down the page titled ORCID*. Below this box is red text reading 'To Register/Link to ORCID, click here'. Please follow that link: you will be taken to ORCID where you can log in to your account (or create an account if you don't have one)
- You will then be asked to authorise Wiley to access your ORCID information. Once you have approved the linking, you will be brought back to our manuscript system.
Unfortunately, we cannot do this linking on the author's behalf for security reasons.
6. CRediT has replaced the traditional author contributions section because it offers a systematic, machine-readable author contributions format that allows for more effective research assessment. Please remove the Authors Contributions from the manuscript and use the free text boxes beneath each contributing author's name in our online submission system to add specific details on the author's contribution. More information is available in our guide to authors.
7. Please merge the "Funding" section with "Acknowledgements".
8. Please rename "Conflict of Interest Statement" into "Disclosure and competing interests statement".
9. We require a Data Availability Section at the end of Materials and Methods. As far as I can see, no data deposition in external databases is needed for this paper. If I am correct, then please state in this section: This study includes no data deposited in external repositories. Further information can be found at https://link.springer.com/partners/embo-press/editorial-policies#Data%20availability%20statement.
10. Individual panels of Figures EV5 and EV7 are not mentioned in the manuscript text; please add the corresponding callouts.
11. Please check the order of the figure callouts in the manuscript text: currently, Fig EV5 is called out before Fig EV4C-J.
12. Please provide the exact p values in the legends of figures 5B, D, F, H; 6C, EV6 C, E.
13. Please update the references according to The EMBO Journal style - where there are more than 10 authors on a paper, the first 10 should be listed, followed by 'et al.'
14. Papers published in The EMBO Journal are accompanied online by a 'Synopsis' to enhance discoverability of the manuscript. It consists of A) a short (1-2 sentences) summary of the findings and their significance, B) 3-4 bullet points highlighting key results and C) a synopsis image that is 550x300-600 pixels large (width x height, jpeg or png format). You can either show a model or key data in the synopsis image. Please note that the image size is rather small and that text needs to be readable at the final size. Please send us this information together with the revised manuscript.

Please let me know if you have any questions regarding any of these points. You can use the link below to upload the revised files.

Thank you again for giving us the chance to consider your manuscript for The EMBO Journal. I look forward to receiving the final version!

With kind regards,

Ieva

Ieva Gailite, PhD
Senior Scientific Editor
The EMBO Journal
Meyerhofstrasse 1
D-69117 Heidelberg
Tel: +4962218891309
i.gailite@embojournal.org

Instructions for preparing your revised manuscript:

Read our guidance for manuscript revisions and related editorial policies: https://link.springer.com/journal/44318/submission-guidelines#cms-Revised-submissions

Revision to The EMBO Journal should be submitted online within 90 days, unless an extension has been requested and approved by the editor; please click on the link below to submit the revision online before 28th May 2026:

Link Not Available

Referee #2:

I am satisfied that the authors have fully addressed my concerns regarding experimental rigor and reproducibility. The addition of the 'no-enzyme' negative controls explicitly demonstrates the specificity of the Fab probes, and the new kinetic data on pre-cleaved microtubules effectively resolves the ambiguity regarding signal saturation, confirming that the probes are reliable reporters of enzymatic activity. Furthermore, the use of purified proteins to validate the lattice-preference mechanism significantly strengthens the study's central conclusions. The manuscript is now very solid in my opinion, and I support its publication in the EMBO J.

***
Rev_Com_number: RC-2025-03171
New_manu_number: EMBOJ-2025-123443-T
Corr_author: Verhey
Title: MATCAP1 preferentially binds an expanded tubulin conformation to generate detyrosinated and ΔC2 a-tubulin

The authors addressed the remaining editorial issues.

1st Revision - Editorial Decision 26th Mar 2026

Dear Kristen,

Thank you for incorporating the final editorial and formatting requests in the manuscript. I apologise for the slow process from our side due to the large number of revised submissions that we currently receive. I am now pleased to inform you that your manuscript has been accepted for publication. Congratulations with a nice study!

Before we forward your manuscript to our publishers, we would like to propose some edits in the manuscript abstract and synopsis. I have also written a short blurb that will accompany the title of your manuscript in our online table of contents. Please take a look at the proposed text changes in the attached text file and let me know if any corrections are needed.

Your manuscript will be processed for publication by EMBO Press. It will be copy edited and you will receive page proofs prior to publication. Please note that you will be contacted by Springer Nature Author Services to complete licensing and payment information.

You may qualify for financial assistance for your publication charges - either via a Springer Nature fully open access agreement or an EMBO initiative. Check your eligibility: https://link.springer.com/journal/44318/how-to-publish-with-us

Should you be planning a Press Release on your article, please get in contact with embo_production@springernature.com as early as possible in order to coordinate publication and release dates.

If you have any questions, please do not hesitate to contact the Editorial Office or me directly. Thank you for this interesting contribution to The EMBO Journal!

Best wishes,

Ieva

Ieva Gailite, PhD
Senior Scientific Editor
The EMBO Journal
Meyerhofstrasse 1
D-69117 Heidelberg
Tel: +4962218891309
i.gailite@embojournal.org

*****
Please note that it is The EMBO Journal policy for the transcript of the editorial process (containing referee reports and your response letters) to be published as an online supplement to each paper. If you should prefer removal of any referee-only figures included in the point-by-point response(s), e.g. because they may still be used for future publication or because they have been reproduced from published work by others, please do let us know immediately via response email.
More information is available here: https://link.springer.com/partners/embo-press/editorial-policies#Peer%20review
*****

***
Rev_Com_number: RC-2025-03171
New_manu_number: EMBOJ-2025-123443R
Corr_author: Verhey
Title: MATCAP1 preferentially binds an expanded tubulin conformation to generate detyrosinated and ΔC2 a-tubulin

EMBO Press Author Checklist

Corresponding Author Name: Kristen Verhey	
Journal Submitted to: EMBO Journal	
Manuscript Number: EMBOJ-2025-123443-T	

USEFUL LINKS FOR COMPLETING THIS FORM
The EMBO Journal - Author Guidelines
EMBO Reports - Author Guidelines
Molecular Systems Biology - Author Guidelines
EMBO Molecular Medicine - Author Guidelines

Reporting Checklist for Life Science Articles (updated January
This checklist is adapted from Materials Design Analysis Reporting (MDAR) Checklist for Authors. MDAR establishes a minimum set of requirements in transparent reporting in the life sciences (see Statement of Task: 10.31222/osf.io/9sm4x). Please follow the journal's guidelines in preparing your
Please note that a copy of this checklist will be published alongside your article.

Abridged guidelines for figures
1. Data
The data shown in figures should satisfy the following conditions:
- the data were obtained and processed according to the field's best practice and are presented to reflect the results of the experiments in an accurate and unbiased manner.
- ideally, figure panels should include only measurements that are directly comparable to each other and obtained with the same assay.
- plots include clearly labeled error bars for independent experiments and sample sizes. Unless justified, error bars should not be shown for technical
- if n<5, the individual data points from each experiment should be plotted. Any statistical test employed should be justified.
- Source Data should be included to report the data underlying figures according to the guidelines set out in the authorship guidelines on Data

2. Captions
Each figure caption should contain the following information, for each panel where they are relevant:
- a specification of the experimental system investigated (eg cell line, species name).
- the assay(s) and method(s) used to carry out the reported observations and measurements.
- an explicit mention of the biological and chemical entity(ies) that are being measured.
- an explicit mention of the biological and chemical entity(ies) that are altered/varied/perturbed in a controlled manner.
- the exact sample size (n) for each experimental group/condition, given as a number, not a range;
- a description of the sample collection allowing the reader to understand whether the samples represent technical or biological replicates (including how many animals, litters, cultures, etc.).
- a statement of how many times the experiment shown was independently replicated in the laboratory.
- definitions of statistical methods and measures:
  - common tests, such as t-test (please specify whether paired vs. unpaired), simple χ2 tests, Wilcoxon and Mann-Whitney tests, can be unambiguously identified by name only, but more complex techniques should be described in the methods section;
  - are tests one-sided or two-sided?
  - are there adjustments for multiple comparisons?
  - exact statistical test results, e.g., P values = x but not P values < x;
  - definition of 'center values' as median or average;
  - definition of error bars as s.d. or s.e.m.

Please complete ALL of the questions below.
Select "Not Applicable" only when the requested information is not relevant for your study.

Materials

Newly Created Materials	Information included in the manuscript?	In which section is the information available? (Reagents and Tools Table, Materials and Methods, Figures, Data Availability Section)
New materials and reagents need to be available; do any restrictions apply?	Not Applicable	

Antibodies	Information included in the manuscript?	In which section is the information available? (Reagents and Tools Table, Materials and Methods, Figures, Data Availability Section)
For **antibodies** provide the following information:<br>- Commercial antibodies: RRID (if possible) or supplier name, catalogue number and or/clone number<br>- Non-commercial: RRID or citation	Yes	Reagents and Tools Table.

DNA and RNA sequences	Information included in the manuscript?	In which section is the information available? (Reagents and Tools Table, Materials and Methods, Figures, Data Availability Section)
**Short novel DNA or RNA including primers, probes**: provide the sequences.	Yes	Materials and Methods

Cell materials	Information included in the manuscript?	In which section is the information available? (Reagents and Tools Table, Materials and Methods, Figures, Data Availability Section)
**Cell lines:** Provide species information, strain. Provide accession number in repository **OR** supplier name, catalog number, clone number, and/**OR** RRID.	Yes	Reagents and Tools Table.
**Primary cultures:** Provide species, strain, sex of origin, genetic modification status.	Not Applicable	
Report if the cell lines were recently **authenticated** (e.g., by STR profiling) and tested for mycoplasma contamination.	Yes	Materials and Methods

Experimental animals	Information included in the manuscript?	In which section is the information available? (Reagents and Tools Table, Materials and Methods, Figures, Data Availability Section)
**Laboratory animals or Model organisms:** Provide species, strain, sex, age, genetic modification status. Provide accession number in repository **OR** supplier name, catalog number, clone number, **OR** RRID.	Not Applicable	
**Animal observed in or captured from the field:** Provide species, sex, and age where possible.	Not Applicable	
Please detail **housing and husbandry conditions**.	Not Applicable	

Plants and microbes	Information included in the manuscript?	In which section is the information available? (Reagents and Tools Table, Materials and Methods, Figures, Data Availability Section)
**Plants:** provide species and strain, ecotype and cultivar where relevant, unique accession number if available, and source (including location for collected wild specimens).	Not Applicable	
**Microbes:** provide species and strain, unique accession number if available, and source.	Not Applicable	

Human research participants	Information included in the manuscript?	In which section is the information available? (Reagents and Tools Table, Materials and Methods, Figures, Data Availability Section)
If collected and within the bounds of privacy constraints report on age, sex and gender or ethnicity for all study participants.	Not Applicable	

Core facilities	Information included in the manuscript?	In which section is the information available? (Reagents and Tools Table, Materials and Methods, Figures, Data Availability Section)
If your work benefited from core facilities, was their service mentioned in the acknowledgments section?	Not Applicable	

Design

Study protocol	Information included in the manuscript?	In which section is the information available? (Reagents and Tools Table, Materials and Methods, Figures, Data Availability Section)
If study protocol has been **pre-registered, provide DOI in the manuscript**. For clinical trials, provide the trial registration number **OR** cite DOI.	Not Applicable	
Report the **clinical trial registration number** (at ClinicalTrials.gov or equivalent), where applicable.	Not Applicable	

Laboratory protocol	Information included in the manuscript?	In which section is the information available? (Reagents and Tools Table, Materials and Methods, Figures, Data Availability Section)
Provide DOI OR other citation details if **external detailed step-by-step protocols** are available.	Not Applicable	

Experimental study design and statistics	Information included in the manuscript?	In which section is the information available? (Reagents and Tools Table, Materials and Methods, Figures, Data Availability Section)
Include a statement about **sample size** estimate even if no statistical methods were used.	Yes	Figure legends
Were any steps taken to minimize the effects of subjective bias when allocating animals/samples to treatment (e.g. **randomization procedure**)? If yes, have they been described?	Not Applicable	
Include a statement about **blinding** even if no blinding was done.	Not Applicable	
Describe **inclusion/exclusion criteria** if samples or animals were excluded from the analysis. Were the criteria pre-established?<br><br>If sample or data points were omitted from analysis, report if this was due to attrition or intentional exclusion and provide justification.	Yes	Materials and Methods
For every figure, are **statistical tests** justified as appropriate? Do the data meet the assumptions of the tests (e.g., normal distribution)? Describe any methods used to assess it. Is there an estimate of variation within each group of data? Is the variance similar between the groups that are being statistically compared?	Yes	Figure legends

Sample definition and in-laboratory replication	Information included in the manuscript?	In which section is the information available? (Reagents and Tools Table, Materials and Methods, Figures, Data Availability Section)
In the figure legends: state number of times the experiment was **replicated** in laboratory.	Yes	Figure legends
In the figure legends: define whether data describe **technical or biological replicates**.	Yes	The biological replicates can be found in the Figure legends.

Ethics

Ethics	Information included in the manuscript?	In which section is the information available? (Reagents and Tools Table, Materials and Methods, Figures, Data Availability Section)
Studies involving **human participants**: State details of **authority granting ethics approval** (IRB or equivalent committee(s), provide reference number for approval.	Not Applicable	
Studies involving **human participants**: Include a statement confirming that **informed consent** was obtained from all subjects and that the experiments conformed to the principles set out in the WMA Declaration of Helsinki and the Department of Health and Human Services Belmont Report.	Not Applicable	
Studies involving **human participants: For** publication of **patient photos**, include a statement confirming that consent to publish was obtained.	Not Applicable	
Studies involving experimental **animals**: State details of **authority granting ethics approval** (IRB or equivalent committee(s), provide reference number for approval. Include a statement of compliance with ethical regulations.	Not Applicable	
Studies involving **specimen and field samples:** State if relevant **permits** obtained, provide details of authority approving study; if none were required, explain why.	Not Applicable	

Dual Use Research of Concern (DURC)	Information included in the manuscript?	In which section is the information available? (Reagents and Tools Table, Materials and Methods, Figures, Data Availability Section)
Could your study fall under dual use research restrictions? Please check biosecurity documents and list of **select agents and toxins** (CDC): https://www.selectagents.gov/sat/list.htm	Not Applicable	
If you used a select agent, is the security level of the lab appropriate and reported in the manuscript?	Not Applicable	
If a study is subject to dual use research of concern regulations, is the name of the **authority granting approval and reference number** for the regulatory approval provided in the manuscript?	Not Applicable	

Reporting

The MDAR framework recommends adoption of discipline-specific guidelines, established and endorsed through community initiatives. Journals have their own policy about requiring specific guidelines and recommendations to complement MDAR.

Adherence to community standards	Information included in the manuscript?	In which section is the information available? (Reagents and Tools Table, Materials and Methods, Figures, Data Availability Section)
State if relevant guidelines or checklists (e.g., **ICMJE, MIBBI, ARRIVE, PRISMA**) have been followed or provided.	Yes	MIBBI information is available in the Materials and Methods section. ICMJE information is available in the Author Contributions and Disclosure and Competing Interests Statement sections.
For **tumor marker prognostic studies,** we recommend that you follow the **REMARK** reporting guidelines (see link list at top right). See author guidelines, under 'Reporting Guidelines'. Please confirm you have followed these guidelines.	Not Applicable	
For **phase II and III randomized controlled trials**, please refer to the **CONSORT** flow diagram (see link list at top right) and submit the CONSORT checklist (see link list at top right) with your submission. See author guidelines, under 'Reporting Guidelines'. Please confirm you have submitted this list.	Not Applicable	

Data Availability

Data availability	Information included in the manuscript?	In which section is the information available? (Reagents and Tools Table, Materials and Methods, Figures, Data Availability Section)
Have **primary datasets** been deposited according to the journal's guidelines (see 'Data Deposition' section) and the respective accession numbers provided in the Data Availability Section?	Yes	Source data
Were **human clinical and genomic datasets** deposited in a public access-controlled repository in accordance to ethical obligations to the patients and to the applicable consent agreement?	Not Applicable	
Are **computational models** that are central and integral to a study available without restrictions in a machine-readable form? Were the relevant accession numbers or links provided?	Not Applicable	
If publicly available data were reused, provide the respective **data citations in the reference list**.	Not Applicable	
